# Flipper dendrimers

**DOI:** 10.1039/d5sc09248e

**Published:** 2026-02-20

**Authors:** Nerea Gonzalez-Sanchis, Felix Bayard, Juan Manuel García-Arcos, Tithi Mandal, Aurelien Roux, Naomi Sakai, Stefan Matile

**Affiliations:** a Department of Organic Chemistry, University of Geneva Geneva Switzerland stefan.matile@unige.ch www.unige.ch/sciences/chiorg/matile/ +41 22 379 6523; b National Centre of Competence in Research (NCCR) Molecular Systems Engineering Basel Switzerland; c Department of Biochemistry, University of Geneva Geneva Switzerland; d Swiss Institute for Experimental Cancer Research (ISREC), School of Life Sciences, Swiss Federal Institute of Technology Lausanne (EPFL) Lausanne Switzerland

## Abstract

Fluorescent flippers have been introduced as small-molecule probes to image physical forces within cell membranes. Despite their popularity and much effort, significant improvements of performance have not been reported since their first design. Now, almost a decade after their introduction, we disclose flipper dendrimers that address the main practical problem with flipper probes: phototoxicity. Flipper dendrimers provide much stronger fluorescence in cells while maintaining responsiveness to changes in membrane tension. This increased effective brightness enables imaging at almost one order of magnitude lower laser power to generate the same fluorescence intensity, thereby reducing phototoxicity and allowing longer monitoring of biological processes. This breakthrough is achieved using large peptide dendrimers that maximize deliverability as Israelachvili-inspired inverted cones. Peptide dendrimers and hydrophobic interfacers modulate fluorescence lifetime and plasma membrane targeting by controlling probe orientation, interdomain distribution, intermembrane transfer and internalization. This supramolecular chemistry strategy to improve performance by engineering probe integration into the environment, rather than the mechanophore itself, is generally applicable.

## Abstract 2

Shifting focus from mechanophore engineering to deliverability and interfacing affords fluorescent probes with higher effective brightness and lower phototoxicity for the imaging of membrane tension in living cells over longer periods of time.

## Graphical abstract

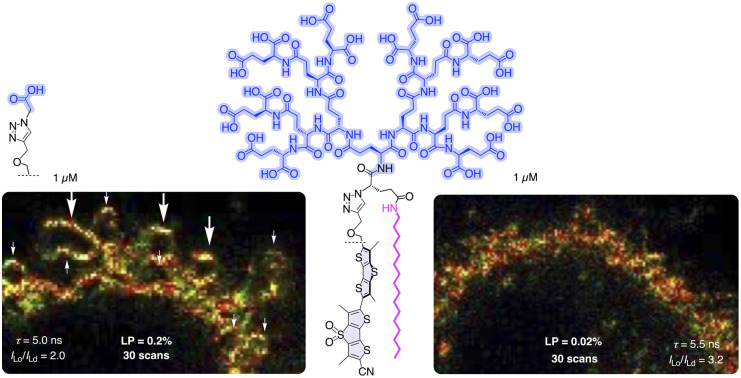

Distinct among modern small-molecule membrane probes,^1–14^ fluorescent flippers have been made to image membrane order and tension changes in living systems, in equilibrium in the ground state.^15^ After the introduction of the bioinspired concept^16–18^ of planarizable push–pull probes in 2012,^19^ the original Flipper-TR 1 became available in 2016 (Fig. 1).^20^ Flipper-TR 1 is constructed around twisted dithienothiophene dimers.^15^ Their planarization under mechanical compression by ordered membranes in the ground state increases conjugation and turns on a push–pull dipole (Fig. 1a and b). This red shifts excitation maxima and increases fluorescence intensity and lifetimes, without affecting the emission maxima, which instead are sensitive to the polarity of the environment.^15,21^ The dependence of fluorescence lifetime on membrane order allowed us to image membrane tension using fluorescence lifetime imaging microscopy (FLIM).^22^ Namely, application of tension or compression to biological membranes composed of various lipids promotes or reduces lipid phase separation to form ordered domains, respectively (Fig. 1c and d). Since Flipper-TR 1 is more emissive in the ordered domains, such changes in phase behavior result in an increase or decrease in fluorescent lifetime, which can be imaged using FLIM.^15^ With this scaffold, a small collection of flippers has been constructed to specifically target any membrane of interest (MOI) within cells for tension imaging.^15^

**Fig. 1:**
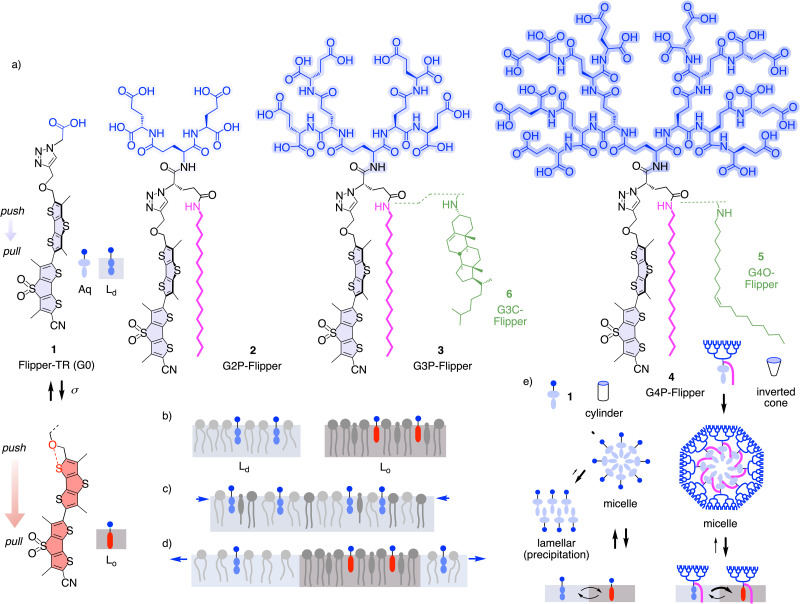
(a) The dendrimer-free original Flipper-TR 1 in twisted (blue) and planar conformation (red), the palmitoylated (P) flipper dendrimers 2–4 composed of 4, 8 and 16 glutamates (E) providing 4, 8 and 16 peripheral carboxylates, and flipper dendrimers 5 and 6 with oleyl (O) and cholesteryl amide (C) interfacers. (b–d) Schematic presentation of twisted (blue) and planar flipper probes (red) in (b) *L*_d_ and *L*_o_ membranes and in biological membranes under (c) decreasing and (d) increasing membrane tension. (e) Delivery mechanism of the cylindrical original G0 1 (left) and the inverted conical G4P 4 to *L*_d_ and *L*_o_ membrane domains.

The biggest weakness of the original flipper probes in practice is their phototoxicity.^23^ This is unproblematic for single scans of living systems by FLIM, but it prevents imaging of physical forces in biological processes for longer periods of time. Since modifications of the mechanophore did not solve the problem,^24,25^ we recently shifted attention to the integration of flippers into their environment.^7,26,27^ Palmitoylation (P), envisioned to shift partitioning from liquid-disordered (*L*_d_) to liquid-ordered (*L*_o_) and solid-ordered (*S*_o_) membrane microdomains (Fig. 1), afforded mostly insoluble material.^28^ Compensation with second-generation (G2) glutamate dendrimers in G2P-Flipper 2 restored solubility, but the performance of 2 in cells was much weaker than the original G0 1 (Fig. 2f).^29^ However, we noticed that the effective brightness of G2P 2 could exceed that of 1 in biomimetic model membranes under optimized conditions.^29^ This observation and the significance of flipper probes in biology encouraged us to synthesize flippers that are equipped with increasingly respectable peptide^30–39^ dendrimers,^30–56^ from G3P 3 to a majestic fourth-generation G4P-Flipper 4 with 16 glutamates and 16 formal^57^ negative charges. Peptide dendrimers were selected because, unlike linear peptides,^7,58^ they are sturdy spheres without secondary structural complications from conformational flexibility.^30–39^ They were expected to improve the probe's deliverability^15,59–64^ and thus the effective brightness because, according to Israelachvili rules, inverted-cone amphiphiles assemble into soluble micelles in water rather than forming precipitating lamellar aggregates (Fig. 1e).^65–67^ Aggregation-induced quenching in flipper micelles is known and beneficial to minimize background and work without washing.^15^ Upon binding to the membrane, these non-fluorescent micelles disassemble, and the fluorescence turns on.^7,15,59–61^ The anionic dendrimers should end up extending like big buoys on the membrane surface, somehow reminiscent of the structure of biological membrane proteins like α-hemolysin.^64^

**Fig. 2:**
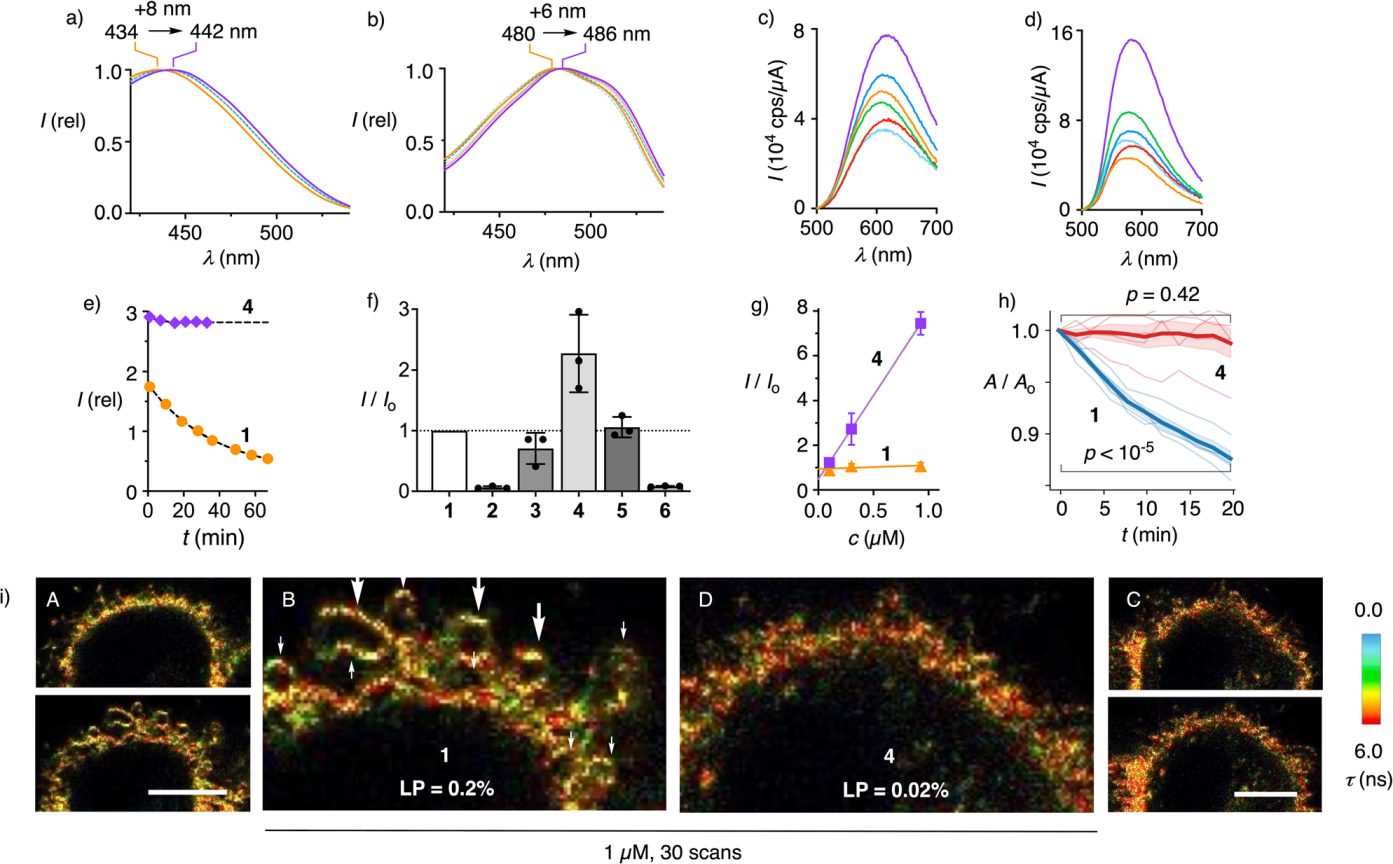
Flipper dendrimers in (a and c) *L*_d_ LUVs, (b and d) *L*_o_ LUVs, (e) PBS, and (f–i) HK cells. (a and b) Normalized excitation (*λ*_em_ = 600 nm) and (c and d) not normalized emission spectra (*λ*_ex_ = 420 nm) of 1 (orange), 2 (green), 3 (blue), 4 (purple), 5 (red), and 6 (cyan, all 100 nM) in (a and c) *L*_d_ DOPC and (b and d) *L*_o_ SM/CL 7 : 3 LUVs (10 mM tris 100 mM NaCl buffer, pH 7.4). (e) Fluorescence intensity with time for 1 (orange circles) and 4 (purple diamonds; both 1 µM, *λ*_ex_/*λ*_em_ = 434/650 nm) in PBS at 37 °C. (f) Relative fluorescence intensity of HK cells incubated with 1–6 (all 0.3 µM, >0.5 h) in FluoroBrite DMEM at identical laser power (LP), compared to that with 1 (*I*_0_). (g) The same for various concentrations of 1 and 4 after 1 h incubation. (h) Cell area *A* relative to the initial *A*_0_ during irradiation of HK cells incubated with 1 (blue) and 4 (red) at LP adjusted to comparable intensity (mean in bold, weak lines individual time lapse), *t*-test *p* values are between time point 0 and time point 11 (20 minutes at 2 min per frame). (i) The first (A and C, top) and the 30th scan (A and C, bottom; B and D zoom) of FLIM images of HK cells incubated with (A and B) 1 and (C and D) 4 (both 1 µM) at (A and B) LP = 0.2%, or (C and D) 0.02%; full FLIM and intensity images in Fig. S18, scale bars = 10 µm.

We further decided to reinvestigate the effect of hydrophobic interfacers on the lipid-phase preference of probes. Changes in phase preferences are likely to influence mechanoresponsiveness of the probes, which is based on the ability to distinguish lipid phases. While our earlier attempt was hampered by poor delivery,^28^ maximized deliverability expected from dendrimers should allow us to elucidate the true impact of hydrophobic interfacing on flipper function. Since oleyl chains and cholesterol are natural components of *L*_d_ and *L*_o_ membranes, respectively, their attachment to flipper probes, as in G4O-Flipper 5 and G3C-Flipper 6, was expected to drive the probes toward respective phases. G3 dendrimer was selected for cholesteryl derivative 6 because of difficulties in characterizing G4 dendrimer products (see below). Flipper dendrimers without hydrophobic interfacers were not considered because, being overall much more hydrophilic and inverted-cone shaped amphiphiles than the original 1 with comparably poor membrane affinity,^28^ they were unlikely to partition well in membranes (Fig. 1e).

Flipper dendrimers 2–6 were prepared by target-oriented multistep synthesis, the best performing G4P-Flipper 4 was obtained in 26 steps (Schemes S1–S9). Size and amphiphilicity of 4 prevented recording of meaningful NMR spectra, as the peaks were always very broad, presumably due to micelle formation under all accessible conditions. Thus, as in peptide and protein chemistry, nature and purity of the final target were assessed by HPLC and high-resolution mass spectrometry (Fig. S21).

Added to *L*_d_ and *L*_o_ LUVs (large unilamellar vesicles), flipper dendrimers 1–6 produced the excitation spectra characteristic of less and more planarized flippers, respectively (Fig. 2a, b and Table S1).^15^ Independent of membrane order, the excitation maxima red-shifted with dendrimer size (+8 or +6 nm from 1 to 4), implying that the dendrimers improved the positioning of the probes within lipid bilayers. Fluorescence intensity was also enhanced by dendrimers, particularly in *L*_o_ LUVs, reflecting not only the better positioning but also the generally better partitioning of probes in the membranes (Fig. 2c, d, S3–S6, S9, S10, Tables S1 and S2). Even with favorable low probe/lipid ratio and vigorous stirring, the time needed to reach equilibrium in *L*_o_ membranes, for instance, decreased rapidly with increasing dendrimer generations (G2P 2: 1 h, G3P 3: 20 min, G4P 4: <5 min). Since the outer layer of the plasma membranes is highly ordered, these results promised better staining of cells by flipper dendrimers than by the original 1.^68^

Despite the higher apparent concentrations of flipper dendrimers 3 and 4 compared to G0 1 in membranes, dendrimers were less prone to photobleaching (Fig. S11). This indicated that the excited states of better-positioned and more planarized flippers undergo less intersystem crossing, thereby reducing singlet-oxygen generation.^23^ In PBS, the fluorescence intensity of G4P-Flipper 4 was about 20 times lower than in *L*_d_ LUVs but remained nearly constant for >30 min, whereas that of the G0 original 1 decreased to about half within the same period (Fig. 2e and S2). These results confirmed the better water solubility of the former, likely in a micellar form, which may lead to improved deliverability (Fig. 1e).

This assumption was verified by FLIM and confocal laser scanning microscopy (CLSM) of HeLa Kyoto (HK) cells labeled with flipper dendrimers 1–6 (Fig. 2, 3, S14–S19, and Table S4). At constant concentration and laser power (LP), labeling of the cells increased by generations from the weakest G2P 2 to the intermediate G3P 3, then to the best G4P 4, consistent with improved deliverability (Fig. 2f). The increased effective brightness of flippers with larger dendrimers was consistent with better deliverability as Israelachvili micelles, which was evinced by linear concentration dependence of fluorescence intensity (Fig. 2g and S15). Increased effective brightness allowed for correspondingly reduced laser power to acquire images at equal intensity. Reduced laser power translated into reduced phototoxicity^23^ and thus access to biological studies over longer periods of time. FLIM images of HK cells labeled with G0 1 and G4P 4 were recorded at LP = 0.2% (0.08 µW) and 0.02% (0.01 µW) to obtain comparable mean intensity counts per pixel for 1 (58) and 4 (43) in the plasma membranes. For the first scans under these conditions, similar images were obtained (Fig. 2i(A and C), top, S18). After 30 scans, cells labeled with the original G0 1 were blebbing (Fig. 2i(B), arrows), while G4P 4 reported identical intact cells (Fig. 2i(D)).

**Fig. 3:**
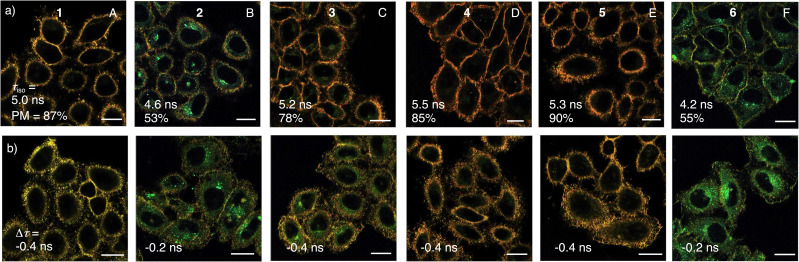
Sensitivity (*τ*_iso_), responsiveness (Δ*τ* = *τ*_hyper_ – *τ*_iso_), and plasma membrane selectivity (PM) of flipper dendrimers in (a) isoosmotic and (b) hyperosmotic HK cells. FLIM images of (a) isoosmotic and (b) hyperosmotic HK cells labeled with flippers 1 (A), 2 (B), 3 (C), 4 (D), 5 (E), and 6 (F, 0.3 µM, 20 min), with PM selectivity (counts in PM per total counts), lifetimes *τ* from phasor plots (Fig. S16) and lifetime changes Δ*τ* in response to osmotic shock (500 mM sucrose, *τ*_hyper_ in b minus *τ*_iso_ in a); scale bars = 20 µm, image intensities not comparable, lifetime color code as in Fig. 2.

Quantified as changes in cell area over scans (1 scan/2 min), effective phototoxicity was found significant with G0 1 but not with G4P 4 in HK cells at comparable fluorescence intensity using another FLIM setup (Fig. 2h and S19). Fluorescence lifetimes were constant during these experiments for G4P 4 at *τ* = 5.5 ns but slightly increased for G0 1 (Δ*τ* = 0.02 ns, Fig. S19). The increase in lifetimes under photoirradiation with G0 1 was previously attributed to lipid oxidation *via* photosensitized singlet oxygen generation.^23^ These results demonstrated that the increased fluorescence of G4P 4, due to apparently higher probe concentration in membranes and better positioning, enabled imaging at lower LP to minimize phototoxicity.

In FLIM images of isoosmotic HK cells, the selectivity of flipper probes is reported as the ratio of counts in the MOI, here the plasma membrane, divided by the total counts. The original Flipper-TR 1 recognized the MOI with 87% selectivity (Fig. 3a(A)). Because of increasing intracellular emission from less ordered environments, this selectivity dropped to 53% for G2P 2 (Fig. 3a(B)). From there, selectivity recovered with dendrimer size over 78% for G3P 3 and 85% for G4P 4 to 90% for G4O 5, surpassing even the original G0 1 (Fig. 3a(C–E)).

The mechanosensitivity of flippers is reported by the absolute fluorescence lifetime *τ* in the MOI. Increasing fluorescence lifetime originates mostly from flipper planarization in equilibrium in the ground state by mechanical compression from the environment.^15^ According to assumption-free and robust phasor analysis,^69–71^ the lifetime of palmitoylated flipper dendrimers increased from G0 1 with *τ* = 5.0 ns to 5.5 ns for G4P 4 (Fig. 3a, and S16). This increase was consistent with design expectations that palmitoyl interfacers drive more flippers into more ordered membrane microdomains and perhaps also contribute directly to planarization (Fig. 1e).

The responsiveness of flipper probes to changes in membrane tension is reported by the lifetime changes Δ*τ*. The original G0 1 reported decreasing membrane tension from hyperosmotic stress with Δ*τ* = −0.4 ns (Fig. 3A). For palmitoylated flipper dendrimers, this responsiveness to membrane tension was maintained, except for the poorly functional G2P 2 (Fig. 3B–D).

Replacement of the saturated palmitoyl in G4P 4 by an unsaturated oleyl interfacer in the fourth generation flipper dendrimer G4O 5 caused a slight increase in plasma membrane selectivity (90%) and a slight decrease in mechanosensitivity (*τ* = 5.3 ns) with unchanged responsiveness to tension (Δ*τ* = −0.4 ns, Fig. 3E). Cholesteryl interfacers in G3C 6 gave dysfunctional probes with poor effective brightness (Fig. 2f), selectivity (55%), mechanosensitivity (*τ* = 4.2 ns) and responsiveness (Δ*τ* = −0.2 ns, Fig. 3F). Overall, the effect of hydrophobic interfacers was minor for mechano-responsiveness but significant for deliverability.

The impact of dendrimers on the orientation and partitioning of flipper probes was examined by FLIM of GUVs (Fig. 4a–d, S12 and S13). Fluorescence lifetimes *τ* determined for all probes in *L*_d_ DOPC, *L*_o_ SM/CL and phase-separated DOPC/SM/CL GUVs were similar to each other (Fig. 4b). Record lifetimes found for large palmitoylated flipper dendrimers, particularly 4, in membranes of all orders were consistent with their record sensitivity in cells (Fig. 3a).

**Fig. 4:**
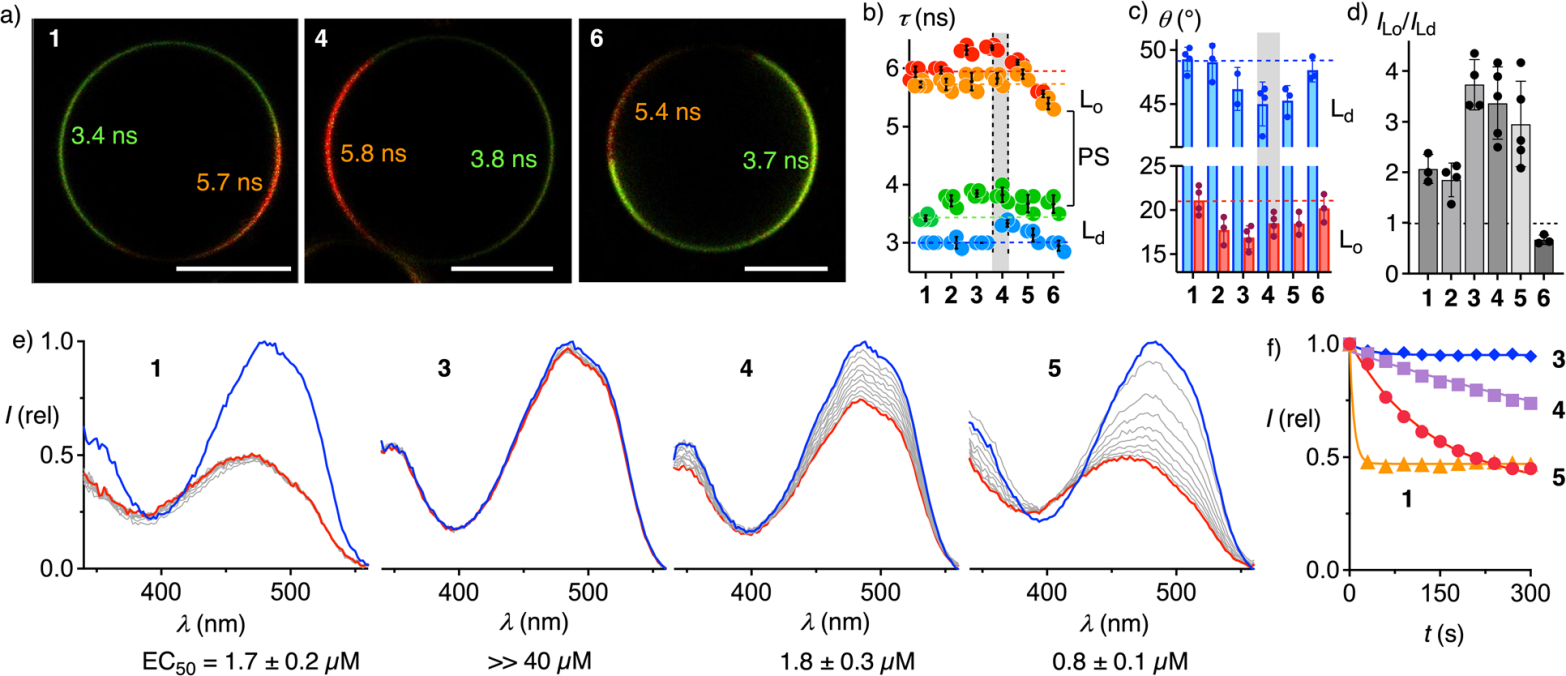
Interdomain (a–d) and intermembrane (e and f) distribution of flipper dendrimers. (a) Representative fast FLIM images of phase-separated (PS) *L*_d_ + *L*_o_ DOPC/SM/CL 58 : 25 : 17 GUVs with 1, 4, and 6 (∼1 µM) with fluorescence lifetimes (*τ*, ns, from phasor analysis) of each domain; scale bar = 10 µm, lifetime color code as in Fig. 2. (b) Fluorescence lifetimes of 1–6 in *L*_o_ SM/CL 7 : 3 (red), *L*_d_ DOPC (blue), or PS GUVs (orange and green). (c) Average angles of the transition dipoles relative to the membrane normal in *L*_d_ DOPC (blue) or *L*_o_ SM/CL GUVs (red). (d) Ratios of fluorescence intensities in *L*_o_ and *L*_d_ domains of PS GUVs. (e) Excitation spectra of 1,^28^3, 4, and 5 (*λ*_em_ = 600 nm) in *L*_o_ SM/CL 7 : 3 LUVs (blue) and *n* × 30 s after addition of *L*_d_ DOPC LUVs (decreasing intensities, grey to red: after 300 s), and EC_50_ values obtained using BSA instead of DOPC LUVs. (f) Time course of intensities at *λ*_ex_ = 488 nm in (e) for 1 (triangles), 3 (diamonds), 4 (squares) and 5 (circles).

Reporting on orientational variability or tilting disorder, the average tilt angle *θ* between the membrane normal and the transition dipole moment was estimated from the intensity ratio at the axial and equatorial regions of GUVs.^70,72^ Consistent with results of molecular mechanics simulations,^73^*θ* ∼50° found for 1 in *L*_d_ GUVs indicated high tilting disorder (Fig. 4c, blue), while *θ* ∼20° in *L*_o_ GUVs supported more uniform, nearly parallel orientation of the probe relative to membrane normal (Fig. 4c, red). Increasing dendrimer size in 3–5 decreased flipper tilting disorder particularly in disordered membranes. Contrary to dendron size, tilting disorder was independent of the saturation of the alkyl tail (4*vs.*5), but increased by cholesteryl interfacers (3*vs.*6).

According to the intensities in FLIM images of phase-separated GUVs, the 3.4 : 1 selectivity of G4P 4 for *L*_o_ domains exceeded the 2.1 : 1 selectivity of G0 1 clearly (Fig. 4a and d). These differences supported that palmitoyl interfacers drive flippers into ordered domains and that this displacement accounts for high fluorescence lifetime in cells. Longer lifetimes in all phases suggested that the proximal palmitoyl organizers contribute to orienting and planarizing flipper probes. G3C 6 selectively labeled *L*_d_ domains, although cholesterol as such accounts for the emergence of *L*_o_ domains (Fig. 4a and d). Indicating that the confined hydrophobic environment of *L*_o_ membrane cannot accommodate the rigid and bulky flipper-cholesterol conjugate 6, this result explained the poor performance in cells (Fig. 3F).

The addition of unlabeled *L*_d_ LUVs to *L*_o_ LUVs labeled with the original G0 1 caused an immediate drop of fluorescence, corresponding to rapid intermembrane probe transfer from ordered to disordered membranes through aqueous medium with a *t*_50_ <30 s (Fig. 4e and f).^28^ Palmitoylation in G2P 2 ^29^ and G3P-Flipper 3 fully inhibited this intermembrane transfer. Further increase of the hydrophilic dendrimer in G4P 4 partially restored intermembrane transfer up to an estimated *t*_50_ ≈ 15 min (Fig. 4e and f). The disorganizing oleyl interfacer in G4O homolog 5 further accelerated intermembrane transfer to a *t*_50_ = 3.4 min. Extraction of the probe from *L*_o_ LUVs with BSA gave similar trends from facile for 1, 4 and 5 (EC_50_ = 0.8–1.8 µM) to barely possible for 2 and 3 (EC_50_ >>40 µM, Fig. S7 and S8). These results were consistent with the partition coefficients of probes in *L*_o_ LUVs (Table S2). While reversible partitioning and intermembrane transfer coincided well with internalization-free plasma membrane labeling (Fig. 3) with leaflet-level precision^74^ and are of interest for superresolution microscopy (1, 4, 5),^75^ irreversible partitioning provides solutions to unique challenges such as the elucidation of liposomal delivery (2, 3).^76^

In summary, besides being beautiful and synthetically ambitious, giant flipper dendrimers are found to address the main weakness of the original flipper probes, that is, the phototoxicity. Improved, Israelachvili-inspired delivery provides access to strongly fluorescent cells, allowing measurements at lower laser power for longer times without phototoxicity. Flipper dendrimers do not only show high effective brightness and thus low phototoxicity, they also excel with selectivity, mechanosensitivity, and responsiveness. They are the result of conceptually understood mechanophore interfacing rather than mechanophore engineering and demonstrate that synthetic efforts are meaningful if the problem addressed is significant. The main lesson learned is that to solve persistent practical problems, it can be worthwhile to focus not on the problematic object itself but on its integration into the environment, which calls for translational supramolecular chemistry. The usefulness of the original flipper in the community suggests that flipper dendrimers with high effective brightness will enable wonderful research worldwide, also with regard to multicellular systems with 2-photon FLIM, where laser power is the main source of toxicity through heating.

## Experimental section

See ESI.

## Author contributions

N. G.-S. and F. B. synthesized and N. G.-S., J. M. G.-A., T. M. and N. S. characterized the probes, A. R., N. S. and S. M. directed the study, all authors contributed to the design of experiments, data interpretation and manuscript writing.

## Conflicts of interest

The authors declare the following competing financial interest: The University of Geneva has licensed four Flipper-TR® probes to Spirochrome for commercialization.

## Supplementary Material

SC-OLF-D5SC09248E-s001

## Data Availability

Data for this paper are available at Zenodo at https://doi.org/10.5281/zenodo.18454876 (the link will be activated before the paper is published). Supplementary information (SI): detailed procedures and results for all reported experiments. See DOI: https://doi.org/10.1039/d5sc09248e.
