# Supplementary material for: Flipper dendrimers
Source: Chem Sci. 2026 Feb 20;17(15):7509–15. doi: 10.1039/d5sc09248e (PMC12922593; doi:10.1039/d5sc09248e)
Supplement: SC-017-D5SC09248E-s001 [file SC-017-D5SC09248E-s001.pdf]

Supplementary Information (SI) for Chemical Science.
This journal is © The Royal Society of Chemistry 2026

Electronic supplementary information

Flipper dendrimers

Nerea Gonzalez-Sanchis,[a,b] Felix Bayard,[a,b] Juan Manuel García-Arcos,[d] Tithi Mandal,[c] Aurelien Roux,[c] Naomi Sakai,[a,b] and Stefan Matile*[a,b]

[a]Department of Organic Chemistry, University of Geneva

Geneva, Switzerland

[b]National Centre of Competence in Research (NCCR) Molecular Systems Engineering

Basel, Switzerland

[c]Department of Biochemistry, University of Geneva

Geneva, Switzerland

[d]Swiss Institute for Experimental Cancer Research (ISREC), School of Life Sciences, Swiss Federal Institute of Technology Lausanne (EPFL), Lausanne, Switzerland

*stefan.matile@unige.ch

Table of contents

1.	Materials and methods	S3
2.	Synthesis	S5
	2.1. Synthesis of dendrimers (G2, G3, G4)	S6
	2.2. Synthesis of Palmitoyl and Oleyl Interfacers (P, O)	S10
	2.3. Synthesis of cholesteryl interfacers (C)	S13
	2.4. Synthesis of G3P conjugates	S16
	2.5. Synthesis of G3C conjugates	S18
	2.6. Synthesis of G4P and G4O conjugates	S20
	2.7. Synthesis of G3P-Flippers	S23
	2.8. Synthesis of G3C-Flippers	S25
	2.9. Synthesis of G4P- and G4O-Flippers	S27
3.	Fluorescence spectroscopy in solution	S29
4.	Fluorescence spectroscopy in LUVs	S29
	4.1. Extraction with BSA	S32
	4.2. Intermembrane exchange	S34
	4.3. Partition coefficients	S34
	4.4. Photobleaching kinetics	S36
5.	Fluorescence lifetime imaging microscopy of GUVs	S37
6.	Fluorescence lifetime imaging microscopy of cells	S40
	6.1. General procedure of FLIM imaging in HeLa Kyoto cells	S40
	6.2. Kinetics	S40
	6.3. Concentration dependence	S41
	6.4. Comparison of probes	S42
	6.5. Phototoxicity evaluation	S44
7.	Supplementary references	S47

8. RP-HPLC chromatograms S48

9. NMR spectra S49

1. Materials and methods

As in reference S1. The reagents used for synthesis were purchased from Fluka, Sigma-Aldrich and TCI. 1,2-dioleoyl-*sn*-glycero-3-phosphocholine (DOPC), egg sphingomyelin (SM) and mini extruder were purchased from Avanti Polar Lipids. Dulbecco's phosphate-buffered saline (PBS, pH = 7.4), Fluorobrite^{TM} DMEM, Penicillin-Streptomycin, Fetal Bovine Serum, TrypLE Express Enzyme were obtained from Thermo Fischer Scientific. Analytical thin layer chromatography (TLC) was performed on silica gel 60 F254 (Merck, 0.2 mm). Column chromatography was carried out on silica gel 60 (SilicaFlash® P60, SILICYCLE, 230-400 mesh). Reverse-phase (RP) flash chromatography was performed on a Biotage Isolera^{TM} Spektra using pre-packed Scorpius C18 5.4 g. Fluorescence measurements were performed on a FluoroMax-4 spectrofluorometer (Horiba Scientific), equipped with a stirrer and a temperature controller. Fluorescence spectra were background subtracted and corrected with factors supplied by the manufacturer. Melting points (Mp) were recorded on a Melting Point M-565 (BUCHI). Alpha-D values were measured on a Polarimeter P-1030 (Jasco). IR spectra were recorded on a Perkin Elmer Spectrum Two FT-IR spectrometer (ATR, Golden Gate) and are reported as wavenumbers ν in cm^{-1} and the peaks are marked as s (strong), m (medium), w (weak), br (broad). All ^{1}H and ^{13}C NMR spectra were recorded on a Bruker 400 MHz or 500 MHz spectrometer at room temperature (25 °C) and are reported as chemical shifts (δ) in ppm relative to TMS (δ = 0 ppm). Spin multiplicities are reported as s (singlet), d (duplet), t (triplet), m (multiplet) and b (broad) with coupling constants J given in Hz. ^{1}H and ^{13}C resonances were reported with the aid of additional information from 1D and 2D NMR (H,H-COSY, DEPT 135, HSQC, HMBC). LC-MS analyses were performed on Advion Avant® UHPLC system equipped with a Thermo C18 Hypersil GOLD column (50 × 2.1 mm, 1.9 µm particles size) with Advion Expression® CMS in ESI mode. ESI-HRMS was measured on Xevo G2-S Tof (Waters). MALDI

MS analyses were performed with α-cyano-4-hydroxycinnamic acid as a matrix using a Bruker MALDI Autoflex Speed TOF/TOF. All mass data are reported as mass-per-charge ratio *m/z* ([assignment]).

Abbreviations. CL: Cholesterol; Cy: Cyclohexane; DIPEA: Diisopropylethylamine; DMEM: Dulbecco's modified Eagle medium; DMF: Dimethylformamide; DMSO: Dimethyl sulfoxide; EC_{50}: Half maximal effective concentration; EtOAc: Ethyl acetate; FA: Formic acid; FDMEM: FluoroBrite^{TM} DMEM; FLIM: Fluorescence lifetime imaging microscopy; GUV: Giant unilamellar vesicle; HATU: Hexafluorophosphate azabenzotriazole tetramethyl uronium; HBTU: 3-[Bis(dimethylamino)methyliumyl]-3*H*-benzotriazol-1-oxide hexafluorophosphate; HK: HeLa Kyoto, LUV: Large unilamellar vesicle; MALDI: Matrix-assisted laser desorption/ionization; PBS: Dulbecco's phosphate-buffered saline; RP: Reverse phase; rt: Room temperature; TFA: Trifluoroacetic acid; THPTA: Tris(3-hydroxypropyltriazolylmethyl)amine.

2. Synthesis

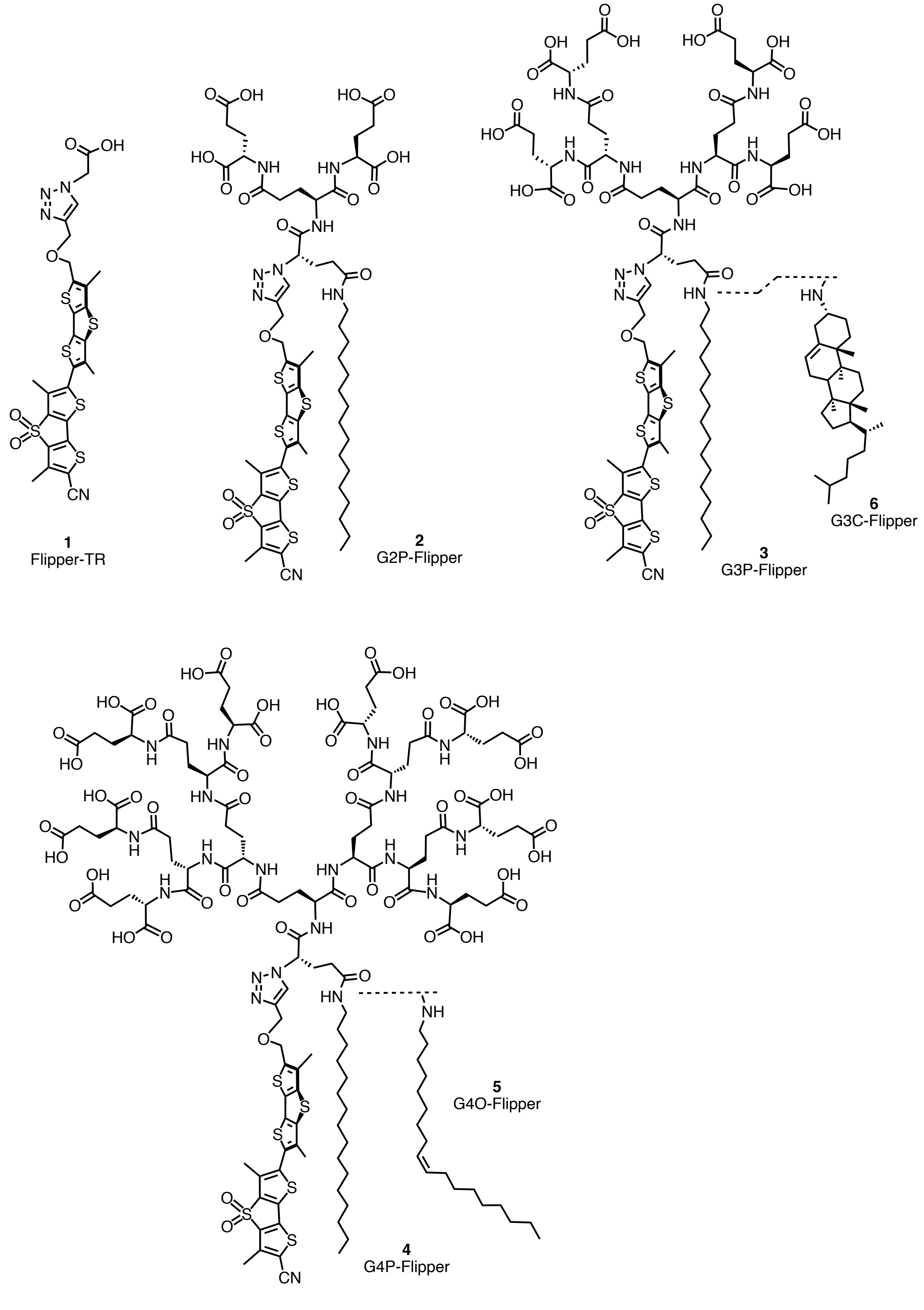

Fig. S1 Structure of the flipper probes made and used in this study.

2.1. Synthesis of dendrimers (G2, G3, G4)

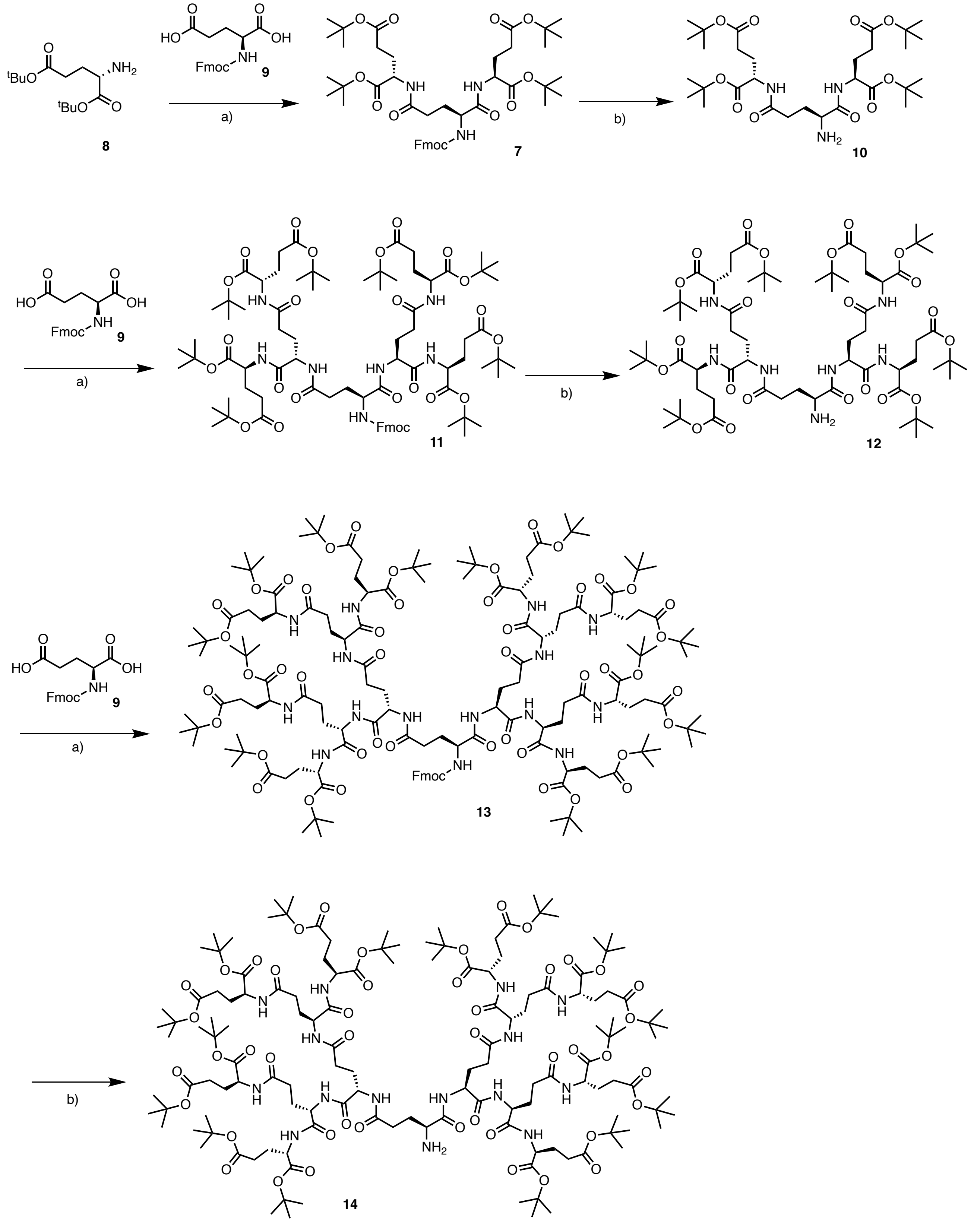

Scheme S1 Synthesis of dendrimers G2 **10**, G3 **12** and G4 **14**. a) HBTU, DIPEA, DMF, rt, 5 h, 53-77%. b) Me_2NH, THF, rt, 45 min, 70-95%.

Compound 7 was prepared as described in S2 from **8** and **9**.

Compound 10 was prepared as described in S2.

Compound 11 was synthesized following the procedure for **7**. To a solution of **9** (300 mg, 0.812 mmol), HBTU (585 mg, 1.54 mmol), and DIPEA (115 μL, 0.652 mmol) in dry DMF (4 mL) under N_2 atmosphere at rt, **10** (1.00 g, 1.62 mmol) was added, and the mixture was stirred at rt for 5 h. The solvent was evaporated *in vacuo*, EtOAc (6 mL) was added to the solution, and the organic phase was washed with aqueous citric acid (20%, 1 x 5 mL), sat. $NaHCO_3$ (1 x 5 mL), and LiCl (5%, 2 x 10 mL), dried over Na_2SO_4, and filtered. The solvent was evaporated under reduced pressure. The crude product was purified by column chromatography (SiO_2, CH_2Cl_2/pentane 6:4) to afford **11** (500 mg, 77%) as a colorless oil. Spectroscopic data were comparable to those reported in the literature.[S3] R_f (CH_2Cl_2/pentane 6:4): 0.3; $[\alpha]_D^{20}$ -7.8 (*c* 1.0, $CHCl_3$); IR (neat): 3286 (br, N–H), 3071 (w, Ar–C–H), 2977 (m, C–H), 2932 (m, C–H), 1727 (s, C=O carbamate), 1640 (s, amide C=O), 1537 (m, N–H), 1449 (m, C–H), 1392 (m, C–H), 1366 (m, C–H, t-Bu), 1277 (m, C–O), 1251 (m, C–O), 1184 (m, C–O), 1042 (w, C–N), 962 (w, Ar–H), 847 (w, Ar–H), 789 (w, Ar–H), 757 (w, Ar–H); ^{1}H NMR (400 MHz, $CDCl_3$): 8.74 (d, $^3J_{H\text{-}H}$ = 6.6 Hz, 1H), 8.37 – 8.30 (m, 3H), 7.93 (d, $^3J_{H\text{-}H}$ = 9.0 Hz, 1H), 7.79 (d, $^3J_{H\text{-}H}$ = 9.2 Hz, 1H), 7.78 – 7.72 (m, 2H), 7.60 – 7.58 (m, 2H), 7.40 – 7.35 (m, 3H), 7.30 – 7.28 (m, 2H), 5.17 (d, $^3J_{H\text{-}H}$ = 8.6 Hz, 1H), 4.79 – 4.72 (m, 2H), 4.62 – 4.54 (m, 2H), 4.40 (dd, $^3J_{H\text{-}H}$ = 8.9, $^3J_{H\text{-}H}$ = 5.7 Hz, 1H), 4.25 – 4.11 (m, 4H), 3.87 (d, $^3J_{H\text{-}H}$ = 8.1 Hz, 1H), 2.49 – 2.17 (m, 23H), 1.88 – 1.81 (m, 6H), 1.47 – 1.43 (m, 72H); ^{13}C NMR (101 MHz, $CDCl_3$): 173.8 (2 x C), 173.6 (2 x C), 173.5 (2 x C), 172.4 (2 x C), 172.2 (2 x C), 171.7 (2 x C), 171.6 (2 x C), 154.8 (C), 143.7 (2 x C), 141.0 (2 x C), 127.4 (2 x CH), 126.8 (2 x CH), 125.3 (2 x CH), 119.6 (2 x CH), 83.1 (C), 82.9 (C), 82.8 (C), 82.7 (C), 80.5 (2 x C), 80.3 (C), 79.7 (C), 66.8 (CH_2), 53.2 (CH), 52.9 (CH), 52.3 (CH), 52.2 (CH), 52.2 (2 x CH), 52.0 (CH), 46.9 (CH), 32.0 (CH_2), 31.8 (2 x CH_2), 31.3 (3 x CH_2), 28.0 (6 x CH_3), 27.9 (6 x CH_3), 27.9 (6 x CH_3), 27.8 (6 x CH_3), 27.1 (CH_2), 26.6 (CH_2); MS (ESI): 1594 ($[M+H]^+$).

Compound 12 was synthesized following the procedure for **10**. Compound **11** (90 mg, 0.056 mmol) was dissolved in a solution of Me_2NH in THF (2 M, 0.90 mL, 1.8 mmol), and the mixture was

stirred at rt for 45 min. The solvent was evaporated, and the crude product was purified by column chromatography (SiO_2, CH_2Cl_2/acetone 1:1) to afford **12** (74 mg, 95%) as a colorless oil. R_f (CH_2Cl_2/acetone 1:1): 0.2; $[\alpha]_D^{20}$ -4.5 (*c* 1.0, $CHCl_3$); IR (neat): 3295 (br, N–H), 2979 (m, C–H), 2932 (m, C–H), 1725 (s, C=O, ester), 1639 (s, C=O, amide), 1535 (m, N–H), 1450 (m, C–H), 1366 (m, C–H), 1249 (m, C–O), 1147 (m, C–O), 1035 (w, C–N), 846 (w, C–H), 743 (w, C–H); ^{1}H NMR (400 MHz, $CDCl_3$): 8.48 (d, $^3J_{H-H}$ =7.0 Hz, 1H), 8.33 (d, $^3J_{H-H}$ =9.1 Hz, 1H), 8.28 (m, 2H), 7.87 (d, $^3J_{H-H}$ = 8.9 Hz, 1H), 7.83 (d, $^3J_{H-H}$ = 9.0 Hz, 1H), 4.70 – 4.67 (m, 2H), 4.57 – 4.53 (m, 2H), 4.24 – 4.20 (m, 1H), 4.12 – 4.06 (m, 1H), 3.01 (dd, $^3J_{H-H}$ = 11.4, 4.0 Hz, 1H), 2.41 – 2.23 (m, 14H), 2.16 – 2.00 (m, 10H), 1.93 – 1.80 (m, 5H), 1.46 – 1.42 (m, 72H); ^{13}C NMR (101 MHz, $CDCl_3$): 173.9 (C), 173.7 (C), 173.5 (C), 173.3 (C), 173.2 (C), 172.9 (C), 172.8 (C), 172.4 (C), 172.3 (C), 171.8 (C), 171.8 (C), 171.7 (C), 171.6 (2 x C), 83.1 (C), 82.9 (C), 82.8 (C), 82.8 (C), 80.5 (C), 80.5 (C), 80.4 (C), 80.3 (C), 53.2 (CH), 53.0 (CH), 52.6 (CH), 52.2 (CH), 52.1 (CH), 52.1 (2 x CH), 31.9 (CH_2), 31.8 (CH_2), 31.7 (CH_2), 31.4 (CH_2), 31.3 (CH_2), 29.5 (CH_2), 29.2 (CH_2), 29.8 (CH_2), 27.9 (4 x CH_3), 27.9 (4 x CH_3), 27.9 (6 x CH_3), 27.8 (5 x CH_3), 27.8 (5 x CH_3), 27.3 (CH_2), 27.3 (CH_2), 27.1 (CH_2), 27.1 (CH_2), 26.7 (CH_2), 26.6 (CH_2); MS (ESI): 1372 ($[M+H]^+$).

Compound 13 was synthesized following the procedure for **7**. To a solution of **9** (40.4 mg, 0.110 mmol), HBTU (83.0 mg, 0.219 mmol), and DIPEA (45 μL, 0.25 mmol) in dry DMF (1 mL) under N_2 atmosphere, after 10 min, **12** (315 mg, 0.230 mmol) was added, and the mixture was stirred at rt for 6 h. The solvent was evaporated *in vacuo*, EtOAc (3 mL) was added to the solution, and the organic phase was washed with aqueous citric acid (20%, 1 x 3 mL), sat. $NaHCO_3$ (1 x 5 mL), and LiCl (5%, 2 x 5 mL), and dried over Na_2SO_4, and filtered. The solvent was evaporated under reduced pressure. The crude product was purified by column chromatography (SiO_2, CH_2Cl_2/MeOH 95:5) to afford **13** (177 mg, 53%) as a colorless oil. R_f (CH_2Cl_2/MeOH 95:5): 0.2; IR (neat): 3353 (br, N–H), 3234 (br, N–H), 2979 (m, C–H), 2931 (m, C–H), 2285 (w), 2161 (w), 1979 (w), 1718 (s, C=O, carbamate), 1654 (s, C=O, amide), 1628 (m, C=C, aromatic), 1559 (m, N–H), 1516 (m, N–H), 1454 (m, C–H), 1391 (m, C–H), 1366 (m, C–H, t-Bu), 1299 (m, C–O), 1244 (m, C–O), 1147 (s, C–O),

1027 (w, C–N), 966 (w, Ar–H), 842 (w, Ar–H), 786 (w, Ar–H), 762 (w, Ar–H), 749 (w, Ar–H), 722 (w, Ar–H); ^{1}H NMR (400 MHz, CD_3OD): 7.75 (d, $^{3}J_{H-H}$ = 7.6 Hz, 2H), 7.67 (d, $^{3}J_{H-H}$ = 7.5 Hz, 2H), 7.35 (t, $^{3}J_{H-H}$ = 7.4 Hz, 2H), 7.30 – 7.22 (m, 2H), 4.54 – 4.44 (m, 2H), 4.41 – 4.19 (m, 13H), 4.14 (t, $^{3}J_{H-H}$ = 7.2 Hz, 1H), 4.08 – 4.06 (m, 1H), 3.85 – 3.81 (m, 1H), 2.45 – 2.27 (m, 26H), 2.22 – 2.17 (m, 4H), 2.15 – 2.02 (m, 17H), 1.96 – 1.77 (m, 13H), 1.53 – 1.37 (m, 144H); ^{13}C NMR (101 MHz, CD_3OD): 174.9 (C), 174.8 (2 x C), 174.8 (3 x C), 174.8 (2 x C), 174.6 (C), 174.5 (C), 174.3 (C),174.1 (C), 174.1 (C), 174.0 (C), 173.6 (2 x C), 173.6 (2 x C), 173.5 (C), 173.4 (C), 173.4 (3 x C), 173.4 (C), 173.3 (C), 173.2 (C), 173.1 (C), 172.8 (C), 172.7 (C), 172.4 (C), 157.9 (C), 145.3 (C), 145.2 (C), 142.5 (2 x C), 128.8 (C), 128.8 (C), 128.3 (C), 128.2 (C), 126.6 (C), 126.5 (C), 120.9 (2 x C), 83.5 (C), 83.5 (C), 83.5 (C), 83.4 (C), 83.4 (C), 83.3 (C), 83.2 (C), 82.9 (C), 81.8 (C), 81.8 (C), 81.7 (C), 81.7 (C), 81.7 (C), 81.6 (C), 68.4 (CH_2), 54.8 (CH), 54.2 (CH), 54.1 (CH), 53.9 (CH), 53.8 (CH), 53.8 (2 x CH), 53.8 (2 x CH), 53.7 (2 x CH), 53.7 (2 x CH), 53.7 (CH), 53.4 (CH), 47.8 (CH), 33.1 (CH_2), 33.0 (CH_2), 32.8 (CH_2), 32.7 (CH_2), 32.6 (2 x CH_2), 32.6 (CH_2), 32.5 (2 x CH_2), 32.5 (2 x CH_2), 32.5 (2 x CH_2), 32.4 (2 x CH_2), 29.8 (CH_2), 29.6 (CH_2), 29.3 (CH_2), 29.1 (CH_2), 28.5 (8 x CH_3), 28.4 (9 x CH_3), 28.4 (9 x CH_3), 28.4 (8 x CH_3), 28.4 (8 x CH_3), 28.3 (6 x CH_3), 28.2 (CH_2), 28.0 (2 x CH_2), 28.0 (3 x CH_2), 28.0 (3 x CH_2), 27.9 (CH_2), 27.8 (CH_2); MALDI-MS: 3094 ($[M+Na]^+$).

Compound 14 was synthesized following the procedure for compound **10**. Compound **13** (84 mg, 0.030 mmol) was dissolved in a solution of Me_2NH in THF (2 M, 0.40 mL, 6.9 mmol), and the mixture was stirred at rt for 45 min. The solvent was evaporated and the crude product was purified by column chromatography (SiO_2, CH_2Cl_2/MeOH 95:5) to afford **14** (55 mg, 70%) as a colorless oil. R_f (CH_2Cl_2/MeOH 95:5): 0.25; $[\alpha]_D^{20}$ -9.0 (*c* 1.0, $CHCl_3$); IR (neat): 3363 (br, N–H), 2975 (m, C–H), 2931 (m, C–H), 2864 (m, C–H), 1716 (s, C=O, ester), 1660 (s, C=O, amide), 1531 (m, N–H), 1512 (m, N–H), 1486 (m, C–H), 1449 (m, C–H), 1367 (m, C–H, t-Bu), 1328 (m, C–O/C–N), 1308 (m, C–O), 1256 (m, C–O), 1229 (m, C–O), 1149 (s, C–O), 1054 (m, C–N), 1006 (m, C–O), 937 (w), 848 (w), 767 (w), 754 (w);^{1}H NMR (400 MHz, CD_3OD): 4.49 – 4.33 (m, 12H), 4.21 – 4.17 (m, 1H), 4.15 – 4.11 (m, 1H), 3.19 (dd, $^{3}J_{H-H}$ = 8.9, 5.6 Hz, 1H), 2.44 – 2.29 (m, 30H), 2.15 – 2.04 (m, 15H), 2.00

– 1.96 (m, 5H), 2.94 – 1.84 (m, 10H), 1.49 – 1.48 (m, 72H), 1.45 – 1.45 (m, 72H); ^{13}C NMR (101 MHz, CD_3OD): 174.9 (C), 174.9 (2 x C), 174.9 (3 x C), 174.8 (5 x C), 174.7 (3 x C), 174.5 (C), 173.6 (C), 173.6 (C), 173.5 (C), 173.5 (C), 173.4 (C), 173.4 (C), 173.3 (C), 173.3 (C), 173.3 (2 x C), 173.1 (C), 173.0 (2 x C), 172.8 (C), 172.6 (C), 83.5 (C), 83.5 (C), 83.5 (C), 83.5 (C), 83.4 (C), 83.4 (C), 83.3 (C), 83.2 (C), 81.9 (2 x C), 81.8 (2 x C), 81.8 (2 x C), 81.7 (C), 81.6 (C), 54.8 (CH), 54.6 (CH), 54.2 (CH), 54.2 (CH), 54.1(CH), 54.0 (CH), 54.0 (CH), 53.9 (2 x CH), 53.9 (2 x CH), 53.8 (2 x CH), 53.8 (CH), 53.7 (CH), 33.0, 32.9 (CH_2), 32.8 (CH_2), 32.7 (CH_2), 32.7 (3 x CH_2), 32.6 (4 x CH_2), 32.5 (2 x CH_2), 32.5 (2 x CH_2), 29.4 (CH_2), 29.1 (CH_2), 28.9 (CH_2), 28.8 (2 x CH_2), 28.5 (8 x CH_3), 28.5 (4 x CH_3), 28.4 (8 x CH_3), 28.4 (10 x CH_3), 28.4 (10 x CH_3), 28.4 (8 x CH_3), 28.2 (CH_2), 28.1 (CH_2), 28.0 (2 x CH_2), 28.0 (3 x CH_2), 27.9 (2 x CH_2), 27.8 (CH_2); MS (ESI): 1426 ([M+2H]$^{2+}$).

2.2. Synthesis of Palmitoyl and Oleyl Interfacers (P, O)

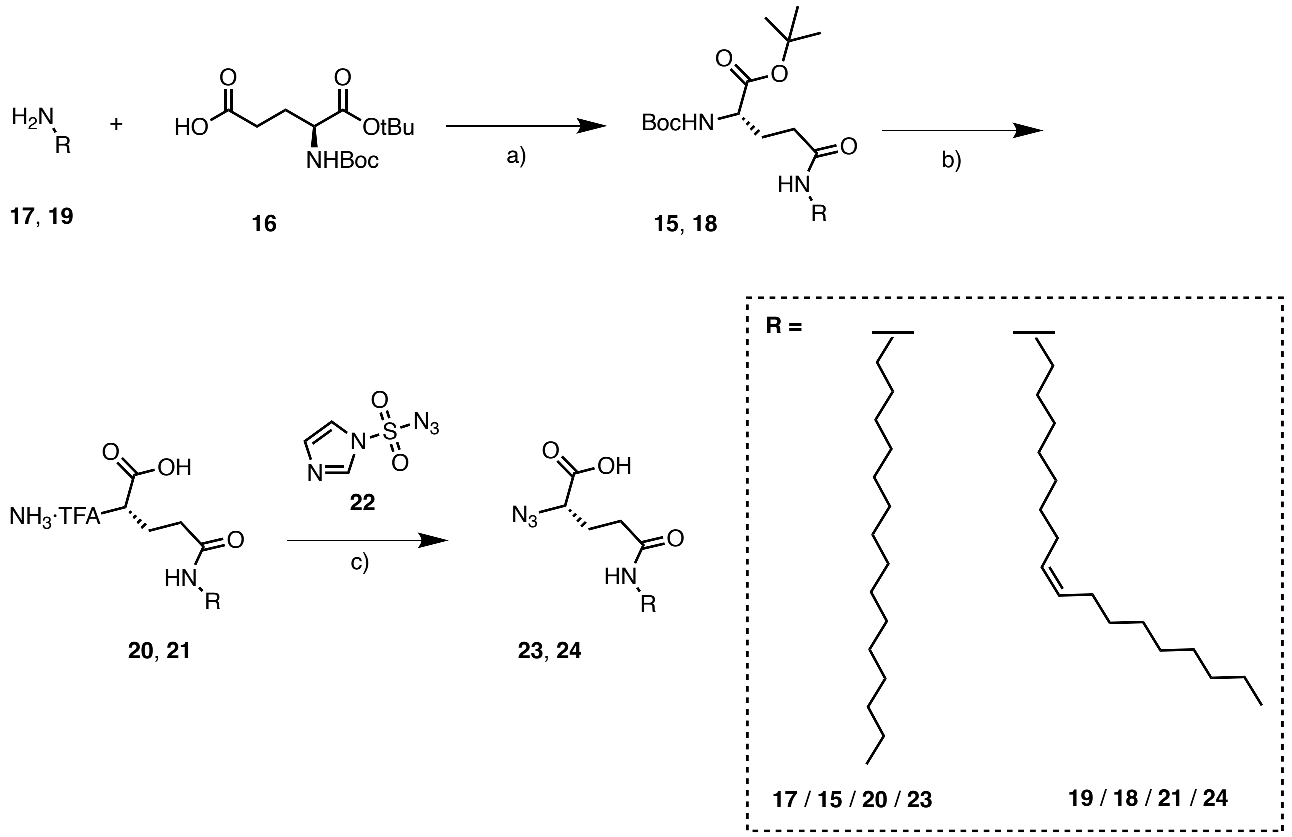

Scheme S2 Synthesis of P and O interfacers **23** and **24**. a) HBTU, DIPEA, DMF, rt, 30 min, 87%. b) TFA, CH_2Cl_2, rt, 1 h, 90%. c) **22**, K_2CO_3, $CuSO_4$ 5 H_2O, DMF, rt, 16 h, 36%.

Compound 15 was synthesized by adapting the reported procedure in the reference S2. To a solution of **16** (1.00 g, 3.30 mmol) in dry DMF (48 mL) under N_2 atmosphere, DIPEA (723 µL, 4.15 mmol) and HBTU (1.25 g, 3.30 mmol) were added. Then, **17** (947 mg, 3.92 mmol) was added, and the mixture was stirred at rt for 30 min. The solvent was reduced *in vacuo* and 1 M aq. HCl (20 mL) was added. The aqueous phase was extracted with EtOAc (2 × 20 mL). The combined organic fractions were washed with water (1 × 30 mL) and brine (3× 20 mL), dried over Na_2SO_4, and finally the solvent was evaporated under reduced pressure. The crude product was purified by column chromatography (SiO_2, Cy/EtOAc 2:3) to afford **15** (1.51 g, 87%) as a colorless solid. R_f (Cy/EtOAc 2:3): 0.3; Mp: 69-70 °C; $[\alpha]_D^{20}$ +2.3 (*c* 1.0, $CHCl_3$); IR (neat): 3354 (br, N–H), 2918 (m, C–H), 2849 (m, C–H), 1732 (s, C=O, ester), 1688 (m, C=O, amide), 1645 (s, C=O, amide), 1511 (m, N–H), 1469 (m, C–H), 1444 (m, C–H), 1369 (m, C–H, t-Bu), 1247 (m, C–O), 1221 (m, C–O), 1153 (m, C–O), 1057 (w, C–N), 1031 (w, C–N), 950 (w), 908 (w), 850 (w), 776 (w), 720 (w), 653 (w); ^{1}H NMR (400 MHz, $CDCl_3$): 6.20 (s, 1H), 5.23 (d, $^3J_{H\text{-}H}$ = 8.3 Hz, 1H), 4.17 – 4.11 (m, 1H), 3.26 – 3.21 (m, 2H), 2.26 – 2.22 (m, 2H), 2.19 – 2.11 (m, 1H), 1.89 – 1.80 (m, 1H), 1.46 – 1.44 (m, 18H), 1.31 – 1.25 (m, 27H), 0.89 – 0.86 (m, 3H); ^{13}C NMR (101 MHz, $CDCl_3$): 172.1 (C), 171.6 (C), 156.1 (C), 82.5 (C), 80.1 (C), 53.6 (CH), 39.8 (CH), 33.1 (CH_2), 32.1 (CH_2), 30.0 (CH_2), 29.9 (3 x CH_2), 29.8 (2 x CH_2), 29.8 (CH_2), 29.8 (CH_2), 29.7 (CH_2), 29.7 (CH_2), 29.5 (CH_2), 29.5 (CH_2), 28.5 (3 x CH_3), 28.1 (3 x CH_3), 27.1 (CH_2), 22.8 (CH_2), 14.3 (CH_3).

Compound 18 was synthesized from **19** according to reported procedures.[S4]

Compound 20 was synthesized by adapting the reported procedure in the reference S4. To a solution of **15** (700 mg, 1.33 mmol) in CH_2Cl_2 (7.43 mL), TFA (7.4 mL, 97 mmol) was added. The reaction was stirred for 1 h at rt. The solvent was removed *in vacuo,* and the product was used without further purification, affording **20** (580 mg, 90%) as a colorless solid. R_f (CH_2Cl_2/acetone 1:1): 0.3; Mp: 85-86 °C; $[\alpha]_D^{20}$ +2.6 (*c* 1.0, $CHCl_3$); IR (neat): 3400 (br, N–H), 3243 (br, N–H), 2919 (m, C–H), 2851 (m, C–H), 2525 (w), 1764 (w, C=O, ester), 1725 (s, C=O, ester), 1644 (s, C=O, amide), 1557 (m, amide II, N–H), 1508 (m, N–H), 1471 (m, C–H), 1447 (m, C–H), 1376 (m, C–H), 1310 (m,

C–O/C–N), 1242 (m, C–O), 1177 (m, C–O), 1163 (m, C–O), 1146 (m, C–O), 986 (w), 871 (w), 841 (w), 801 (w), 779 (w), 722 (w); ^{1}H NMR (400 MHz, CD_3OD): 4.01 (dd, $^{3}J_{H-H}$ = 6.7, 5.9 Hz, 1H), 3.17 (td, $^{3}J_{H-H}$ = 7.0, 1.0 Hz, 2H), 2.47 (t, $^{3}J_{H-H}$ = 6.9 Hz, 2H), 2.23 – 2.09 (m, 2H), 1.52 – 1.48 (m, 2H), 1.29 (s, 26H), 0.92 – 0.88 (m, 3H); ^{13}C NMR (101 MHz, CD_3OD + a drop DMSO-d_6): 173.8 (C), 171.6 (C), 53.5 (CH), 40.5 (CH_2), 33.0 (CH_2), 32.5 (CH_2), 30.7 (4 x CH_2), 30.7 (4 x CH_2), 30.4 (3 x CH_2), 28.0 (CH_2), 27.2 (CH_2), 23.7 (CH_2), 14.6 (CH_3).

Compound 21 was prepared as described in S4.

Compound 22 was prepared as described in S5.

Compound 23 was synthesized by adapting the procedure reported in S5. To a solution of **20** (280 mg, 0.56 mmol) in DMF (6.2 mL), anhydr. K_2CO_3 (197 mg, 1.43 mmol), $CuSO_4$ 5·H_2O (3.6 mg, 14 μmol), and **22** (160 mg, 0.93 mmol) were added. The reaction was stirred at rt for 16 h, and the mixture was quenched with 1 M aq. HCl (10 mL). The aqueous phase was extracted with EtOAc (1 × 20 mL). The combined organic phases were washed with water (30 mL) and brine (4 × 30 mL), dried over Na_2SO_4, filtered, and finally the solvent was evaporated under reduced pressure. The crude product was purified by column chromatography (SiO_2, CH_2Cl_2/MeOH 95:5) to afford **23** (80 mg, 36%) as a brown solid. R_f (CH_2Cl_2:MeOH 95:5): 0.15; IR (neat): 3400 (br, N–H), 3000–2500 (br, O–H, COOH), 2920 (m, C–H), 2850 (m, C–H), 2105 (s, N_3), 1712 (s, C=O, carboxylic acid), 1650 (s, C=O, amide), 1552 (m,), 1468 (m, C–H), 1446 (m, C–H), 1372 (m, C–H), 1298 (m, C–O), 1240 (m, C–O), 1175 (m), 720 (w); ^{1}H NMR (500 MHz, $CDCl_3$): 5.93 (t, $^{3}J_{H-H}$ = 5.7 Hz, 1H), 4.06 (s, 1H), 3.26 – 3.22 (m, 2H), 2.36 (t, $^{3}J_{H-H}$ = 7.5 Hz, 2H), 2.21 – 2.17 (m, 1H), 2.10 – 2.06 (m, 1H), 1.51 – 1.48 (m, 2H), 1.31 – 1.25 (m, 26H), 0.88 (t, $^{3}J_{H-H}$ = 6.9 Hz, 3H); ^{13}C NMR (126 MHz, $CDCl_3$): 172.6 (C), 172.3 (C), 61.1 (CH), 39.9 (CH_2), 32.0 (CH_2), 31.8 (CH_2), 29.6 (3 x CH_2), 29.6 (CH_2), 29.6 (CH_2), 29.5 (CH_2), 29.4 (CH_2), 29.3 (CH_2), 29.2 (CH_2), 29.2 (CH_2), 27.5 (CH_2), 26.8 (CH_2), 22.6 (CH_2), 14.0 (CH_3).

Compound 24 was prepared as described in S4.

2.3. Synthesis of cholesteryl interfacers (C)

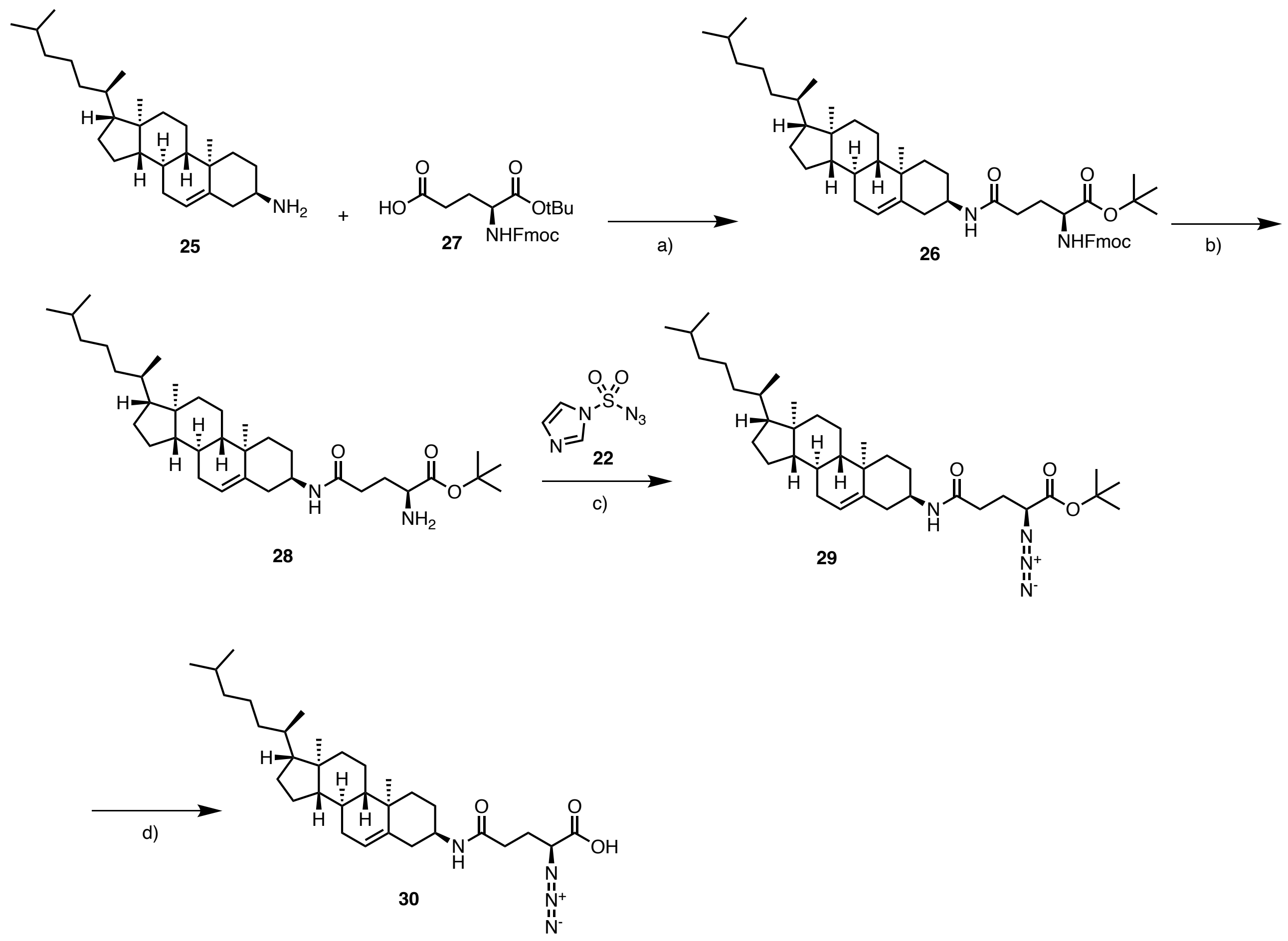

Scheme S3 Synthesis of C interfacers **30**. a) **27**, HBTU, DIPEA, DMF, rt, 4 h, 95%. b) Me_2NH, THF, rt, 45 min, 93%. c) **22**, K_2CO_3, $CuSO_4$ $5H_2O$, DMF, rt, 16 h, 72%. d) TFA, CH_2Cl_2, rt, 3 h, 67%.

Compound 25 was synthesized according to previously published procedures.[S6]

Compound 26 was synthesized by adapting the reported procedure in the reference S4. To a solution of Fmoc-Glu-OtBu **27** (600 mg, 1.14 mmol) in dry DMF (40 mL) under N_2 atmosphere, DIPEA (500 µL 1.4 mmol) and HBTU (540 mg, 1.42 mmol) were added. Then, **25** (544 mg, 1.41 mmol) was added and the mixture was stirred at rt for 4 h. The solvent was reduced *in vacuo* and 1 M aq. HCl (50 mL) was added. The aqueous phase was extracted with EtOAc (2 × 50 mL). The combined organic fractions were washed with water (2 × 50 mL) and brine (4 × 50 mL), dried over

Na_2SO_4 and finally the solvent was evaporated under reduced pressure. The crude product was purified by column chromatography (SiO_2, Cy/EtOAc 2:1) to afford **26** (1.04 g, 95%) as a colorless solid. R_f (Cy/EtOAc 2:1) 0.15; Mp: 68-70 °C; $[\alpha]_D^{20}$ -4.3 (*c* 1.0, $CHCl_3$); IR (neat): 3161 (br, N-H), 2943 (w, C-H) 1731 (m, C=O), 1642 (s, C=C), 1542 (m, C-H), 1447 (w, C-H), 1220 (w, C-O); ^1H NMR (400 MHz, $CDCl_3$): 7.78 – 7.75 (m, 2H), 7.60 (d, $^3J_{H-H}$ = 7.5 Hz, 2H), 7.45 – 7.36 (m, 2H), 7.36 – 7.27 (m, 2H), 6.28 (d, $^3J_{H-H}$ = 7.9 Hz, 1H), 5.62 (d, $^3J_{H-H}$ = 8.1 Hz, 1H), 5.42 – 5.33 (m, 1H), 4.53 – 4.42 (m, 1H), 4.34 – 4.23 (m, 3H), 4.18 (m, 1H), 2.56 (d, $^2J_{H-H}$ = 14.3 Hz, 1H), 2.30 – 2.16 (m, 3H), 2.02 – 1.60 (m, 8H), 1.48 (s, 9H), 1.55 – 1.06 (m, 12H), 1.00 (s, 3H), 0.94 – 0.78 (m, 10H), 0.62 (s, 3H); ^{13}C NMR (101 MHz, $CDCl_3$): 171.4 (C), 171.3 (C), 156.7 (C), 144.2 (C), 143.7 (C), 141.5 (C), 141.4 (C), 139.2 (C), 127.9 (CH), 127.9 (CH), 127.2 (CH), 125.4 (CH), 125.2 (CH), 123.6 (CH), 120.2 (CH), 120.2 (CH), 82.7 (C), 77.4 (CH), 67.4 (CH_2), 56.8 (CH), 56.2 (CH), 54.0 (CH), 50.4 (CH), 47.4 (CH), 46.1 (CH), 42.3 (C), 39.7 (CH_2), 37.5 (C), 37.3 (CH_2), 36.3 (CH_2), 35.9 (CH), 34.1 (CH_2), 33.1 (CH_2), 32.1 (CH_2), 31.9 (CH), 30.1 (CH_2), 28.3 (CH_2), 28.2 (CH_3), 28.2 (CH_3), 26.2 (CH_2), 24.3 (CH_2), 24.1 (CH_2), 23.0 (CH_3), 22.7 (CH), 20.9 (CH_2), 19.1 (CH_3), 18.8 (CH_3), 11.9 (CH_3); MS (ESI): 816 ($[M+Na]^+$).

Compound 28. Compound **26** (1.00 g, 1.26 mmol) was dissolved in a solution of Me_2NH in THF (2 M, 6.3 mL, 13 mmol), and the mixture was stirred at rt for 45 min. The solvent was evaporated, and the crude product was purified by column chromatography (SiO_2, CH_2Cl_2/MeOH 96:4) to afford **28** (669 mg, 93%) as a colorless solid. R_f (CH_2Cl_2/MeOH 96:4): 0.03; Mp: 129-130 °C; $[\alpha]_D^{20}$ -56 (*c* 1.0, $CHCl_3$); IR (neat): 3286 (w, N-H), 3168 (w, C-H), 2932 (m, C-H), 2866 (w, C-H), 1727 (m, C=O), 1630 (s, C=C), 1433 (w, C-H), 1367 (m, C-H), 1252 (w, C-N), 1152 (m, C-O), 730 (w, C=C); ^1H NMR (400 MHz, $CDCl_3$): 6.03 (d, $^3J_{H-H}$ = 7.9 Hz, 1H), 5.37 – 5.35 (m, 1H), 4.18 – 4.10 (m, 1H), 3.34 (dd, $^3J_{H-H}$ = 9.0, 4.7 Hz, 1H), 2.60 – 2.52 (m, 1H), 2.39 – 2.21 (m, 2H), 2.14 – 2.05 (m, 1H), 2.05 – 1.90 (m, 3H), 1.89 – 1.78 (m, 1H), 1.77 – 1.63 (m, 4H), 1.65 – 1.50 (m, 10H), 1.46 (s, 9H), 1.40 – 1.05 (m, 8H), 1.02 (s, 4H), 1.04 – 0.94 (m, 2H), 0.92 (d, $^3J_{H-H}$ = 6.5 Hz, 3H), 0.88 (d, $^3J_{H-H}$ = 1.8 Hz, 3H), 0.86 (d, $^3J_{H-H}$ = 1.8 Hz, 3H), 0.68 (s, 3H); ^{13}C NMR (101 MHz, $CDCl_3$): 175.3 (C), 171.6 (C),

139.3 (C), 123.6 (CH), 81.4 (C), 77.4 (CH), 57.0 (CH), 56.3 (CH), 54.4 (CH), 50.8 (CH), 45.8 (CH), 42.5 (C), 39.9 (CH_2), 39.7 (CH_2), 37.5 (C), 37.3 (CH_2), 36.3 (CH_2), 36.0 (CH), 34.4 (CH_2), 33.4 (CH_2), 32.1 (CH), 32.0 (CH), 30.5 (CH_2), 28.4 (CH_2), 28.2 (CH_3), 28.2 (CH_3), 26.3 (CH_2), 24.4 (CH_2), 24.0 (CH_2), 23.0 (CH_3), 22.7 (CH_3), 20.9 (CH_2), 19.1 (CH_3), 18.9 (CH_3), 12.0 (CH_3); MS (ESI): 594 ($[M+Na]^+$).

Compound 29 was synthesized by adapting reported procedure.[S5] To a solution of **28** (200 mg, 350 µmol) in DMF (3.7 mL), anhydr. K_2CO_3 (72.6 mg, 525 µmol), $CuSO_4$ $5H_2O$ (1.4 mg, 5.6 µmol) and **22** (88.1 mg, 420 µmol) were added. The reaction was stirred at rt for 16 h and the mixture was quenched with 1 M aq. HCl (40 mL). The aqueous phase was extracted with EtOAc (1 × 50 mL). The combined organic phases were washed with water (30 mL) and brine (4 × 30 mL), dried over Na_2SO_4, filtered and finally the solvent was evaporated under reduced pressure. The crude product was purified by column chromatography (SiO_2, Cy/EtOAc 3:1) to afford **29** (150 mg, 72%) as a colorless solid. R_f (Cy/EtOAc 3:1): 0.27; $[\alpha]_D^{20}$ -29.5 (*c* 1.0, $CHCl_3$); IR (neat): 3305 (w, N-H), 2931 (m, C-H), 2902 (m, C-H), 2867 (w, C-H), 2106 (m, N_3), 1738 (m, C=O), 1634 (s, C=C), 1367 (m, C-H), 1221 (m, C-N), 1148 (s ,C-O), 843 (w, C=C); ^1H NMR (400 MHz, $CDCl_3$): 5.57 (d, $^3J_{H-H}$ = 7.7 Hz, 1H), 5.40 – 5.34 (m, 1H), 4.13 (dt, $^3J_{H-H}$ = 7.7, 3.4 Hz, 1H), 3.83 (dd, $^3J_{H-H}$ = 9.2, 4.7 Hz, 1H), 2.61 – 2.55 (m, 1H), 2.29 – 2.27 (m, 2H), 2.22 – 2.13 (m, 1H), 2.06 – 1.88 (m, 4H), 1.88 – 1.78 (m, 1H), 1.78 – 1.52 (m, 7H), 1.50 (s, 9H), 1.46 – 1.04 (m, 15H), 1.02 (s, 5H), 0.92 (d, $^3J_{H-H}$ = 6.5 Hz, 3H), 0.87 (d, $^3J_{H-H}$ = 1.8 Hz, 3H), 0.85 (d, $^3J_{H-H}$ = 1.8 Hz, 3H), 0.68 (s, 3H); ^{13}C NMR (101 MHz, $CDCl_3$): 170.4 (C), 169.4 (C), 139.0 (C), 123.9 (CH), 83.2 (C), 61.9 (CH), 56.9 (CH), 56.3 (CH), 50.6 (CH), 45.9 (CH), 42.5 (C), 39.9 (CH_2), 39.7 (CH_2), 37.5 (C), 37.2 (CH_2), 36.3 (CH_2), 36.0, 34.3 (CH_2), 32.6 (CH_2), 32.1 (CH_2), 32.0, 28.4 (CH_2), 28.2 (CH_3), 27.3 (CH_2), 26.2 (CH_2), 24.4 (CH_2), 24.0 (CH_2), 23.0 (CH_3), 22.7 (CH_3), 20.9 (CH_2), 19.0 (CH_3), 18.9 (CH_3), 12.0 (CH_3); MS (ESI): 619 ($[M+Na]^+$).

Compound 30 was synthesized by adapting the reported procedure in reference S4. To a solution of **29** (62.6 mg, 105 µmol) in CH_2Cl_2 (1.33 mL), triethylsilane (26.5 µL, 166 µmol) and TFA (400 µL, 5.2 mmol) were added. The reaction was stirred for 3 h at rt. The solvent was removed *in*

vacuo and the crude product was purified by column chromatography (SiO_2, CH_2Cl_2/MeOH/AcOH 96:4:2) to afford **30** (38.0 mg, 67%) as a colorless solid. The spectra were in accordance with previously published results.[S4]

2.4. Synthesis of G3P conjugates

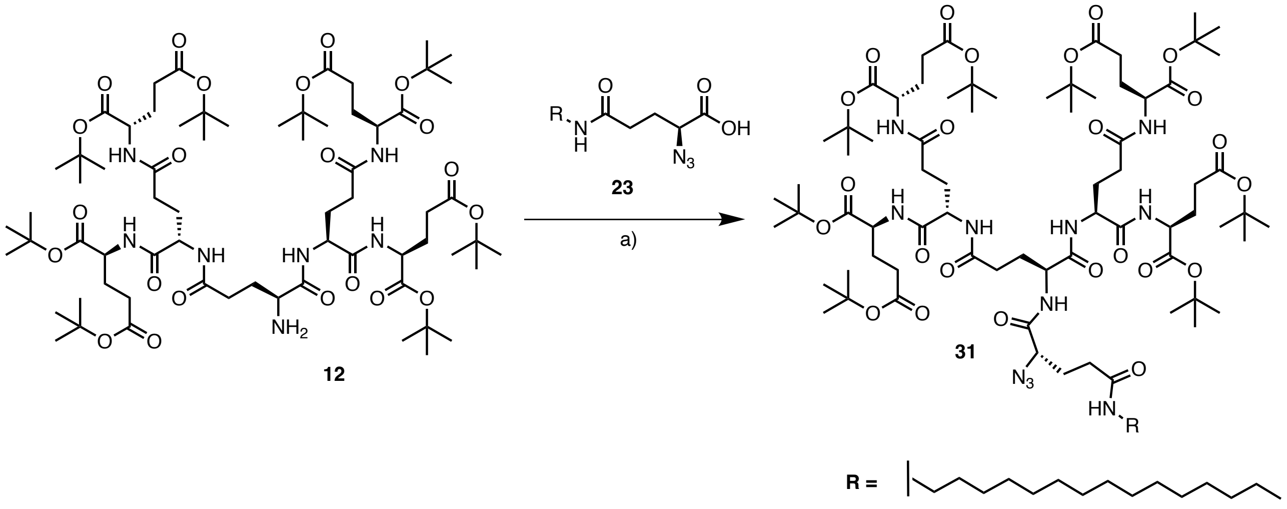

Scheme S4 Synthesis of G3P conjugates **31**. a) **23**, HBTU, DIPEA, DMF, rt, 2 h, 56%.

Compound 31 was synthesized by adapting the reported procedure in the reference S2. To a solution **23** (23.6 mg, 0.0595 mmol) in dry DMF (305 µL) under N_2 atmosphere, DIPEA (10 µL, 0.06 mmol) and HBTU (22.0 mg, 0.0580 mmol) were added. Then, **12** (87.0 mg, 0.0635 mmol) was added, and the mixture was stirred at rt for 2 h. The crude product was dissolved in 5 mL of EtOAc, washed with water (1 × 10 mL) and brine (3× 10 mL), dried over Na_2SO_4, and finally the solvent was evaporated under reduced pressure. The crude product was purified by column chromatography (SiO_2, CH_2Cl_2/MeOH, 95:5) to afford **31** (62 mg, 56%) as a brown solid. R_f (CH_2Cl_2/MeOH, 95:5): 0.3; IR (neat): 3274 (br, N–H), 2924 (m, C–H), 2102 (s, N_3), 1728 (s, C=O, ester), 1647 (s, C=O amide), 1635 (s, C=O amide), 1545 (m,), 1456 (m, C–H), 1391 (m, C–H, t-Bu), 1366 (m, C–H, t-Bu), 1297 (m, C–O), 1245 (m, C–O), 1149 (s, C–O), 1066 (w, C–N), 1027 (w), 972 (w), 847 (w), 787 (w), 751 (w); ^{1}H NMR (400 MHz, $CDCl_3$): 8.77 (d, $^3J_{H\text{-}H}$ = 6.9 Hz, 1H), 8.41 (d, $^3J_{H\text{-}H}$ = 8.7 Hz, 1H), 8.30 (t, $^3J_{H\text{-}H}$ = 8.5 Hz, 2H), 7.91 (s, 1H), 7.71 (d, $^3J_{H\text{-}H}$ = 8.8 Hz, 1H), 6.71 (d, $^3J_{H\text{-}H}$ = 8.0 Hz, 1H), 6.67 (d,

$^3J_{H-H}$ = 5.7 Hz, 1H), 4.79 – 4.75 (m, 1H), 4.68 – 4.62 (m, 1H), 4.56 – 4.50 (m, 2H), 4.22 – 4.19 (m, 1H), 4.15 – 4.11 (m 1H), 4.02 – 3.95 (m, 2H), 3.25 – 3.11 (m, 2H), 2.52 – 2.06 (m, 26H), 1.89 – 1.81 (m, 6H), 1.46 – 1.39 (m, 72H), 1.26 – 1.23 (m, 28H), 0.86 (t, $^3J_{H-H}$ = 6.7 Hz, 3H); ^{13}C NMR (101 MHz, $CDCl_3$): 173.7 (C), 173.6 (C), 173.4 (C), 173.4 (C), 173.2 (C), 172.6 (C), 172.5 (C), 172.4 (C), 172.4 (2 x C), 171.6 (C), 171.6 (C), 171.6 (C), 171.5 (C), 170.8 (C), 167.8 (C), 83.0 (C), 82.9 (C), 82.8 (C), 82.7 (C), 80.5 (C), 80.5 (C), 80.4 (C), 80.1 (C), 62.2 (CH), 53.1 (CH), 52.6 (CH), 52.2 (CH), 52.1 (CH), 52.1 (CH), 50.7 (CH), 50.6 (CH), 39.4 (CH_2), 31.9 (3 x CH_2), 31.9 (3 x CH_2), 31.7 (2 x CH_2), 31.7 (2 x CH_2), 31.7 (CH_2), 31.6 (CH_2), 31.3 (CH_2), 31.2 (CH_2), 31.2 (CH_2), 29.5 (5 x CH_2), 29.5 (3 x CH_2), 29.4 (2 x CH_2), 29.4 (CH_2), 29.2 (CH_2), 29.2 (CH_2), 27.9 (4 x CH_3), 27.9 (7 x CH_3), 27.8 (4 xCH_3), 27.8 (4 x CH_3), 27.8 (4 x CH_3), 27.7 (CH_3), 27.3 (2 x CH_3), 27.3 (2 x CH_3), 27.0 (2 x CH_3), 26.8 (CH_3), 26.7 (CH_3), 26.5 (CH_3), 22.5 (CH_2), 13.9 (CH_3).

2.5. Synthesis of G3C conjugates

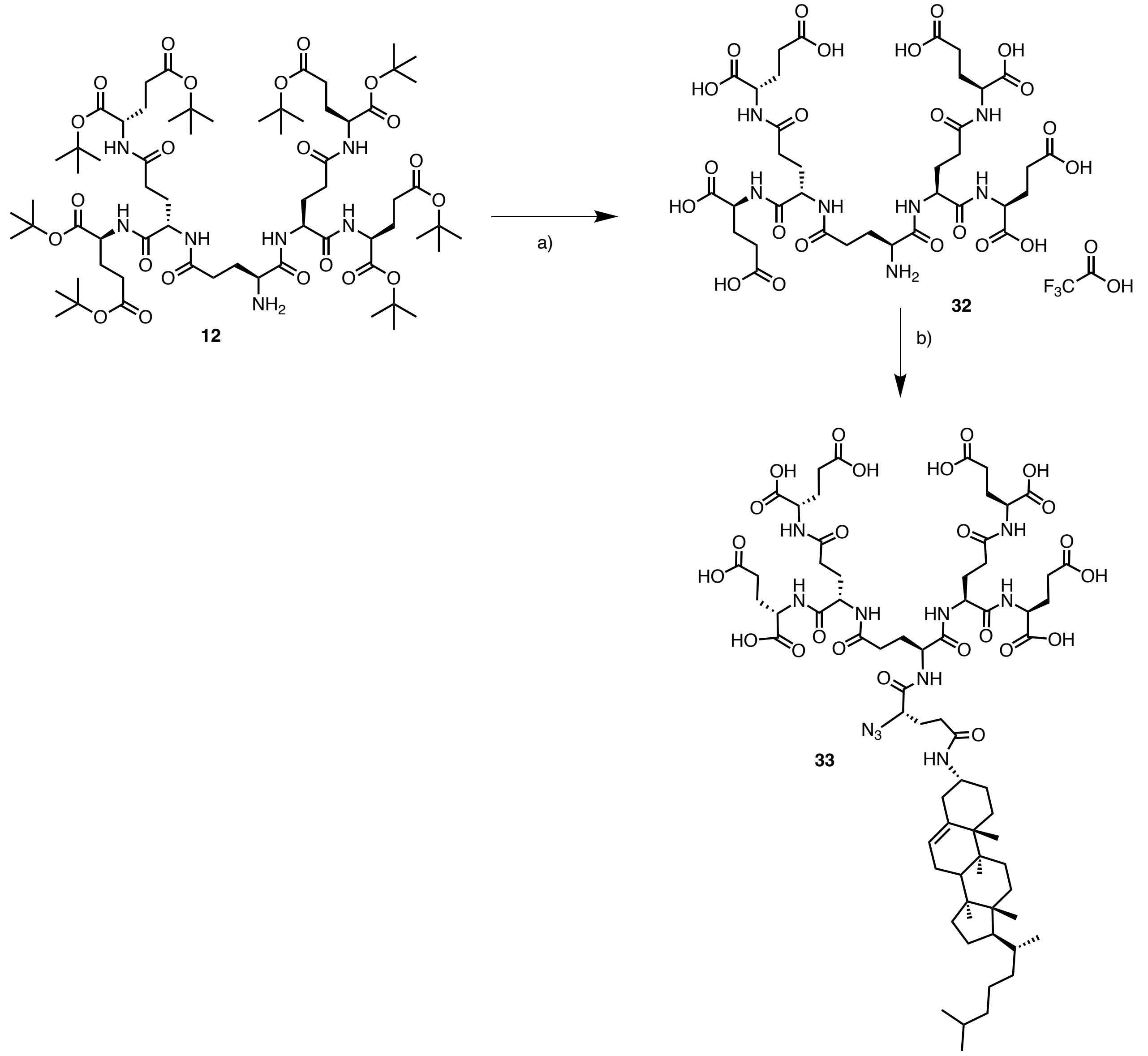

Scheme S5 Synthesis of G3C conjugates **33**. a) TFA, CH_2Cl_2, rt, 20 h, 81%. b) **30**, HATU, DIPEA, DMF, rt, 30 min, 40%.

Compound 32 was synthesized by adapting the reported procedure in reference S2. To a solution of **12** (52.0 mg, 37.9 µmol) in CH_2Cl_2 (290 µL), TFA (290 µL, 3.8 mmol) was added. The reaction was stirred for 20 h at rt. The solvent was removed *in vacuo* and the crude product was purified by RP-flash chromatography (Scorpius C18, 5.4 g, gradient elution, CH_3CN/H_2O + 0.1% TFA 0:1 to 1:0) to afford **32** (31.7 mg, 81%) as a colorless solid. IR (neat): 3330 (br, w, O-H), 3062 (w, C-H), 2941 (w, C-H), 1713 (s, C=O) 1634 (s, C=O), 1415 (m, C-H), 1184 (s, C-O), 1140 (m, C-

N), 797 (w, C-H); ^{1}H NMR (500 MHz, D_2O): 4.53 – 4.46 (m, 2H), 4.46 – 4.38 (m, 3H), 4.29 (dd, $^3J_{H-H}$ = 8.8, 6.0 Hz, 1H), 3.95 (dd, $^3J_{H-H}$ = 8.3, 5.6 Hz, 1H), 2.58 – 2.32 (m, 14H), 2.29 – 2.16 (m, 5H), 2.16 – 2.06 (m, 3H), 2.05 – 1.94 (m, 6H); ^{13}C NMR (126 MHz, D_2O): 177.0 (C), 177.0 (C), 176.9 (C), 176.8 (C), 175.2 (C), 175.1 (C), 174.9 (C), 174.9 (C), 174.7 (C), 174.6 (C), 174.1 (C), 173.8 (C), 173.0 (C), 169.0 (C), 53.2 (CH), 53.1 (CH), 52.0 (CH), 52.0 (CH), 52.0 (CH), 31.3 (CH_2), 31.2 (CH_2), 30.2 (CH_2), 30.0 (CH_2), 30.0 (CH_2), 29.8 (CH_2), 29.8 (CH_2), 26.9 (CH_2), 26.8 (CH_2), 26.6 (CH_2), 25.7 (CH_2), 25.7 (CH_2), 25.6 (CH_2); MS (ESI): 923 ([M+H]$^+$).

Compound 33 was synthesized by adapting the reported procedure in reference S2. **30** (7.6 mg, 14 µmol), HATU (5.4 mg, 14 µmol), and DIPEA (3.9 µL, 22 µmol) were dissolved in dry DMF (0.1 mL) under N_2, and the mixture was stirred for 15 min at rt. This solution was added to a solution of **32** (10.3 mg, 11.2 µmol) and DIPEA (19.5 µL, 112 µmol) in dry DMF (0.2 mL) and stirred for 30 min at rt. The volatiles were removed under reduced pressure, and the residue was suspended in water. The resulting precipitate was filtered off, and the filtrate was lyophilized. The remaining solid was triturated with Et_2O/CH_2Cl_2 1:1 (3 × 2 mL) and acetone (3 × 2 mL) to give **33** (6.4 mg, 40%) as a colorless solid. The product was used in the next step without further purification. IR (neat): 3301 (br, w, O-H), 2932 (m, C-H), 2868 (w, C-H), 2108 (w, N_3), 1718 (m, C=O), 1635 (s, C=O, C=C), 1534 (s, C-H), 1450 (w, C-H), 1382 (w, C-H), 1206 (m, C-N), 1172 (m, C-O), 798 (w, C=C); ^{1}H NMR (500 MHz, DMSO-d_6): 8.40 – 8.32 (m, 1H), 8.25 – 8.17 (m, 3H), 8.09 – 8.01 (m, 3H), 7.37 (d, $^3J_{H-H}$ = 7.4 Hz, 1H), 5.24 (s, 1H), 4.29 – 4.13 (m, 4H), 3.95 (s, 1H), 3.80 (dt, $^3J_{H-H}$ = 8.7, 5.4 Hz, 1H), 2.45 – 2.39 (m, 1H), 2.33 – 2.13 (m, 17H), 2.04 – 1.84 (m, 5H), 1.84 – 1.65 (m, 7H), 1.61 – 1.46 (m, 4H), 1.44 – 1.28 (m, 2H), 1.29 – 1.20 (m, 2H), 1.17 – 0.97 (m, 3H), 0.96 (s, 3H), 0.90 (d, $^3J_{H-H}$ = 6.4 Hz, 3H), 0.85 (d, $^3J_{H-H}$ = 2.5 Hz, 3H), 0.84 (d, $^3J_{H-H}$ = 2.5 Hz, 3H), 0.65 (s, 3H); ^{13}C NMR (126 MHz, DMSO-d_6): 173.7 (C), 173.7 (C), 173.6 (C), 173.3 (C), 171.8 (C), 171.5 (C), 170.9 (C), 170.6 (C), 170.5 (C), 169.3 (C), 138.6 (C), 121.9 (CH), 61.2 (CH), 61.0 (CH), 56.4 (CH), 55.6 (CH), 52.2 (CH), 51.9 (CH), 51.2 (CH), 51.2 (CH), 51.1 (CH), 49.2 (CH), 45.1 (CH), 41.9 (C), 36.6 (C), 36.3 (CH_2), 35.7 (CH_2), 35.2 (CH), 33.0 (CH_2), 31.7 (CH_2), 31.6 (CH_2), 31.5 (CH_2), 31.4 (CH), 30.1 (CH_2), 30.0

(CH_2), 30.0 (CH_2), 28.2 (CH_2), 27.8 (CH_2), 27.4 (CH), 26.3 (CH_2), 25.7 (CH_2), 23.9 (CH_2), 23.3 (CH_2), 22.7 (CH_3), 22.5 (CH_2), 22.4 (CH_3), 20.3 (CH_2), 18.7 (CH_3), 18.6 (CH_3), 11.7 (CH_3); MS (ESI): 1446 ($[M+H]^+$).

2.6. Synthesis of G4P and G4O conjugates

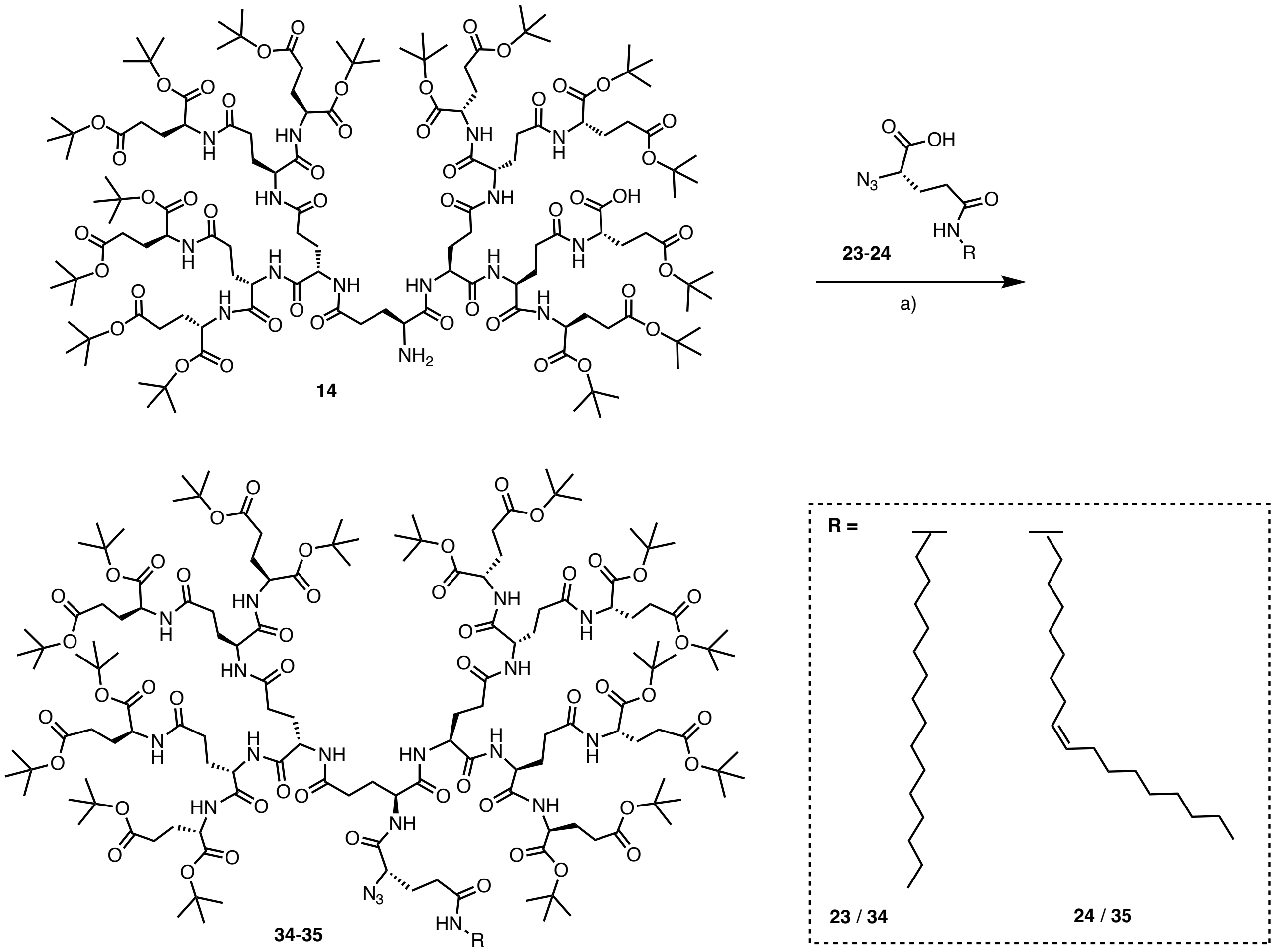

Scheme S6 Synthesis of G4P and G4O conjugates **34** and **35**. a) **23/24**, HBTU, DIPEA, DMF, rt, 3-4 h, 55-88%.

Compound 34 was synthesized by adapting the reported procedure in the reference S2. To a solution of **23** (13.2 mg, 0.0334 mmol) in dry DMF (0.7 mL) under N_2 atmosphere, DIPEA (30 μL, 0.2 mmol) and HBTU (11.7 mg, 0.0309 mmol) were added. After 5 min, **14** (80.0 mg, 0.0280 mmol) was added, and the mixture was stirred at rt for 3 h. The crude product was dissolved in 5 mL of

EtOAc, washed with sat. $NaHCO_3$ (1 × 10 mL), citric acid (20%, 1× 10 mL) and LiCl (5%, 2× 10 mL), dried over Na_2SO_4, and finally the solvent was evaporated under reduced pressure. The crude product was purified by column chromatography (SiO_2, CH_2Cl_2) to afford **34** (80 mg, 88%) as a colorless solid. R_f (CH_2Cl_2): 0.3; IR (neat): 3285 (br, N–H), 2977 (m, C–H), 2928 (m, C–H), 2106 (s, N_3), 1728 (s, C=O, ester), 1635 (s, C=O, amide), 1537 (m,), 1450 (m, C–H), 1392 (m, C–H, t-Bu), 1367 (m, C–H, t-Bu), 1251 (m, C–O), 1149 (s, C–O), 1037 (w, C–N), 961 (w), 847 (w), 753 (w); ^1H NMR (400 MHz, CD_3OD): 4.56 – 4.53 (m, 2H), 4.48 – 4.31 (m, 10H), 4.21 (t, $^3J_{H-H}$ = 8.1 Hz, 1H), 4.13 – 4.00 (m, 2H), 4.01 (dd, $^3J_{H-H}$ = 7.4, 5.9 Hz, 1H), 3.24 – 3.14 (m, 2H), 2.44 – 2.34 (m, 31H), 2.18 – 2.06 (m, 24H), 1.91 – 1.89 (m, 9H), 1.51 – 1.46 (m, 144H), 1.31 (s, 28H), 0.94 – 0.90 (m, 3H); ^{13}C NMR (101 MHz, CD_3OD): 174.9 (4 x C), 174.8 (3 x C), 174.6 (2 x C), 174.5 (2 x C), 174.2 (3 x C), 173.6 (2 x C), 173.5 (2 x C), 173.4 (4 x C), 173.3 (2 x C), 173.1 (2 x C), 173.0 (C), 172.9 (C), 172.8 (C), 172.5 (C), 171.3 (C), 83.6 (2 x C), 83.5 (3 x C), 83.4 (C), 83.2 (C), 83.1 (C), 81.9 (2 x C), 81.8 (3 x C), 81.7 (C), 81.7 (C), 81.6 (C), 63.5 (C), 54.2 (2 x CH), 53.9 (5 x CH), 53.8 (6 x CH), 53.6 (3 x CH), 40.6 (CH_2), 33.1 (2 x CH_2), 32.8 (4 x CH_2), 32.7 (7 x CH_2), 32.7 (2 x CH_2), 32.5 (1 x CH_2), 30.8 (9 x CH_2), 30.8 (4 x CH_2), 30.5 (48 x CH_2) 28.5 (16 x CH_3), 28.5 (16 x CH_3), 28.4 (16 x CH_3), 28.1 (4 x CH_2), 28.0 (4 x CH_2), 28.0 (4 x CH_2), 27.8 (4 x CH_2), 23.7 (CH_2), 14.5 (CH_3).

Compound 35 was synthesized by adapting the reported procedure in the reference S2. To a solution of **24** (11.00 mg, 0.03 mmol) in dry DMF (0.3 mL) under N_2 atmosphere, DIPEA (30 µL, 4.15 mmol) and HBTU (12.0 g, 3.30 mmol) were added. Then, **14** (67 mg, 0.02 mmol) was added, and the mixture was stirred at rt for 4 h. The crude product was dissolved in 5 mL of EtOAc, washed with sat. $NaHCO_3$ (1 × 10 mL), citric acid (20%, 1× 10 mL), and LiCl (5%, 2× 10 mL), dried over Na_2SO_4, and finally the solvent was evaporated under reduced pressure. The crude product was purified by column chromatography (SiO_2, CH_2Cl_2/MeOH 95:5) to afford **35** (42 mg, 55%) as a pale yellow solid. R_f (CH_2Cl_2/MeOH 95:5): 0.35; $[\alpha]_D^{20}$ -11.4 (*c* 1.0, $CHCl_3$); IR (neat): 3288 (br, N–H), 2977 (m, C–H), 2929 (m, C–H), 2856 (m, C–H), 1728 (s, C=O, ester), 1631 (s, C=O, amide), 1539 (m), 1446 (m, C–H), 1367 (m, C–H, t-Bu), 1253 (m, C–O), 1149 (s, C–O), 1041 (w, C–N), 964 (w,

C=C–H), 847 (w), 753 (w); ^{1}H NMR (400 MHz, CD_3OD): 5.36 – 5.33 (m, 2H), 4.57 – 4.51 (m, 2H), 4.46 – 4.31 (m, 11H), 4.21 – 4.17 (m, 1H), 4.14 – 4.07 (m, 2H), 4.01– 3.97 (m, 1H), 3.21 – 3.12 (m, 2H), 2.41 – 2.32 (m, 28H), 2.17 – 1.97 (m, 28H), 1.91 – 1.85 (m, 9H), 1.49 – 1.47 (m, 72H), 1.46 – 1.44 (m, 74H), 1.32 – 1.29 (m, 29H), 0.94 – 0.86 (m, 3H); ^{13}C NMR (101 MHz, CD_3OD): 174.9 (4 x C), 174.8 (2 x C), 174.7 (C), 174.6 (C), 174.5 (C), 174.4 (C), 174.3 (C), 174.2 (C), 173.6 (4 x C), 173.5 (4 x C), 173.4 (4 x C), 173.3 (C), 173.1 (2 x C), 173.0 (C), 172.9 (C), 172.8 (C), 172.5 (C), 171.3 (C), 130.9 (C), 130.8 (C), 83.6 (2 x C), 83.5 (C), 83.5 (C), 83.5 (C), 83.4 (C), 83.2 (C), 83.1 (C), 81.9 (2 x C), 81.8 (2 x C), 81.8 (2 x C), 81.8 (C), 81.7 (C), 81.6 (C), 63.5 (C), 54.2 (5 x CH), 53.8 (5 x CH), 53.8 (5 x CH), 40.7 (CH_2), 33.0 (4 x CH_2), 32.7 (6 x CH_2), 32.5 (6 x CH_2), 33.1 (CH_2), 32.7 (CH_2), 32.5 (CH_2), 30.9 (CH_2), 30.8 (CH_2), 30.8 (CH_2), 30.6 (2 x CH_2), 30.5 (CH_2), 30.5 (CH_2), 30.4 (CH_2), 30.3 (CH_2), 28.5 (5 x CH_3), 28.5 (7 x CH_3), 28.5 (10 x CH_3), 28.4 (10 x CH_3), 28.4 (5 x CH_3), 28.4 (6 x CH_3), 28.4 (5 x CH_3), 28.2 (3 x CH_2), 28.1 (4 x CH_2), 28.1 (3 x CH_2), 28.0 (3 x CH_2), 28.0 (3 x CH_2), 23.7 (CH_2), 14.5 (CH_3).

2.7. Synthesis of G3P-Flippers

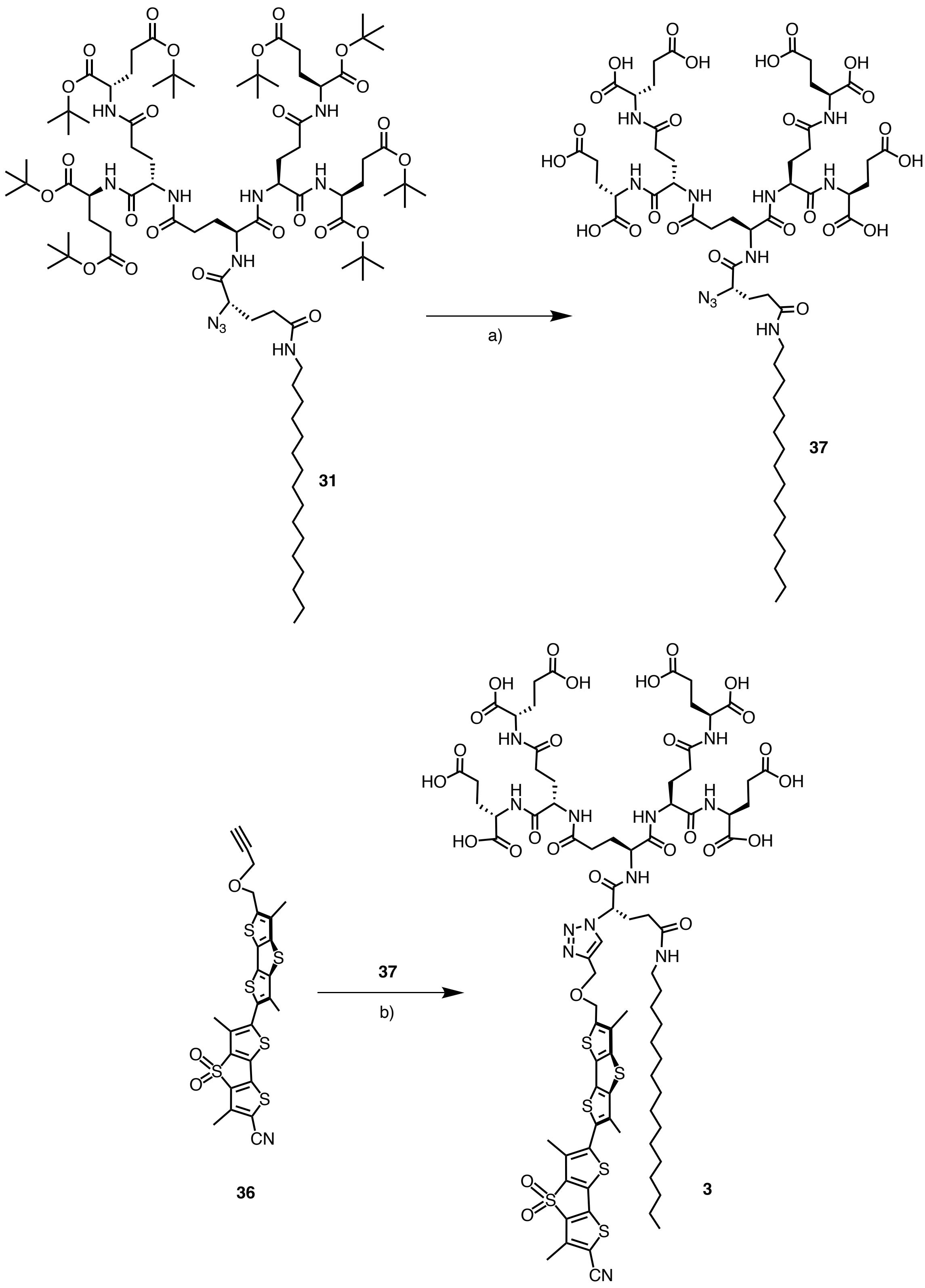

Scheme S7 Synthesis of G3P-Flippers **3**. a) TFA, CH_2Cl_2, rt, 5 h. b) **36**, THPTA, $CuSO_4 \cdot 5\ H_2O$, sodium ascorbate, CH_2Cl_2/DMSO/H_2O 1:2:1.3, rt, 5 h, 51%.

Compound 36 was synthesized according to reported procedures.[S7]

Compound 3. To a solution of **31** (66 mg, 0.039 mmol) in CH_2Cl_2 (1.0 mL), TFA (1.0 mL) was added, and the reaction was stirred at rt for 5 h. The volatiles were removed to obtain **37** a colorless solid. To a solution of this solid, (44 mg, 0.038 mmol), **36** (22.2 mg, 0.0390 mmol) and THPTA (16.4 mg, 0.0378 mmol) in DMSO (1.35 mL) and CH_2Cl_2 (0.7 mL), $CuSO_4$ $5H_2O$ (51.2 mg, 0.205 mmol) and sodium ascorbate (79 mg, 0.40 mmol) in H_2O (0.8 mL) were added. The reaction was stirred at rt for 5 h. The solvent was reduced *in vacuo,* and CH_2Cl_2 (2 mL) and basic water (1 mL of sat. $NaHCO_3$, 10 mL) were added. The aqueous layer was washed with CH_2Cl_2 (10 x 5 mL). The solvent was lyophilized, and the remaining solid was acidified with 1 mM aq. HCl until pH 1. The precipitate was triturated with water (2 × 2 mL), MeCN (10 × 2 mL) and hexane (3 × 2 mL) to afford **3** (36 mg, 51%) as a red solid. IR (neat): 3300 (br, O–H), 3076 (w), 2922 (m, C–H), 2851 (m, C–H), 2214 (w), 1715 (s, C=O), 1644 (s, amide C=O), 1538 (m, amide II), 1445 (m, C–H), 1412 (m), 1317 (m), 1204 (m, C–O), 1143 (m, C–O), 1092 (w), 1066 (w), 792 (w), 655 (w), 580 (w), 557 (w); ^1H NMR (400 MHz, DMSO-d_6): 8.68 (d, $^3J_{H-H}$ =7.5 Hz, 1H), 8.34 – 8.18 (m, 4H), 8.05 – 8.03 (m, 3H), 7.74 (s, 1H), 5.40 – 5.36 (m, 1H), 4.76 (s, 2H), 4.62 (s, 2H), 4.22 – 4.19 (m, 7H), 2.96 – 2.94 (m, 2H), 2.34 – 2.14 (m, 27H), 1.95 – 1.92 (m, 8H), 1.75 – 1.73 (m, 7H), 1.36 – 1.16 (m, 29H), 0.84 – 0.80 (m, 3H); ^{13}C NMR (126 MHz, DMSO-d_6): 173.8 (C), 173.7 (C), 173.7 (3 x C), 173.6 (C), 173.3 (C), 173.3 (C), 171.9 (3 x C), 171.6 (C), 171.5 (C), 171.0 (C), 170.4 (C), 167.7 (C), 143.9 (C), 143.5 (C), 143.4 (C), 142.2 (C), 141.8, 140.8 (C), 139.9 (C), 138.1 (C), 136.8 (C), 133.3 (C), 131.5 (C), 130.5 (C), 130.3 (C), 128.6 (C), 128.0 (C), 126.0 (C), 123.5 (CH), 113.1 (C), 109.9 (C), 64.5 (CH_2), 62.6 (CH_2), 62.2 (CH), 52.4 (CH_2), 52.2 (CH_2), 52.0 (CH_2), 51.2 (3 x CH_2), 51.1 (CH_2), 38.6 (CH_2), 31.7 (CH_2), 31.6 (CH_2), 31.4 (CH_2), 31.3 (2 x CH_2), 30.1 (CH_2), 30.1 (CH_2), 30.0 (CH_2), 30.0 (CH_2), 29.1 (5 x CH_2), 29.1 (3 x CH_2), 29.0 (CH_2), 28.8 (2 x CH_2), 28.7 (2 x CH_2), 28.4 (CH_2), 28.2 (CH_2), 28.1 (CH_2), 28.0 (CH_2), 26.5 (CH_2), 26.3 (2 x CH_2), 22.1 (CH_2), 14.0 (CH_3), 13.9 (CH_3), 12.6 (CH_3), 12.5 (CH_3), 12.1 (CH_3); HRMS (ESI-) calcd for $C_{81}H_{106}N_{12}O_{27}S_6$ $[M-2H]^{2-}$: 934.2734, found: 934.2769.

The purity of the compound was confirmed by reverse-phase high-performance liquid chromatography (RP-HPLC).

2.8. Synthesis of G3C-Flippers

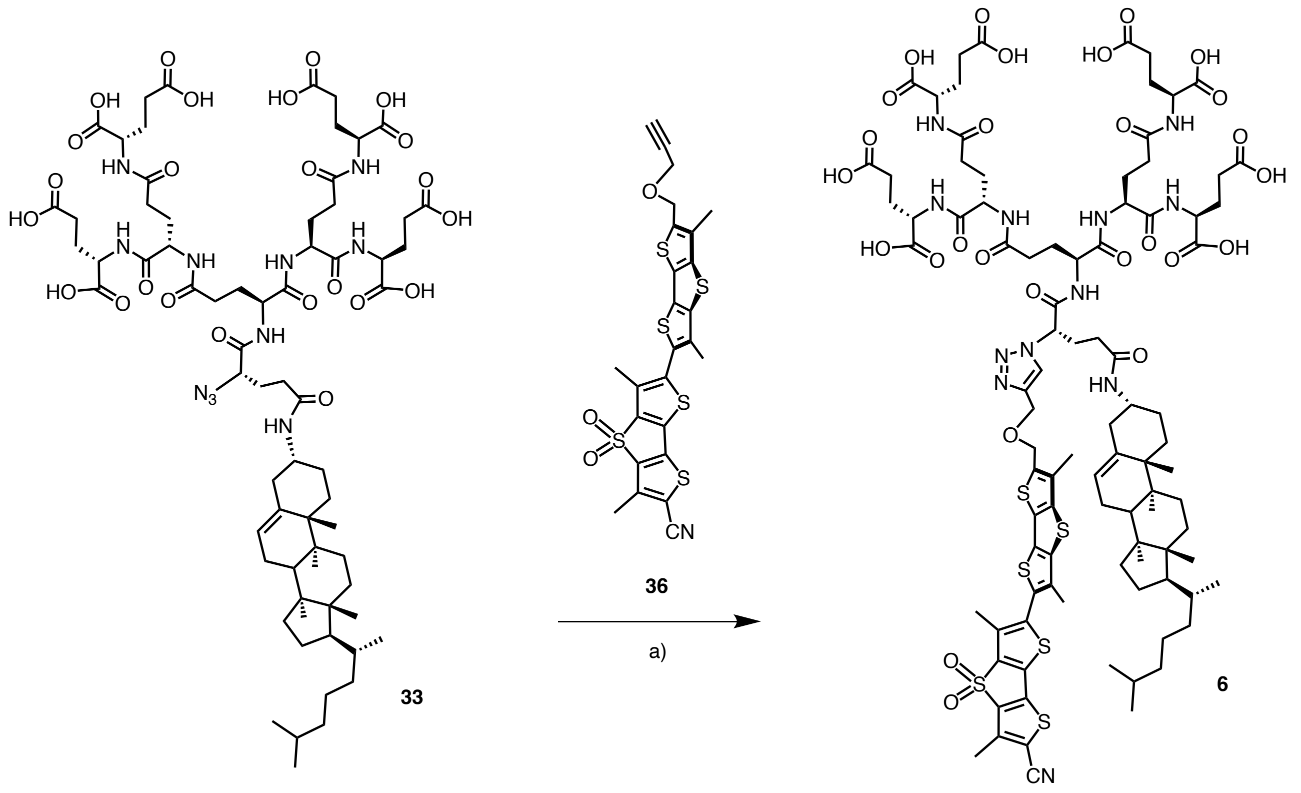

Scheme S8 Synthesis of G3C-Flippers **6**. a) **36**, THPTA, $CuSO_4 \cdot 5\ H_2O$, sodium ascorbate, CH_2Cl_2/DMSO/H_2O 5:4:3, rt, 5 h, 80%.

Compound 6. To a solution of **36** (3.2 mg, 5.6 µmol), **33** (6.4 mg, 4.5 µmol), and THPTA (5.8 mg, 13 µmol) in DMSO (0.7 mL) and CH_2Cl_2 (0.8 mL), $CuSO_4\ 5H_2O$ (2.8 mg, 11 µmol) and sodium ascorbate (4.9 mg, 25 µmol) in H_2O (0.2 mL) were added. The reaction was stirred at rt for 5 h. CH_2Cl_2 (10 mL) and basic water (1 mL of sat. $NaHCO_3$, 10 mL) were added. The aqueous layer was washed with CH_2Cl_2 (5 x 5 mL). The solvent was evaporated under reduced pressure, and the remaining solid was acidified with 1 mM aq. HCl until pH 1. The precipitate was triturated with water (4 × 5 mL), MeCN (3 × 1 mL) and CH_2Cl_2 (3 × 1 mL) to afford **6** (7.2 mg, 80%) as a red solid. IR

(neat): 3675 (br, w, O-H), 3058 (w, C-H), 2947 (w, C-H), 1737 (m, C=O), 1540 (w, C-H), 1424 (w, C-H), 1216 (m, C-O), 1054 (m, S=O), 1006 (s, C=C), 821 (m, C=C), 759 (m, C-H); ^{1}H NMR (500 MHz, DMSO-d_6): 12.39 (s, 8H), 8.69 (d, $^{3}J_{H-H}$ = 7.7 Hz, 1H), 8.26 – 8.22 (m, 4H), 8.11 – 7.98 (m, 4H), 7.18 (d, $^{3}J_{H-H}$ = 7.2 Hz, 1H), 5.44 – 5.38 (m, 1H), 5.19 (s, 1H), 4.75 (s, 2H), 4.63 (s, 2H), 4.27-4.14 (m, 7H), 3.90 (s, 1H), 2.54 (s, 3H), 2.41 – 0.73 (m, 106H), 0.89 (m, 3H), 0.81 (s, 3H), 0.80 (s, 3H), 0.57 (s, 3H); ^{13}C NMR (126 MHz, DMSO-d_6): 173.7 (C), 173.7 (C), 173.7 (C), 173.6 (C), 173.6 (C), 173.4 (C), 173.3 (C), 173.3 (C), 173.1 (C), 173.0 (C), 171.8 (C), 171.6 (C), 171.4 (C), 170.9 (C), 170.2 (C), 167.7 (C), 143.9 (C), 143.5 (C), 142.2 (C), 142.2 (C), 141.8 (C), 140.8 (C), 139.9 (C), 138.5 (C), 138.1 (C), 136.7 (C), 133.2 (C), 131.3 (C), 130.4 (C), 130.2 (C), 128.7 (C), 128.0 (C), 126.0 (C), 123.7 (CH), 121.9 (C), 113.0 (C), 109.8 (C), 64.1 (CH_2), 62.4 (CH_2), 62.1 (CH), 56.2 (CH), 55.6 (CH), 52.4 (CH), 52.2 (CH), 51.9 (CH), 51.2 (CH), 51.1 (CH), 49.1 (CH), 45.1 (CH), 41.7 (C), 40.4 (CH), 36.6 (C), 36.2 (CH_2), 35.6 (CH_2), 35.2 (CH), 33.1 (CH_2), 31.7 (CH_2), 31.6 (CH_2), 31.3 (CH_2), 31.2 (CH_2), 30.1 (CH_2), 30.1 (CH_2), 30.0 (CH_2), 30.0 (CH_2), 28.4 (CH_2), 28.2 (CH_2), 28.0 (CH_2), 27.8 (CH_2), 27.4 (CH), 26.3 (CH_2), 26.3 (CH_2), 25.6 (CH_2), 23.8 (CH_2), 23.4 (CH_2), 22.6 (CH_3), 22.4 (CH_3), 20.2 (CH_2), 18.6 (CH_3), 18.5 (CH_3), 13.9 (CH_3), 12.6 (CH_3), 12.5 (CH_3), 12.0 (CH_3), 11.6 (CH_3); HRMS (ESI-): calcd for $C_{92}H_{118}N_{12}O_{27}S_6$ $[M-2H]^{2-}$: 1006.3204, found: 1006.3197.

2.9. Synthesis of G4P- and G4O-Flippers

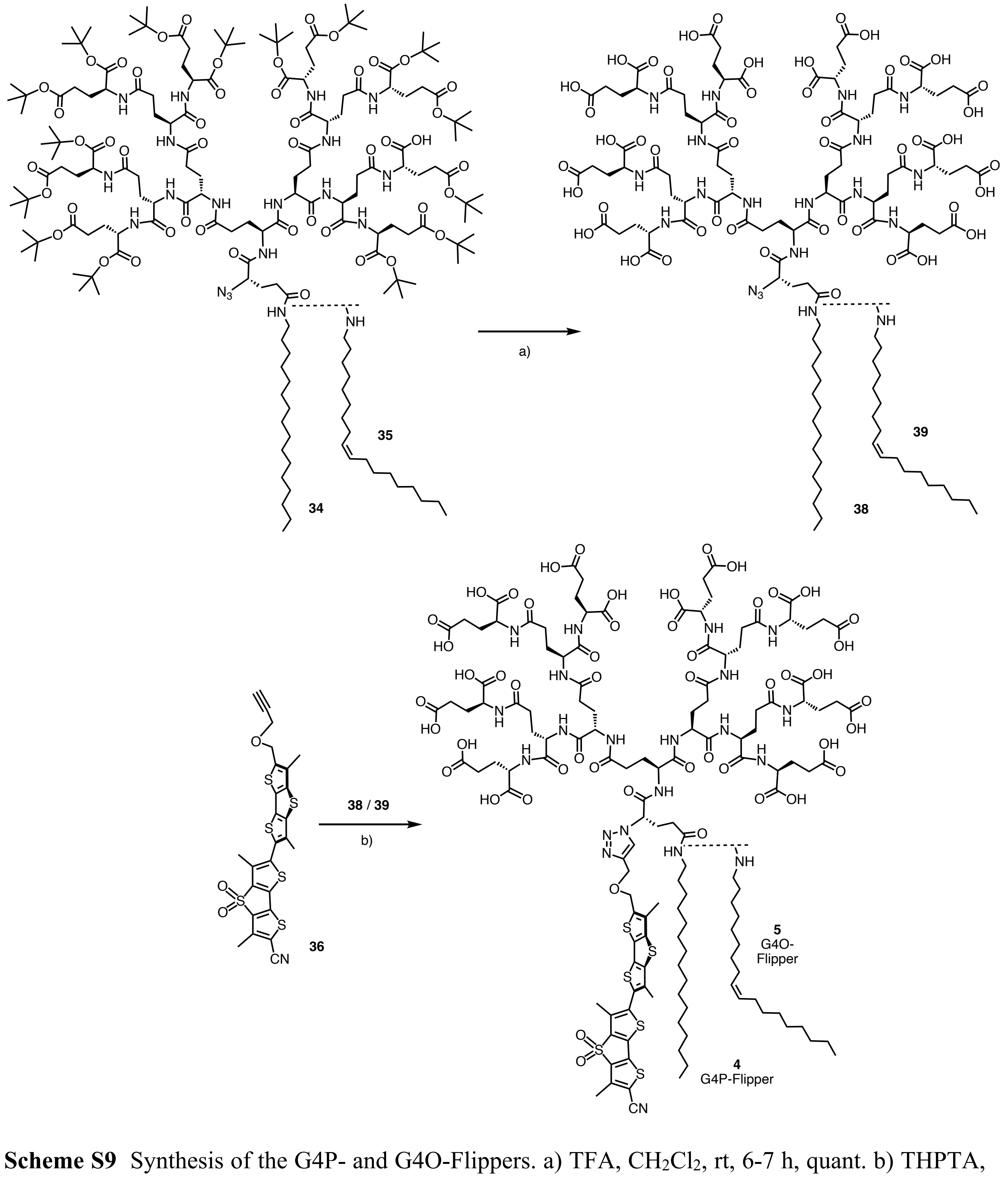

Scheme S9 Synthesis of the G4P- and G4O-Flippers. a) TFA, CH_2Cl_2, rt, 6-7 h, quant. b) THPTA, $CuSO_4 \cdot 5\ H_2O$, sodium ascorbate, dioxane/DMSO/H_2O 2:1:0.1, rt, 2-5 h, 30-49%.

Compound 4. To a solution of **34** (56 mg, 0.017 mmol) in CH_2Cl_2 (0.6 mL), TFA (0.8 mL) was added, and the reaction was stirred at rt for 7 h. The volatiles were removed to obtain a colorless

solid. To a solution of this solid (39.8 mg, 0.0173 mmol) in DMSO (0.5 mL) and **36** (19.5 mg, 0.0341 mmol) dissolved in dioxane (1.00 mL) were added THPTA (6.8 mg, 0.015 mmol), sodium ascorbate (33.8 mg, 0.171 mmol) and $CuSO_4$ 5·H_2O (20.9 mg, 0.0838 mmol) in H_2O (0.1 mL). The reaction was stirred at rt for 2 h. The solvent was reduced *in vacuo*, and CH_2Cl_2 (2 mL) and basic water (5 mL of sat. $NaHCO_3$) were added. The aqueous layer was washed with CH_2Cl_2 (10 x 5 mL). The solvent was evaporated under reduced pressure and the remaining solid was acidified with 1 mM aq. HCl until pH 1. The precipitate was triturated with water (2 × 2 mL), MeCN (10 × 2 mL) and hexane (3 × 2 mL) to afford **4** (25 mg, 49%) as a red solid. IR (neat): 3391 (br, w, O–H/N–H), 3004 (w, =C–H), 2919 (m, C–H), 1651 (m C=O), 1437 (m, C–H), 1407 (w), 1316 (w), 1013 (s, C–O), 951 (m), 705 (w, C–H); HRMS (ESI-): calcd for $C_{121}H_{162}N_{20}O_{51}S_6$ $[M-2H]^{2-}$: 1450.4437, found: 1450.4360. The purity of the compound was confirmed by reverse-phase high-performance liquid chromatography (RP-HPLC).

Compound 5. To a solution of **35** (83.0 mg, 0.0255 mmol) in CH_2Cl_2 (0.2 mL), TFA (0.6 mL) was added, and the reaction was stirred at rt for 6 h. The volatiles were removed to obtain a colorless solid. To a solution of this solid, (54.1 mg, 0.0255 mmol) in DMSO (0.5 mL) and **36** (19 mg, 0.033 mmol) dissolved in dioxane (1.00 mL) were THPTA (12 mg, 0.028 mmol), $CuSO_4$ 5·H_2O (5.7 mg, 0.023 mmol) and sodium ascorbate (10 mg, 0.05 mmol) in H_2O (0.2 mL). The reaction was stirred at rt for 4 h. The solvent was reduced *in vacuo,* and CH_2Cl_2 (2 mL) and basic water (1 mL of sat. $NaHCO_3$, 10 mL) were added. The aqueous layer washed with CH_2Cl_2 (10 x 5 mL). The solvent was evaporated under reduced pressure and the remaining solid was acidified with 1 mM aq. HCl until pH 1. The precipitate was triturated with water (2 × 2 mL), MeCN (10 × 2 mL) and hexane (3 × 2 mL) to afford **5** (20 mg, 30%) as a red solid. IR (neat): 3288 (br, w, N–H/O–H), 2977 (m, C–H), 2929 (m, C–H), 1727 (w), 1632 (s, amide C=O/C=C), 1541 (m), 1450 (m, C–H), 1392 (m), 1367 (m), 1252 (m, C–N/C–O), 1149 (s, C–O), 1025 (m, C–N), 992 (w, =C–H), 846 (w), 754 (w); HRMS (ESI-): calcd for $C_{123}H_{164}N_{20}O_{51}S_6$ $[M-2H]^{2-}$: 1463.4515, found: 1463.4612. The purity of the compound was assessed by reverse-phase high-performance liquid chromatography (RP-HPLC).

3. Fluorescence spectroscopy in solution

To a stirred PBS (2.0 mL) at 37 °C, the probe (**1** or **4**, 2 µL of a 1 mM stock solution in DMSO) was added. The excitation spectra were recorded at regular intervals for emission at λ_{em} = 650 nm, using a long-pass filter (O58) in the emission path. The spectrum of PBS was used as the background and subtracted.

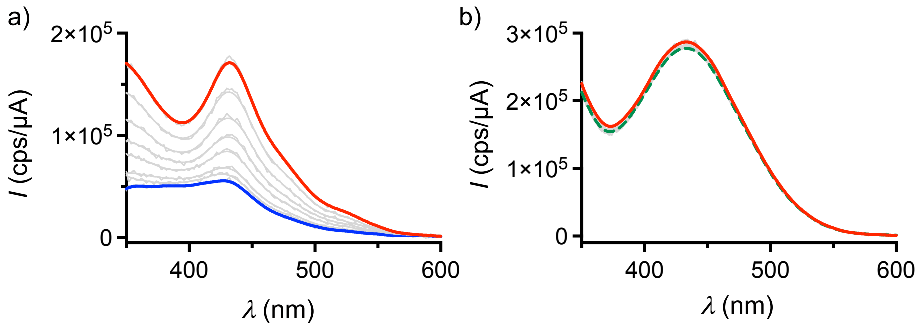

Fig. S2 Excitation spectra of flipper probes (a) **1** and (b) **4** in PBS as a function of time, immediately after the sample addition (red) to 30 min (green) or 65 min (blue).

4. Fluorescence spectroscopy in LUVs

Large unilamellar vesicles (LUVs) were prepared following reported procedures.[S1]

DOPC LUVs. A lipid film was prepared by evaporating a solution of DOPC (23 mg) in $CHCl_3$/MeOH 9:1 (1 mL) and then putting the flask under vacuum overnight. The resulting lipid film was hydrated with Tris buffer (10 mM Tris, 100 mM NaCl, pH 7.4) at rt for 30 min with occasional stirring. The mixture was subjected to freeze-thaw cycles (>10×, liquid N_2, 45 °C water bath and extruded (>15×) at rt through a polycarbonate membrane (pore size 100 nm) using a mini-extruder.

SM/CL LUVs were prepared following the procedure for DOPC LUVs using SM (14.8 mg) and CL (3.5 mg). Hydration (with 1 mL of Tris buffer) and extrusion were performed at 55 °C.

Fluorescence spectroscopy in LUVs: general procedure. To a stirred Tris buffer solution (2.0 mL, 10 mM Tris, 100 mM NaCl, pH 7.4) at 37.5 °C were added LUVs (5 µL of 30 mM lipid in Tris buffer) and then the probe (between 0.25 and 2 µL of a 0.1 mM stock solution in DMSO). The

emission and excitation spectra were recorded upon excitation at $\lambda_{ex} = 420$ nm and emission at $\lambda_{em} = 600$ nm, respectively. The same mixture without the probe was used as the background.

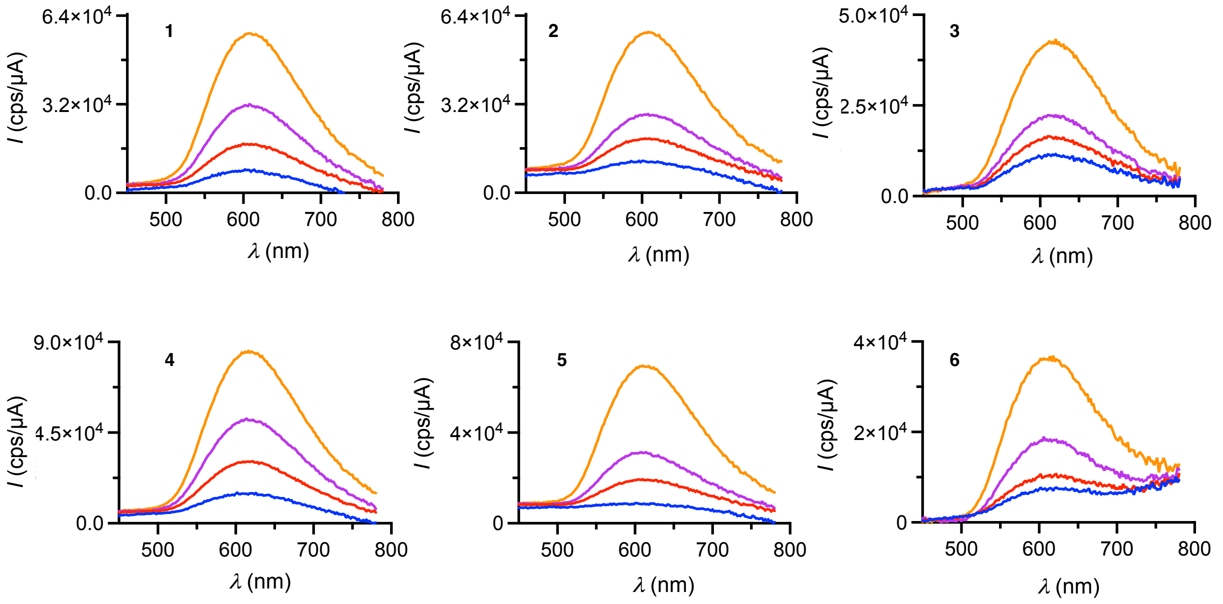

Fig. S3 Emission ($\lambda_{ex} = 420$ nm) spectra of flipper probes **1**, **2**, **3**, **4**, **5** and **6** in DOPC LUVs: 100 nM orange line, 50 nM purple line, 25 nM, red line and 12.5 nM blue line.

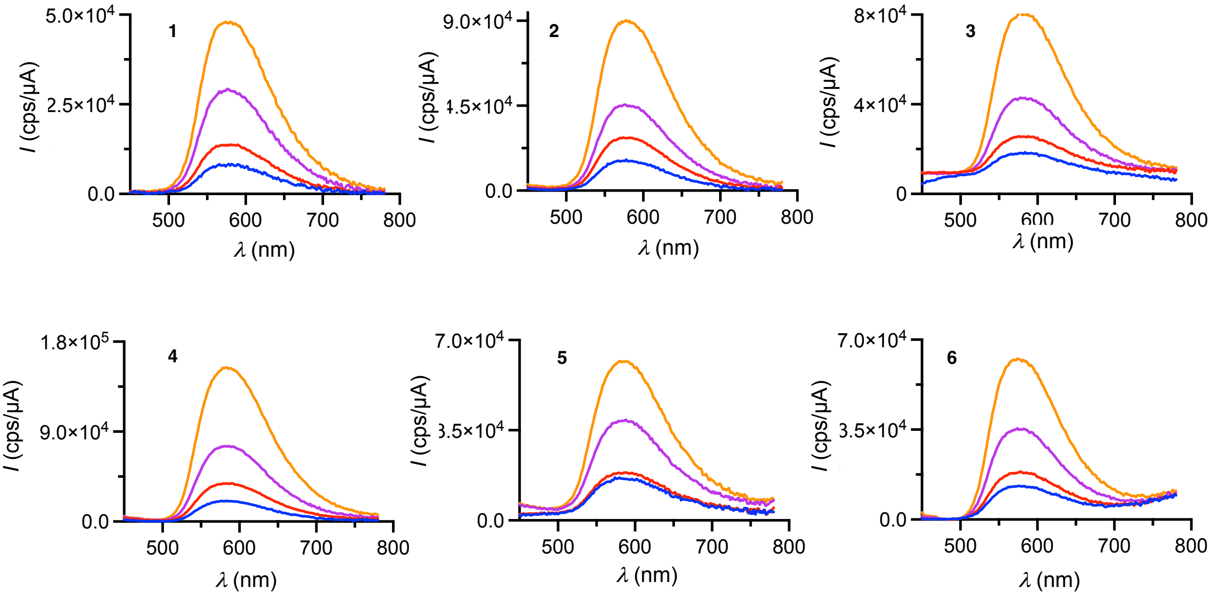

Fig. S4 Emission ($\lambda_{ex} = 420$ nm) spectra of flipper probes **1**, **2**, **3**, **4**, **5** and **6** in SM/CL LUVs: 100 nM orange line, 50 nM purple line, 25 nM, red line and 12.5 nM blue line.

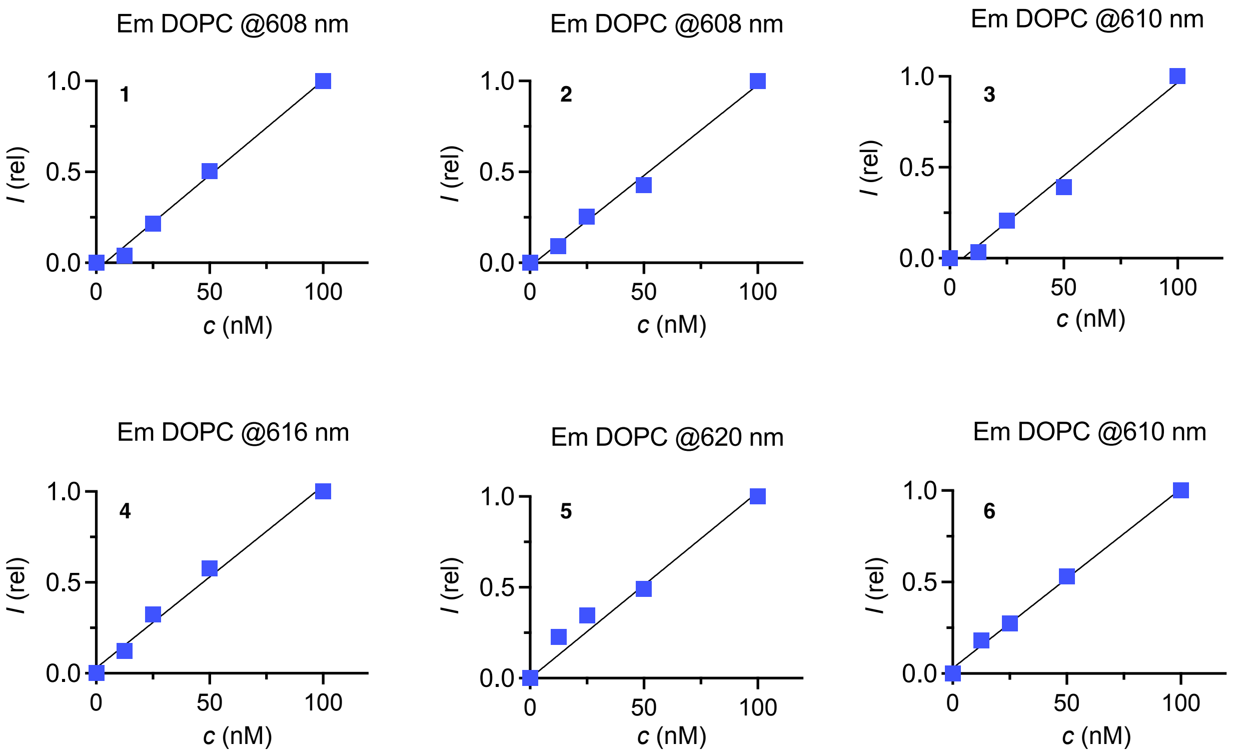

Fig. S5 Dependence of maximum emission intensities on concentrations of compounds: **1**, **2**, **3**, **4**, **5** and **6** in DOPC LUVs at 37 °C.

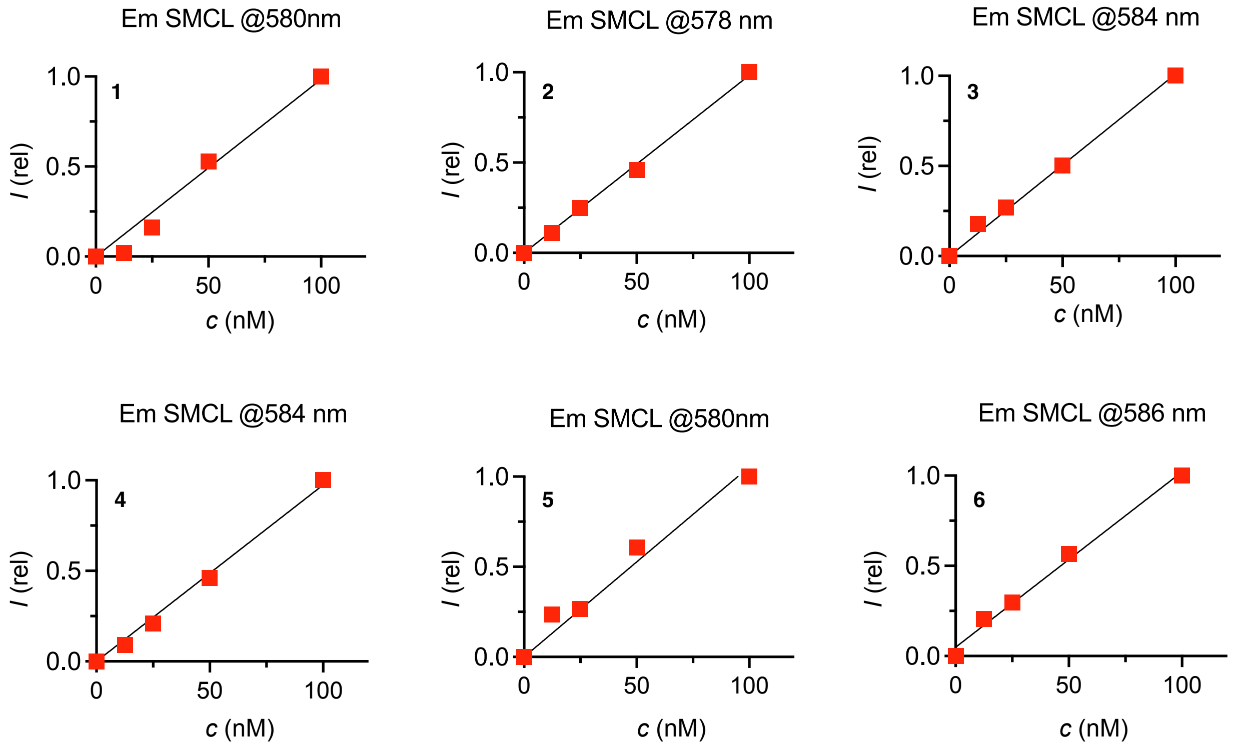

Fig. S6 Dependence of maximum emission intensities on concentrations of compounds: **1**, **2**, **3**, **4**, **5** and **6** in SM/CL 7:3 LUVs at 37 °C.

Table S1. Fluorescence properties of probes **1–6** in LUVs.

Cpd[a]	$\lambda_{ex\text{-}d}$ (nm)[b]	$\lambda_{em\text{-}d}$ (nm)[c]	$\lambda_{ex\text{-}o}$ (nm)[d]	$\lambda_{em\text{-}o}$ (nm)[e]	I_d (rel)[f]	I_o (rel)[g]
**1**	434	610	480	578	1.0	1.0
**2**	438	608	482	578	0.9	1.9
**3**	440	612	484	582	1.1	1.5
**4**	442	618	486	584	1.5	3.3
**5**	442	616	484	586	0.75	1.2
**6**	440	612	480	574	0.67	1.3

[a]Compounds **1-6** (100 nM). [b]Excitation maximum in DOPC LUVs. [c]Emission maximum in DOPC LUVs. [d]Excitation maximum in SM/CL 7:3 LUVs. [e]Emission maximum in SM/CL LUVs. [f]Relative intensities in DOPC LUVs at the emission maximum compared to compound **1**. [g]Relative intensities in SM/CL LUVs at the emission maximum compared to compound **1**.

4.1. Extraction with BSA

Samples were prepared following the general procedure using a stock solution of either DOPC or SM/CL LUVs (30 mM in Tris buffer, 5 µL) and the probe (0.1 mM in DMSO, 2 µL). After full equilibration (45 min), an increasing amount of a stock solution of BSA (0.5 mM in water, 1-160 µL) was added. Excitation spectra of Tris buffer solution (10 mM Tris, 100 mM NaCl, pH 7.4, 2 mL) at 37.5 °C were recorded to serve as background. Spectra were acquired two minutes after each addition of BSA. The excitation spectra of the probes in buffer with BSA (160 µL of 0.5 mM in H_2O) without LUVs were measured for comparison.

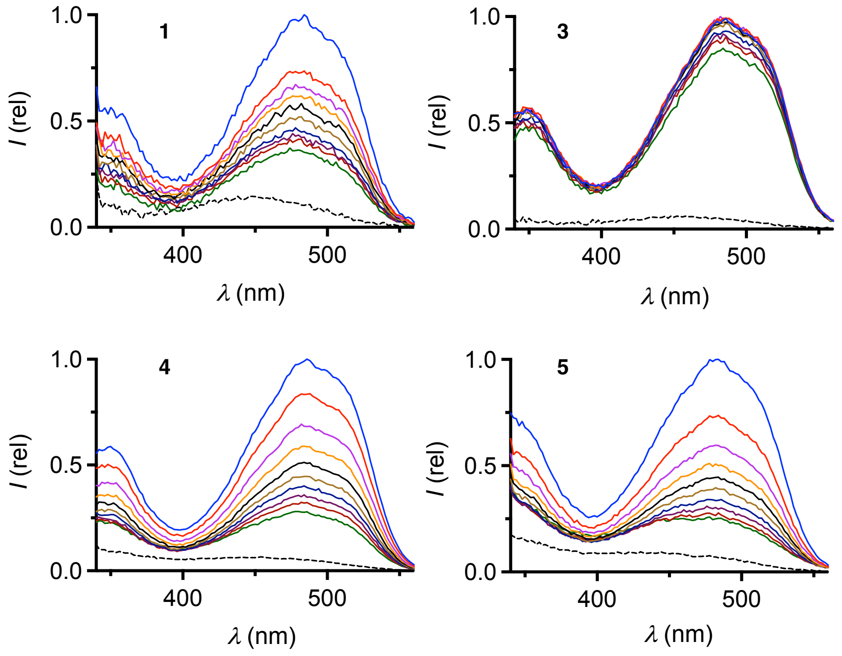

Fig. S7 Excitation spectra of the probes **1**, **3**, **4** and **5** in SM/CL 7:3 LUVs after the addition of 0 µM (blue), 0.25 µM (red), 0.5 µM (violet), 0.625 µM (orange), 1.25 µM (black), 2.5 µM (brown), 5 µM (dark blue), 10 µM (purple), 20 µM (dark red) and 40 µM (green) of BSA, and spectra of probes with BSA (40 µM, dashed) in buffer without LUVs.

Fluorescence intensities (I) at the emission maximum were extracted for each condition, normalized, and plotted as a function of BSA concentration (c) to generate dose–response curves. The data were fitted with Equation (S1) to determine the half-maximal effective concentration (EC_{50}) and the Hill coefficient (n).

$$I = I_{min} + [(1 - I_{min}) / (((EC_{50} / c) \wedge n) + 1)] \qquad (S1)$$

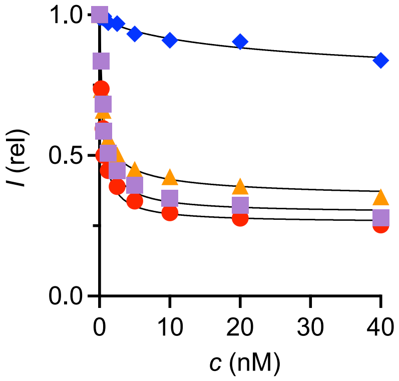

Fig. S8 Dose-response curves of the normalized excitation intensity at l_{ex} = 486 nm (Figure S7) as a function of BSA concentration for **1** (triangles), **3** (diamonds), **4** (squares), and **5** (circles).

4.2. Intermembrane exchange

Samples were prepared following the general procedure using SM/CL LUVs (30 mM in Tris buffer, 5 µL) and the probe (0.1 mM in DMSO, 2 µL). After full equilibration (45 min), DOPC LUVs (30 mM in Tris buffer, 5 µL) were added. The excitation spectra were recorded at 30-second intervals for 5 minutes.

4.3. Partition coefficients

Solutions were prepared following the general procedure. To a Tris buffer solution (10 mM Tris, 100 mM NaCl, pH 7.4, 2 mL), an increasing number of LUVs (0.5 – 40 µL of 30 mM in Tris buffer) were added. After equilibrating the mixture at 37.5 °C for 15 min, the probe (**1**, **2**, **3**, **4**, or **5**, 0.5 µL of 0.1 mM in DMSO) was added. Excitation spectra were acquired after complete equilibration (45 min) after the addition of the probes. Excitation spectra of samples without the probe at each lipid concentration served as background. K_x values were obtained by fitting the obtained fluorescence intensity I (λ_{em} = 600 nm, λ_{ex} DOPC = 440 nm, SM/CL = 478 nm) to the Equation (S2)

$$I = I_{min} + [(I_{max} - I_{min}) / (1 + (c_W / K_x \cdot c_l)] \qquad \text{(S2)}$$

where I_{min} is the intensity of the probe without LUVs added, I_{max} is the intensity of the probe with the highest LUV concentration, c_W is the concentration of water (55.6 M), c_l is the concentration of the LUVs added, and K_x is the partition coefficient.

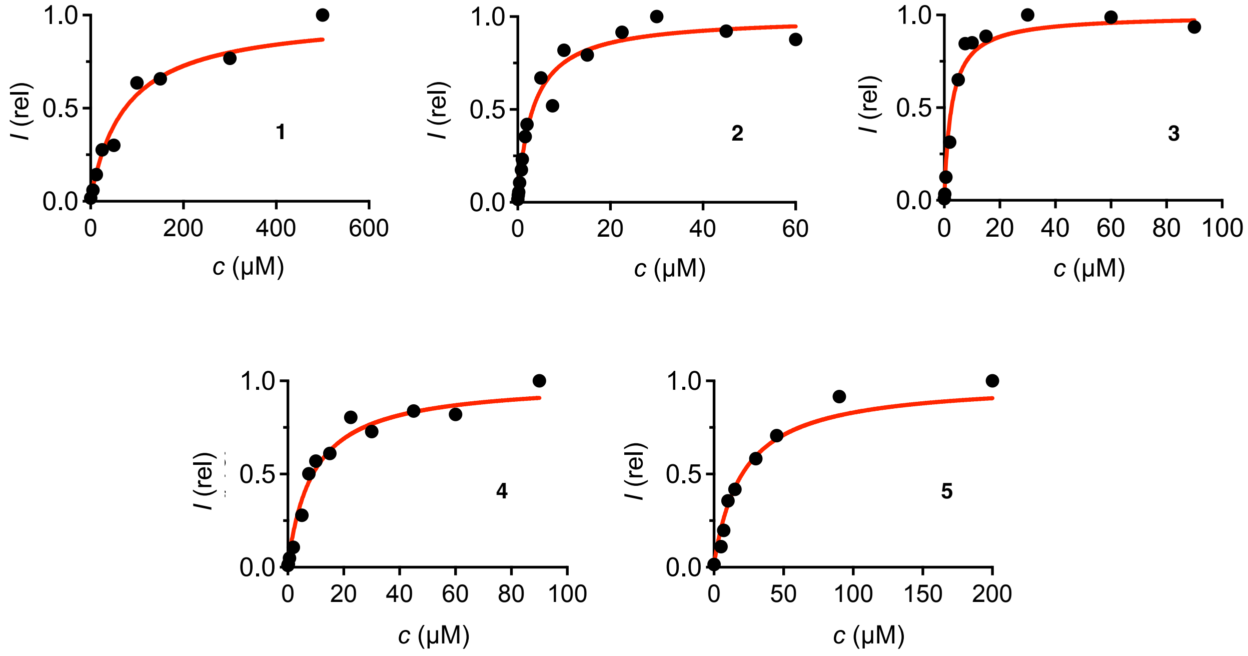

Fig. S9 Determination of the partition coefficients of the probe **1**–**5** (25 nM) in SM/CL 7:3 LUVs.

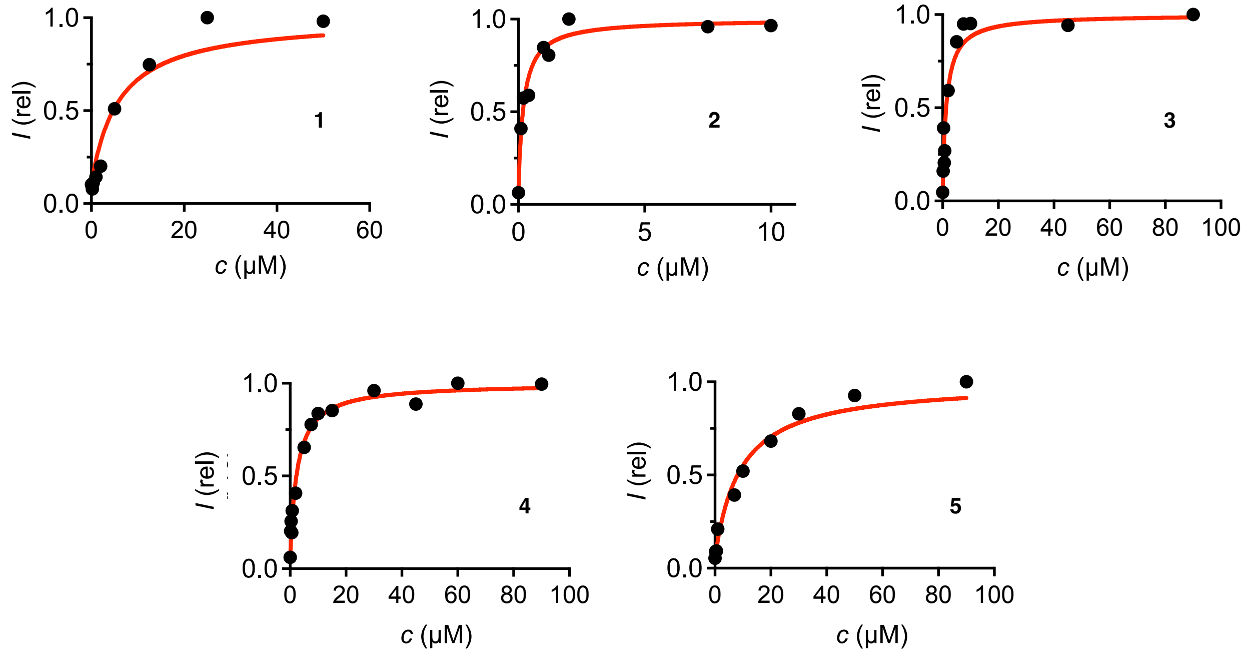

Fig. S10 Determination of the partition coefficients of the probe **1**–**5** (25 nM) in DOPC LUVs.

Table S2. Partition coefficients.

Cpd[a]	K_{xd} x 10^6 [b]	K_{xd} (rel)[c]	K_{xo} x 10^6 [d]	K_{xo} (rel)[e]	$K_{o/d}$[f]
**1**	9.4 ± 2[g]	1	0.72 ± 0.09[g]	1	0.07
**2**	270 ± 30[h]	29	17 ± 2[h]	23	0.06
**3**	39 ± 7	4	21 ± 3	29	0.53
**4**	22 ± 2	2.3	6.1 ± 0.6	8.4	0.3
**5**	6.1 ± 0.7	0.7	2.7 ± 0.3	3.8	0.4

[a]Compounds **1-5** (for the structures see Figure S1). [b]Partition coefficients in L_d DOPC LUVs. [c]K_{xd} results relative to compound **1**. [d]Partition coefficients in L_o SMCL LUVs. [e]K_{xo} results relative to compound **1**. [f]Ratio $K_{o/d} = K_{xo}/K_{xd}$. [g]Comparable values were reported previously (K_{xd} = 15 ± 4; K_{xo} = 1.1 ± 0.1).[S4] [h]Comparable values were reported previously (K_{xd} = 360 ± 60; K_{xo} = 41 ± 4).[S2]

4.4. Photobleaching kinetics

To a stirred Tris buffer solution (10 mM Tris, 100 mM NaCl, pH 7.4, 2.98 mL) in a plastic cuvette at 37 °C, SM/CL-LUVs (23 µL of 10 mM in Tris buffer) were added, followed by the probe (**1**, **3**, or **4**, 0.75 µL of 0.1 mM in DMSO). Excitation spectra (λ_{em} = 600 nm) were acquired every ≈5 min to confirm equilibrium was reached after 5–20 min. The sample was then irradiated at 450 nm with a LED (Royal Blue M450LP2, Thorlabs; placed at ~5 cm from the sample) at the maximum irradiance (34.2 µW/mm^2 according to the supplier) under stirring at rt. Excitation spectra were recorded every 10 min of irradiation.

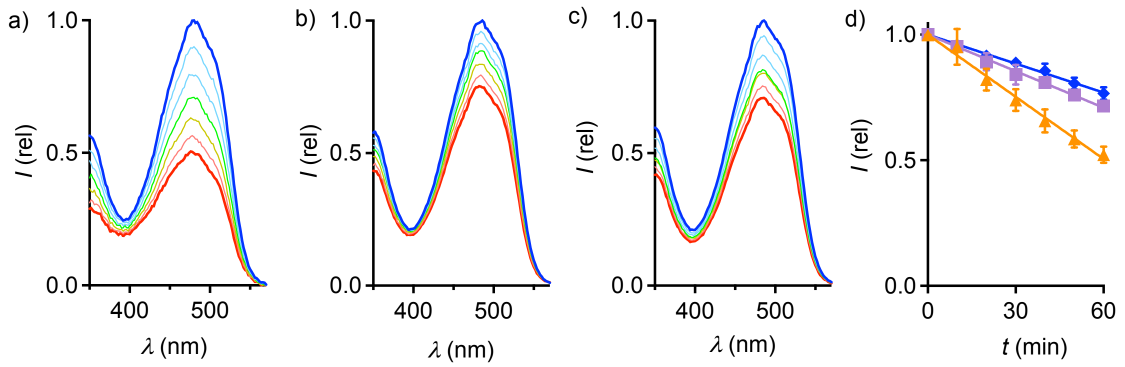

Fig. S11 a-c) Excitation spectra of probes (a) **1**, (b) **3**, and (c) **4** (all at 25 nM) in SM/CL 7:3 LUVs before (blue) and after repeated irradiation at 450 nm for 10 min (pale blue to red after 60 min). d) Normalized fluorescence intensities at the excitation maxima of **1** (orange triangles), **3** (blue diamonds), and **4** (purple squares, mean ± SEM of duplicates) as a function of irradiation time.

5. Fluorescence lifetime imaging microscopy of GUVs

GUVs were prepared by the electroformation method using a Nanion Vesicle Prep Pro following reported procedures.[S8]

DOPC GUVs. A thin lipid film was prepared by evaporating a solution of DOPC (10 mM) in $CHCl_3$ (20 µL) on the conductive side of an ITO electrode and drying under vacuum for 2 h. An o-ring covered in silicon grease was placed around the film, and 250 µL of an aqueous sucrose solution (250 mM) was added to the film. The second ITO electrode was placed on top of the first one, conductive side facing the joint and the sucrose solution. The electrodes were placed in the electroformation device and were exposed to an electric field of 1.2 V and 10 Hz for 2 h at 25 °C. This resulting stock solution of GUVs was used without further purification.

SM/CL GUVs. A thin lipid film was prepared by evaporating a preheated (55 °C) solution of SM/CL (7:3; 10 mM) in a mixture of $CHCl_3$ and MeOH (10/1, 20 µL) on the conductive side of a preheated ITO electrode (55 °C) and further drying under vacuum at 55 °C for 2 h. An o-ring covered in silicon grease was placed around the film, and 250 µL of an aqueous sucrose solution (250 mM) was added onto the film. The second ITO electrode was placed on top of the first one, conductive

side facing the joint and the sucrose solution. The electrodes were placed in the electroformation device and were exposed to an electric field of 1.2 V and 10 Hz for 2 h at 55 °C. This resulting stock solution of GUVs was used without further purification.

Phase-separated GUVs were prepared analogously to SM/CL GUVs using a solution of DOPC/SM/CL (58:25:17) in $CHCl_3$ with one drop of MeOH.

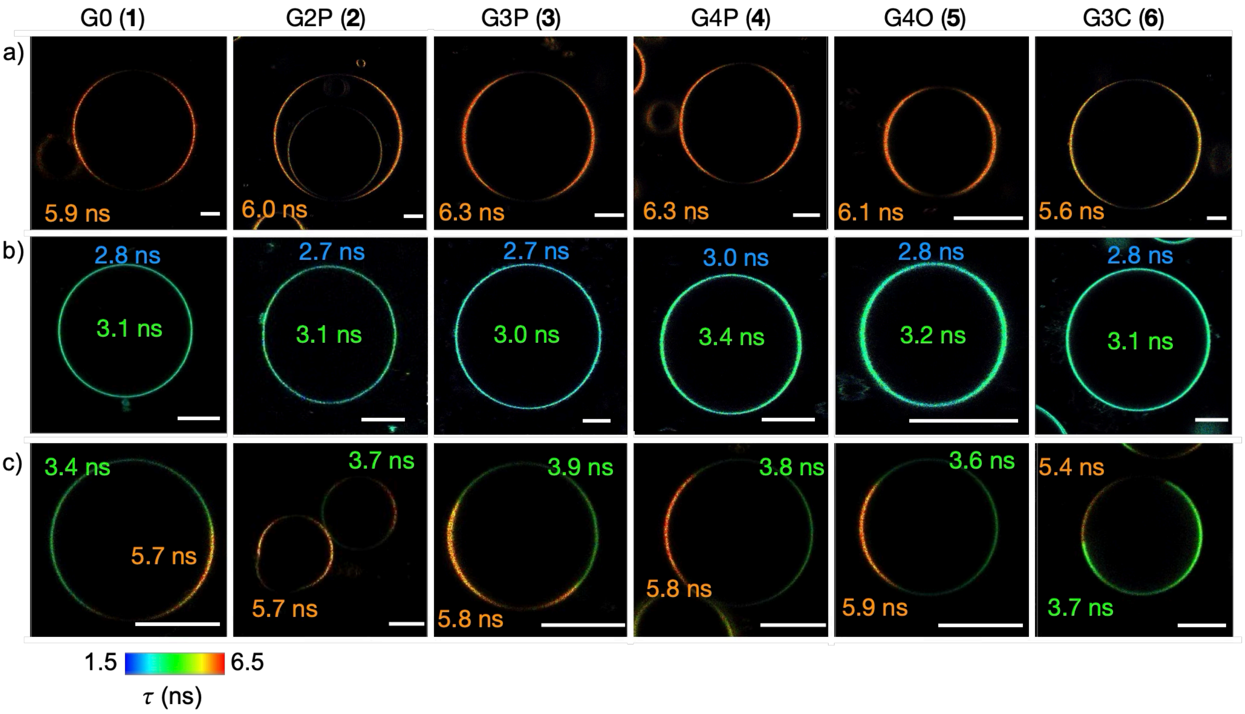

Fig. S12 Representative FLIM images of the probes **1–6** in a) SM/CL 7:3, b) DOPC, and c) mixed (DOPC/SM/CL 58:25:17) GUVs. Scale bars: 10 µm.

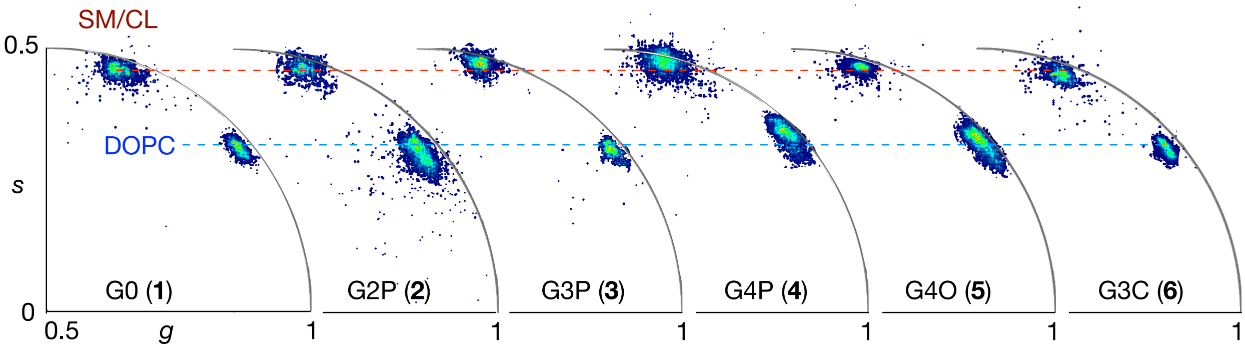

Fig. S13 Overlayed phasor plots (median) of the probes **1–6** in DOPC and SM/CL 7:3 GUVs.

Table S3. Fluorescent lifetimes, tilt angles, and intensity ratios of flippers **1–6** in GUVs.[a]

Cpd[b]	τ (ns)[c]				θ (°)[d]		I_{Lo}/I_{Ld}[e]
	DOPC	SM/CL	PS-L_d[f]	PS-L_o[f]	DOPC	SM/CL	
**1**	3.0	5.9	3.4	5.7	49 ± 1	21 ± 2	2.1 ± 0.3
**2**	3.0	6.0	3.7	5.7	49 ± 2	18 ± 2	1.9 ± 0.3
**3**	3.0	6.3	3.9	5.8	46 ± 2	17 ± 1	3.7 ± 0.5
**4**	3.3	6.4	3.8	5.8	45 ± 2	19 ± 1	3.4 ± 0.7
**5**	3.1	6.1	3.7	5.9	45 ± 1	18 ± 1	3.0 ± 0.8
**6**	3.0	5.6	3.7	5.4	48 ± 1	20 ± 2	0.7 ± 0.1

[a]Mean values ± SD from technical triplicates, unless noted. [b]Flipper probes. [c]Fluorescence lifetime near the equatorial positions of GUVs, estimated by manually choosing the top of the phasor cloud. SD < 0.1 ns. [d]Average tilt angle θ relative to the membrane normal. [e]Ratios of fluorescence intensities at the L_o phase over L_d phase in phase-separated GUVs. [f]L_d and L_o phases in phase-separated GUVs consisting of DOPC/SM/CL 58:25:17.

FLIM of GUVs. Samples were prepared by mixing 20 μL of GUVs, 280 μL of Tris buffer (10 mM Tris, 100 mM NaCl, pH 7.4) and a stock solution of the probe (1.5 μL of 0.12–0.26 mM **1**–**6** in DMSO) in a well of ibidi μ-slide 8-well glass bottom. The FLIM images were acquired using a Leica Stellaris 8 Falcon with a 63× oil immersion lens at rt. Fluorescence was collected between 550 and 650 nm upon excitation with a white laser set at 488 nm and 20 MHz. The images were analyzed using Leica Application Suite Software LASX FLIM 4.8. Lifetime (t) was estimated by manually choosing the top of the phasor cloud. Technical triplicates were performed, and the average values ± SD are reported.

The average tilt angle θ (in °, or v in radians) between the transition dipole of the probe and the membrane normal was estimated as described previously[S8,S9] from the ratio of intensities at the axial and equator positions of GUVs using Equation (S3)

$$F_{ax}/F_{eq} = (\tan^2 v)/2 \qquad \text{(S3)}$$

where F represents the intensity at the pole, or the equator minus background in the intensity images of FLIM.

6. Fluorescence lifetime imaging microscopy of cells

Cell preparation. As described in reference S10, HeLa Kyoto (HK) cells were seeded at 8 × 10^4 cells/mL (0.3 mL/well) in FluoroBrite™ DMEM (FDMEM) containing 10% fetal bovine serum (FBS), 1% Penicillin/Streptomycin (PS) and 1% GlutaMAX in an 8-well glass-bottomed µ-plate and cultured at 37 °C under 5% CO_2 for 1 day.

6.1. General procedure of FLIM imaging in HeLa Kyoto cells

The cells were rinsed (3 × 0.3 mL) with FDMEM (without FBS and PS) and incubated with FDMEM containing the probe (0.1–1.0 µM, 0.3 mL) for 5–100 min at 37 °C under 5% CO_2. The images were acquired without exchanging the medium using Leica Stellaris 8 Falcon with a 63× oil immersion lens at 37 °C under 5% CO_2. Fluorescence was collected between 550 and 650 nm upon excitation with a white light laser set at 488 nm and 20 MHz. The hypertonic shock was applied by first removing 0.15 mL of the medium and then adding 0.15 mL of 1 M aqueous sucrose solution in the well. Images were acquired after ~5 min under the conditions described above.

6.2. Kinetics

Following the general procedure, FLIM images were acquired at the indicated time after the addition of probes **1** and **4** (1.0 µM, from 0.75 µL of 0.4 mM stock solutions in DMSO).

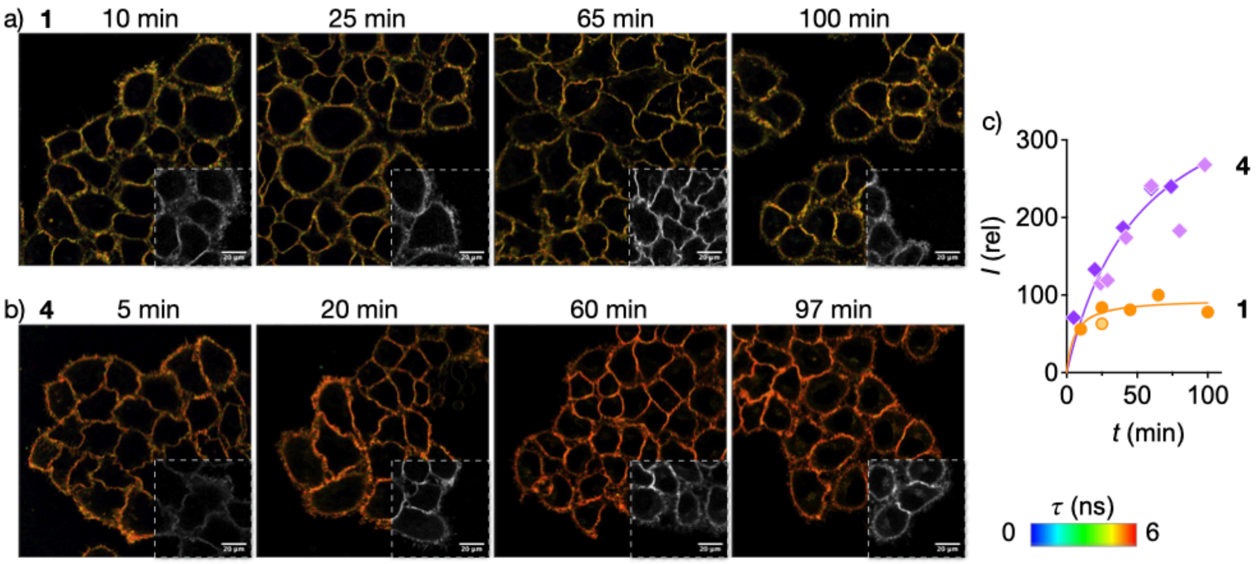

Fig. S14 a, b) Selected FLIM and confocal (inset) images of HK cells treated with probe a) **1** and b) **4** (1.0 μM) for the indicated time. LP = 0.05% (**1**) 0.02% (**4**). Same intensity scales were used for confocal images within the series, but not for FLIM images. c) Dependence of relative fluorescence intensities on the incubation time from experimental duplicates (different shades of orange (**1**) and purple (**4**)). Scale bars: 20 μm.

6.3. Concentration dependence

Following the general procedure, FLIM images were acquired after >1 h of incubation with probes **1** and **4** (0.1, 0.3, or 1.0 μM, from 0.55~0.75 μL of 40~400 μM stock solutions in DMSO).

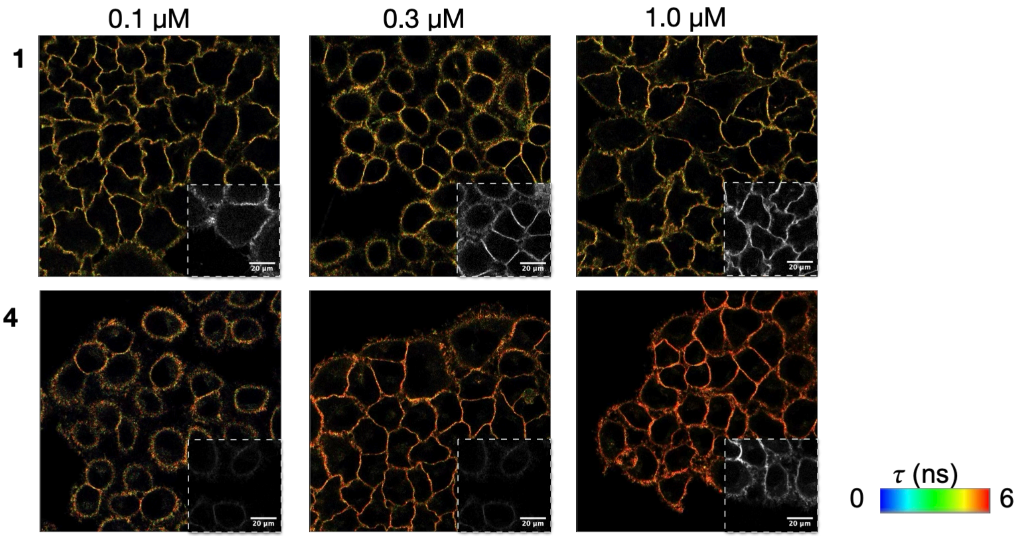

Fig. S15 Selected FLIM and confocal (inset) images of HK cells treated with probe **1** (top) and **4** (bottom) at different concentrations for >1 h. LP = 0.05% (**1**) 0.02% (**4**). Same intensity scales were used for confocal images of the same compound, but not for FLIM images. Note, the intensity of **4** was concentration dependent (see Fig. 2g). Scale bars: 20 μm.

6.4. Comparison of probes

Following the general procedure, FLIM images were acquired after >20 min of incubation with probes **1**–**6** (0.3 μM, from 0.75 μL of 0.12 mM stock solutions in DMSO). Experimental and technical triplicates were performed.

Plasma membrane selectivity (PM%) was estimated from the FLIM intensity images of probes **1**–**6** (0.3 μM, >20 min incubation). Intensities and the areas of whole cells (I_{all}, A_{all}), cell interior (combined I_{in}, A_{in}), and the background (I_{bg}, A_{bg}) were measured using Fiji, and converted to PM% by the Equation (S4)

$$PM\% = 100 \times [1 - (I_{in} - A_{in} \times I_{bg}/A_{bg})/ (I_{all} - A_{all} \times I_{bg}/A_{bg})] \qquad (S4)$$

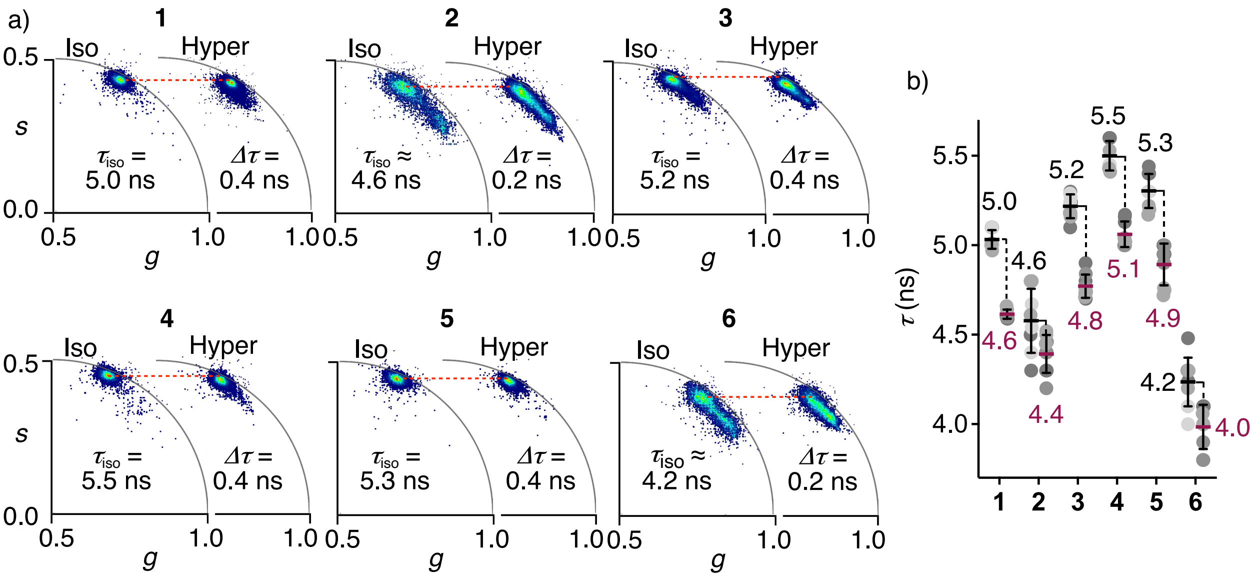

Fig. S16 a) Selected phasor plots derived from FLIM images of HK cells treated with probes **1**–**6** (0.3 μM) under isotonic and hypertonic conditions. b) Lifetimes (black: isotonic, dark red: hypertonic conditions; mean ± SD) estimated by manually choosing the top of the phasor cloud corresponding to plasma membrane staining. Each point represents a result from one image, experimental triplicates in different shades of grey.

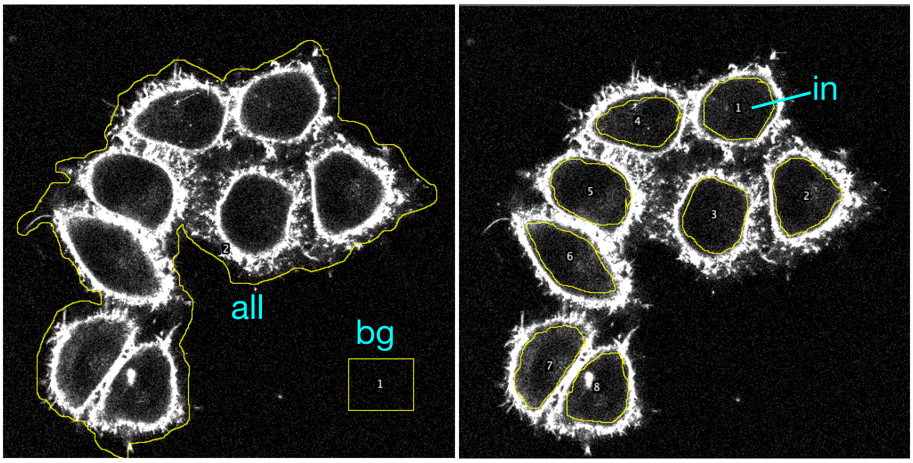

Fig. S17 Representative examples of whole cells (all), background (bg), and cell interiors (in), used for the calculation of PM%.

Table S4. Fluorescent lifetimes under isotonic and hypertonic conditions, relative intensities, and PM selectivities of flippers **1–6** in HK cells.[a]

Cpd[b]	τ_{iso} (ns)[c]	τ_{hyper} (ns)[c]	$\Delta\tau$ (ns)[c]	I / I_0[d]	*PM* (%)[e]
**1**	5.0 ± 0.1	4.6 ± 0.1	0.4	1.0	87 ± 2
**2**	4.6 ± 0.2	4.4 ± 0.1	0.2	0.06 ± 0.02	53 ± 3
**3**	5.2 ± 0.1	4.8 ± 0.1	0.4	0.7 ± 0.3	78 ± 2
**4**	5.5 ± 0.1	5.1 ± 0.1	0.4	2.3 ± 0.6	85 ± 2
**5**	5.3 ± 0.1	4.9 ± 0.1	0.4	1.1 ± 0.2	90 ± 1
**6**	4.2 ± 0.1	4.0 ± 0.1	0.2	0.08 ± 0.01	55 ± 7

[a]Mean values ± SD from technical triplicates. [b]Flipper probes. [c]Fluorescence lifetime estimated by manually choosing the top of the phasor cloud. [d]Fluorescence intensity relative to that of G0 (**1**) = I_0. [e]Plasma membrane selectivity.

6.5. Phototoxicity evaluation

Following the general procedure, FLIM images were acquired using Leica Stellaris 8 Falcon upon excitation with a white light laser set at 488 nm and 20 MHz after >60 min of incubation with probes **1** and **4** (1.0 μM). Images were acquired at 200 Hz with 8 line repetitions with a pixel dwell time of 7.6 μs. Laser powers (0.2%: 0.08 μW, 0.02%: 0.01 μW) were set to give comparable mean intensity counts per pixel for **1** (58) and **4** (43) in PM.

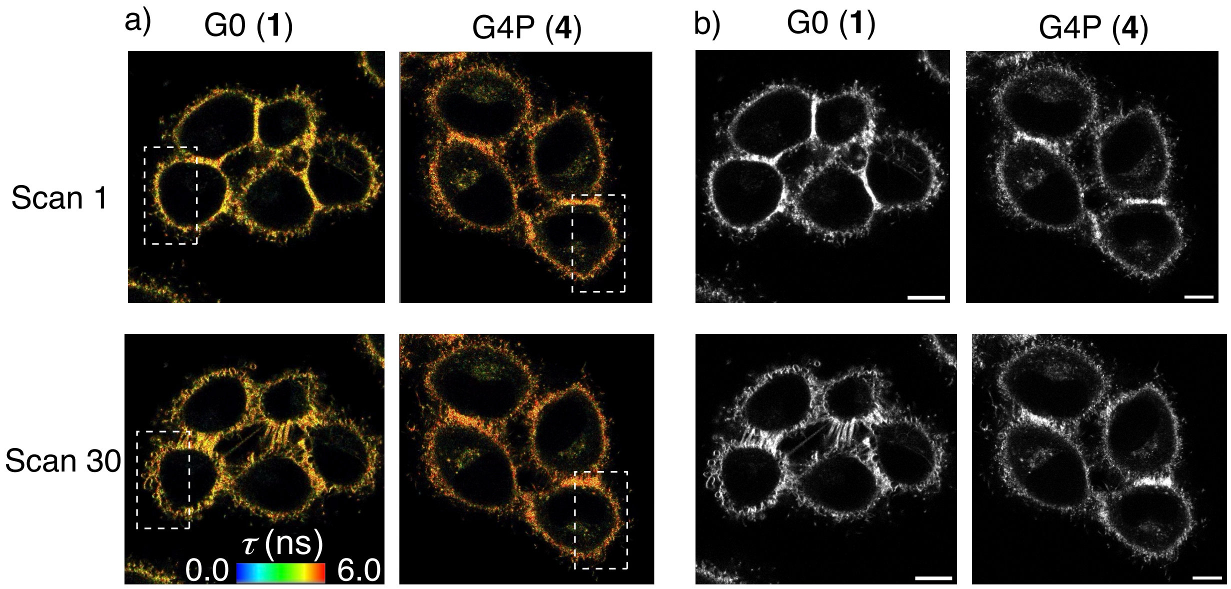

Fig. S18 Full a) FLIM (0-6 ns) and b) intensity (0-90 counts) images of HK cells treated with G0 (**1**, LP = 0.2%) or G4P (**4**, LP = 0.02%) after 1 or 30 scans. Zoomed images of regions in white dashed squares are shown in Figure 2i. Scale bars: 10 µm.

Phototoxicity was further evaluated in independent experiments. HeLa Kyoto cells were cultured in DMEM supplemented with 10% FBS and penicillin–streptomycin at 37 °C and 5% CO_2. Before imaging, the medium was replaced with FluoroBrite (without FBS, with PenStrep) containing probes **1** or **4** (both 1 µM). Flipper-TR **1** was incubated for 15 min, whereas Flipper G4P **4** was incubated for 1 h. Cells were maintained at 37 °C and 5% CO_2 throughout the incubation and imaging.

Fluorescence lifetime imaging was performed on a Nikon Eclipse Ti2 inverted microscope equipped with a point-scanning A1 confocal system, Apo LWD λ 40×/1.15 WI objective (#MRD77410), and a perfect focus system. The setup included a stage-top OkoLab incubation chamber. Excitation was provided by a pulsed 485 nm diode laser (PicoQuant LDH-D-C-485) driven by a Sepia PDL-828, operated at 20 MHz. Imaging parameters: pixel dwell 3.16 µs, scan speed ~100 Hz, pinhole 1.2 Airy Units (AU). Emission was filtered through a 600/50 nm bandpass and detected with a PMA Hybrid 40 detector. Photon arrival times were recorded with a Picoquant MultiHarp 150 TCSPC module. Control and acquisition were via NIS-Elements AR 3.30.05. No IRF measurement was used as input, but software generated. The laser power for Flipper-TR (80%, 40 µW) was set

approximately sevenfold higher than for Flipper G4P (11%, 5.5 μW) to match the measured 6.9× difference in brightness (mean intensity/cell pixel: 57 for **1**, 37 for **4**). Cells were imaged every 2 min for 20 min while maintaining physiological temperature and CO_2.

Cell area was segmented using an ilastik model[S11] applied identically to both probe conditions and normalized to the first time point to monitor percentage area changes. Intensity-weighted lifetime ("Fast lifetime", τ_{fast}) was computed as $\sum(\tau \cdot I)/\sum I$ within the segmented area.[S12] All processing was performed in a custom-made Jupyter Python notebook (https://jupyter.org/), and statistical comparisons between timepoint 0 and timepoint 10 were carried out using paired two-sided t-tests (ttest_rel, SciPy library) for both area and lifetime.

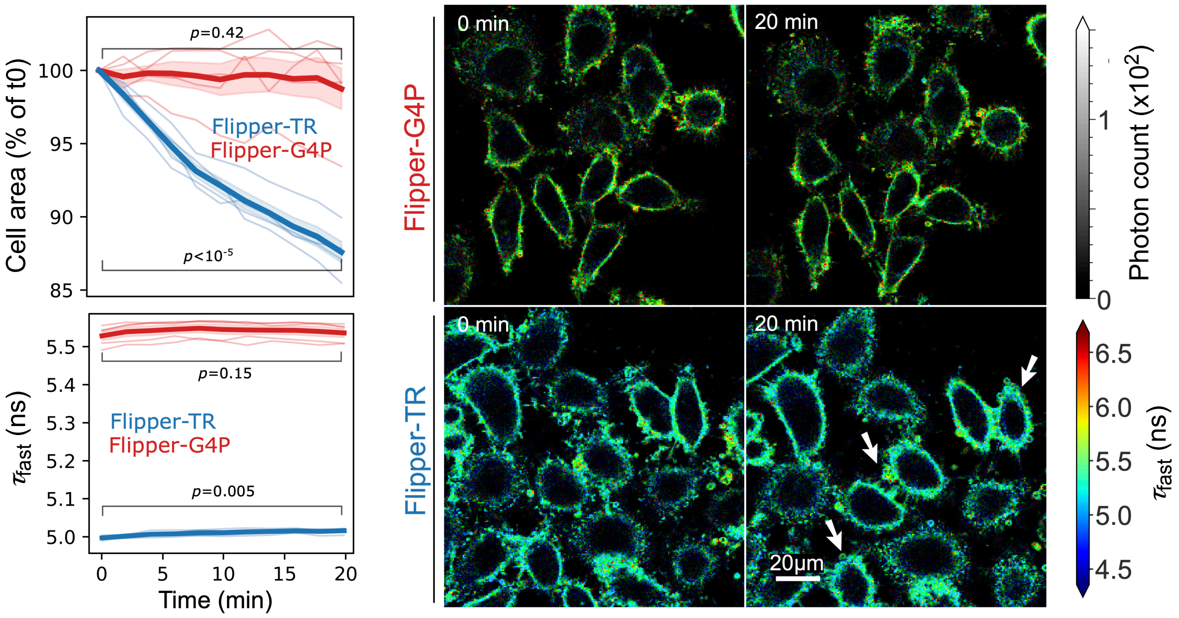

Fig. S19 Representative FLIM images of HK cells treated with Flipper-TR **1** (LP = 80%) or G4P **4** (LP = 11%) after 0 or 20 min, imaging every 2 min, arrows: blebbing. Determined from these images, cell area (top) and fluorescence lifetime τ_{fast} (bottom) as a function of imaging time (mean in bold, thin lines individual time lapse, *t*-test *p* values are between time point 0 and time point 11 (20 minutes at 2 min/frame).

7. Supplementary references

S1 J. García-Calvo, J. Maillard, I. Fureraj, K. Strakova, A. Colom, V. Mercier, A. Roux, E. Vauthey, N. Sakai, A. Fürstenberg and S. Matile, *J. Am. Chem. Soc.*, 2020, **142**, 12034–12038.

S2 F. Bayard and S. Matile, *Helv. Chim. Acta*, 2024, **107**, e202400062.

S3 Q. Xu, C. Shu, Y. Li, R. Zhuang, L. Jiang and X. Xu, *Chem. Commun.*, 2021, **57**, 4859–4862.

S4 F. Bayard, X. Chen, J. M. García-Arcos, A. Roux, N. Sakai and S. Matile, *ChemistryEurope*, 2023, **1**, e202300041.

S5 K. Suthagar and A. J. Fairbanks, *Chem. Commun.*, 2017, **53**, 713–715.

S6 J. De Vrieze, A. P. Baptista, L. Nuhn, S. Van Herck, K. Deswarte, H. Yu, B. N. Lambrecht and B. G. De Geest, *Adv. Ther.*, 2021, **4**, 2100079.

S7 S. Soleimanpour, A. Colom, E. Derivery, M. Gonzalez-Gaitan, A. Roux, N. Sakai and S. Matile, *Chem. Commun.*, 2016, **52**, 14450–14453.

S8 K. K. P. Pamungkas, I. Fureraj, L. Assies, N. Sakai, V. Mercier, X. Chen, E. Vauthey and S. Matile, *Angew. Chem. Int. Ed.*, 2024, **63**, e202406204.

S9 W. Grudzinski, J. Sagan, R. Welc, R. Luchowski and W. I. Gruszecki, *Sci. Rep.*, 2016, **6**, 32780.

S10 X. Chen, R. M. Gomilla, J. M. García-Arcos, M. Vonesch, N. Gonzalez-Sanchis, A. Roux, N. Sakai and S. Matile, *JACS Au*, 2023, **3**, 2557-2565

S11 S. Berg, D. Kutra, T. Kroeger, C. N. Straehle, B. X. Kausler, C. Haubold, M. Schiegg, J. Ales, T. Beier, M. Rudy, K. Eren, J. I. Cervantes, B. Xu, F. Beuttenmueller, A. Wolny, C. Zhang, U. Koethe, F. A. Hamprecht and A. Kreshuk, *Nat. Met.*, 2019, **16**, 1226–1232.

S12 T. Mandal, A. Roux and J. M. García-Arcos, *bioRxiv*, 2025, 2025.09.23.678124.

8. RP-HPLC chromatograms

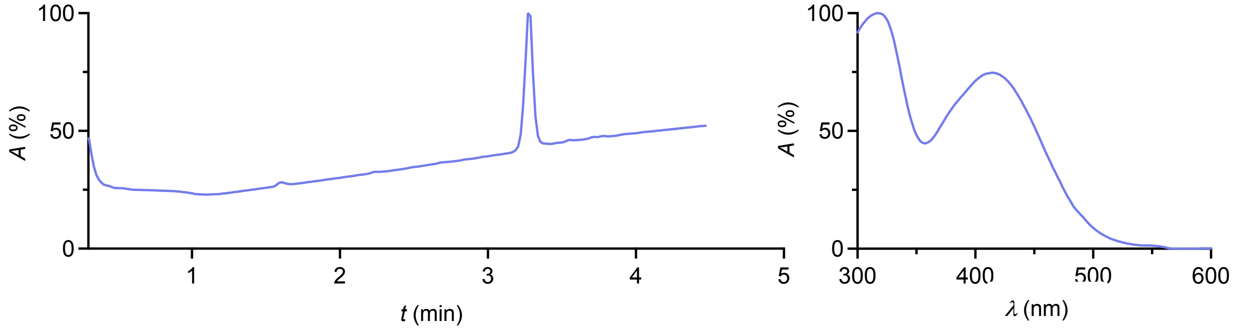

Fig. S20 Left: HPLC chromatogram (λ = 200 - 795 nm) of compound **3** (eluent H_2O/CH_3CN + 0.1% FA, from 40:60 to 5:95 in 4.5 min, flowrate 0.7 mL/min). Right: UV-vis absorption spectrum of peak at 3.20 min.

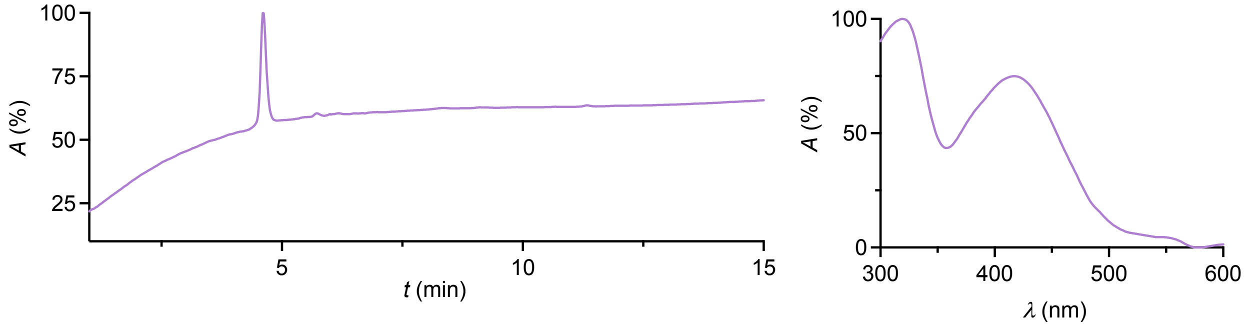

Fig. S21 Left: HPLC chromatogram (λ = 200 - 795 nm) of compound **4** (eluent H_2O/CH_3CN + 0.1% FA, from 70:30 to 22:78 in 15 min, flowrate 0.5 mL/min). Right: UV-vis absorption spectrum of peak at 4.37 min.

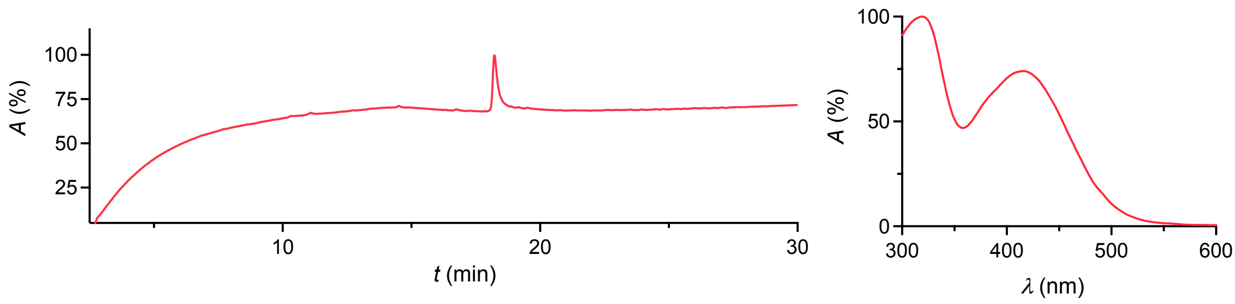

Fig. S22 Left: HPLC chromatogram (λ = 200 - 795 nm) of compound **5** (eluent H_2O/CH_3CN + 0.1% FA, from 70:30 to 5:95 in 30 min, flowrate 0.5 mL/min). Right: UV-vis absorption spectrum of peak at 18.15 min.

9. NMR spectra

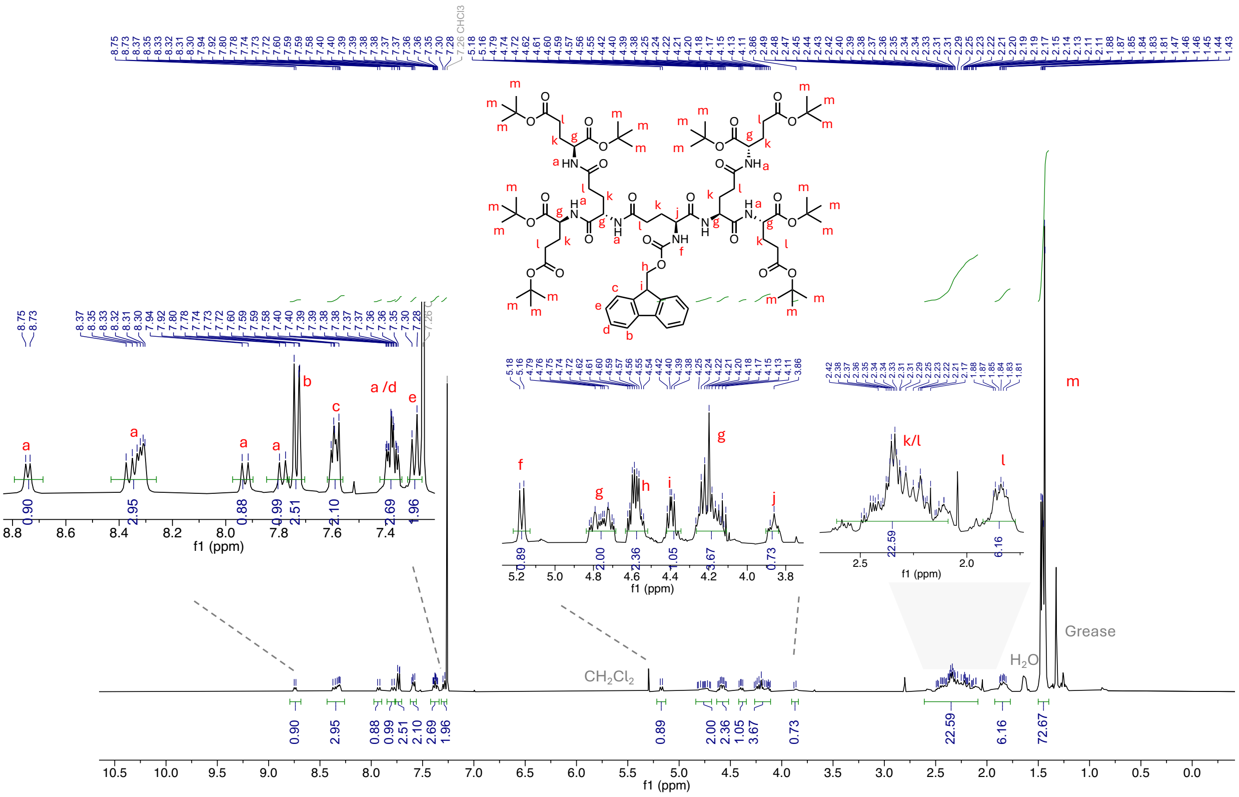

Fig. S23 400 MHz ^{1}H NMR spectrum of compound **11** in $CDCl_3$.

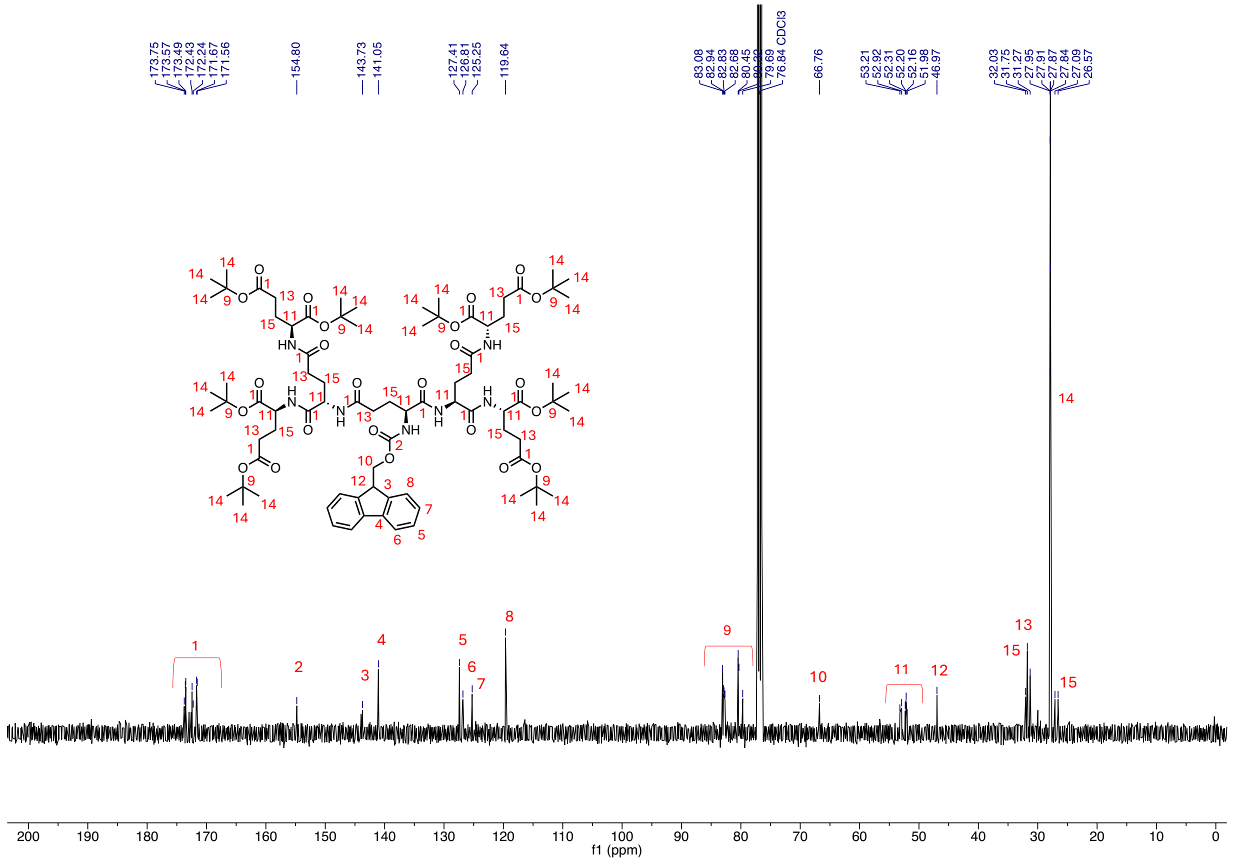

Fig. S24 101 MHz ^{13}C NMR spectrum of **11** in $CDCl_3$.

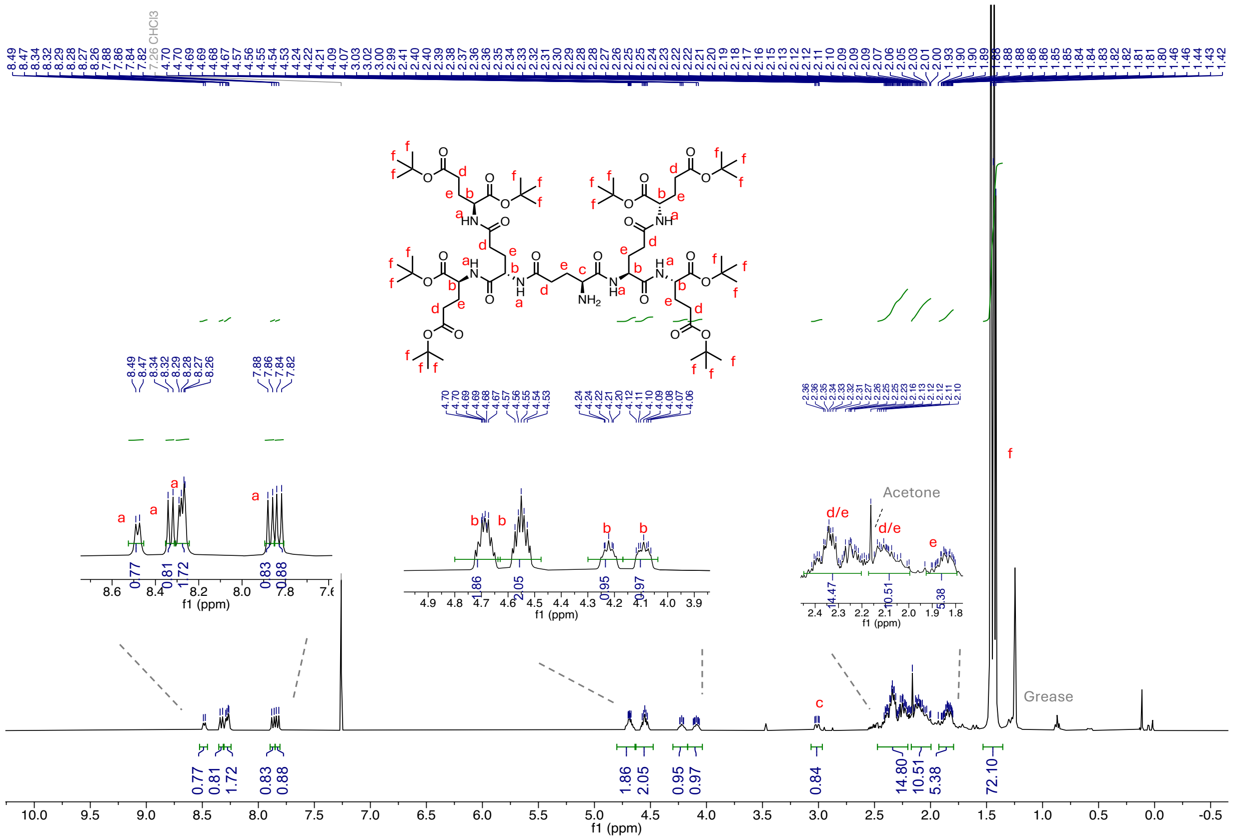

Fig. S25 400 MHz ^{1}H NMR spectrum of **12** in $CDCl_3$.

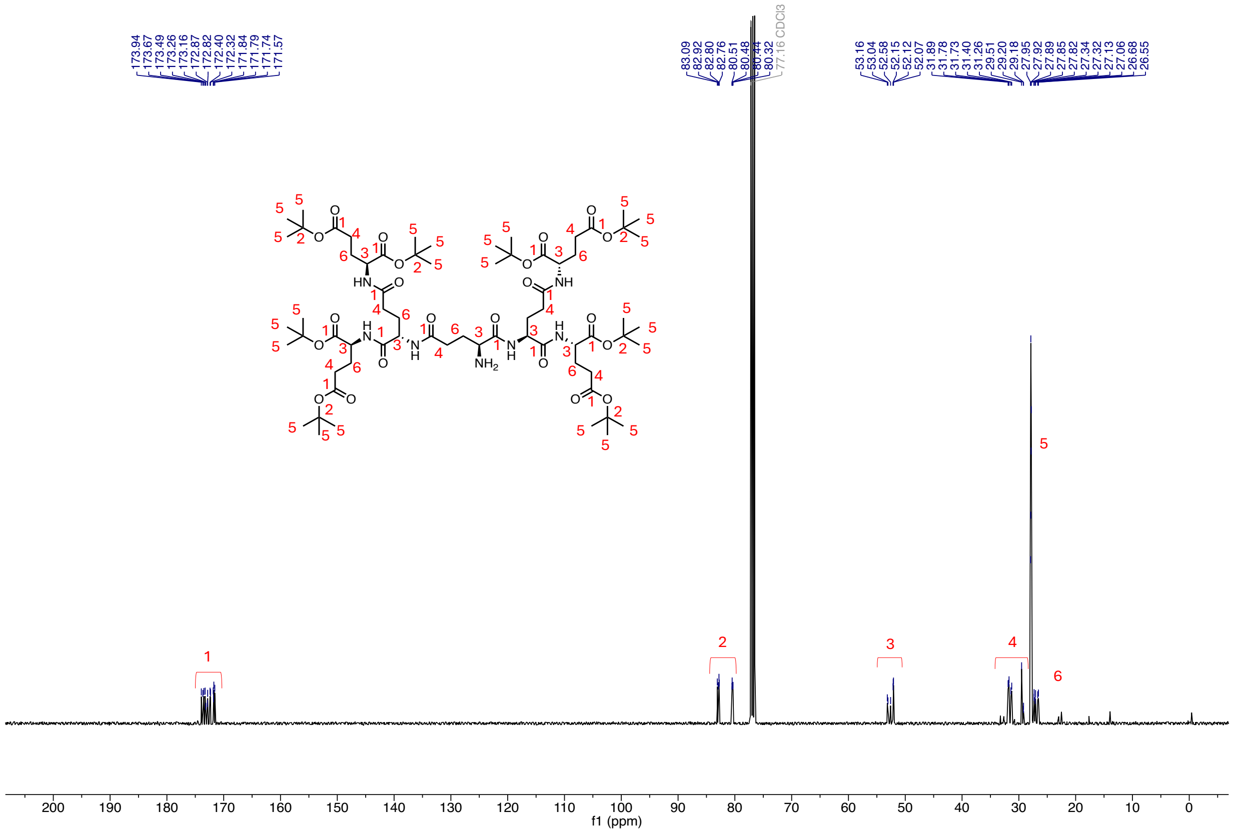

Fig. S26 101 MHz ^{13}C NMR spectrum of **12** in $CDCl_3$.

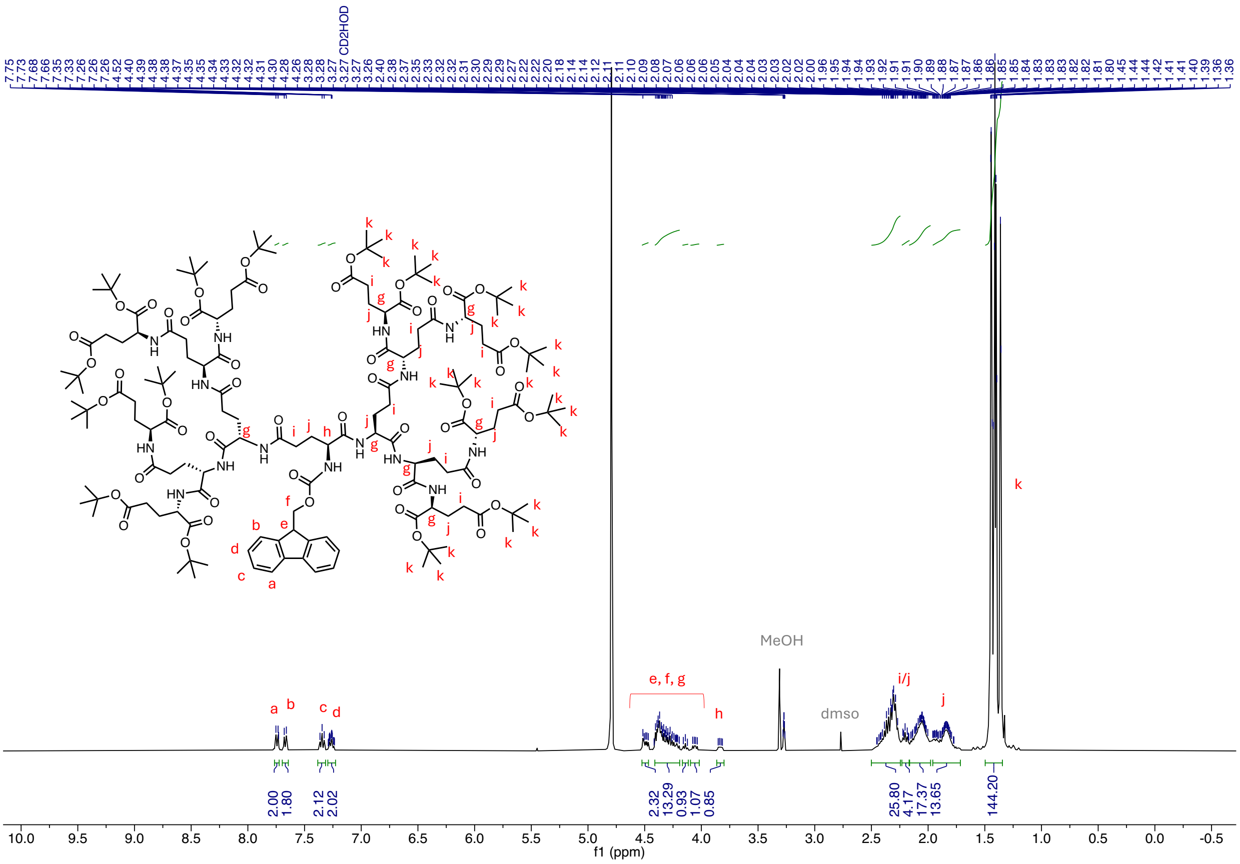

Fig. S27 400 MHz ^{1}H NMR spectrum of **13** in CD_3OD.

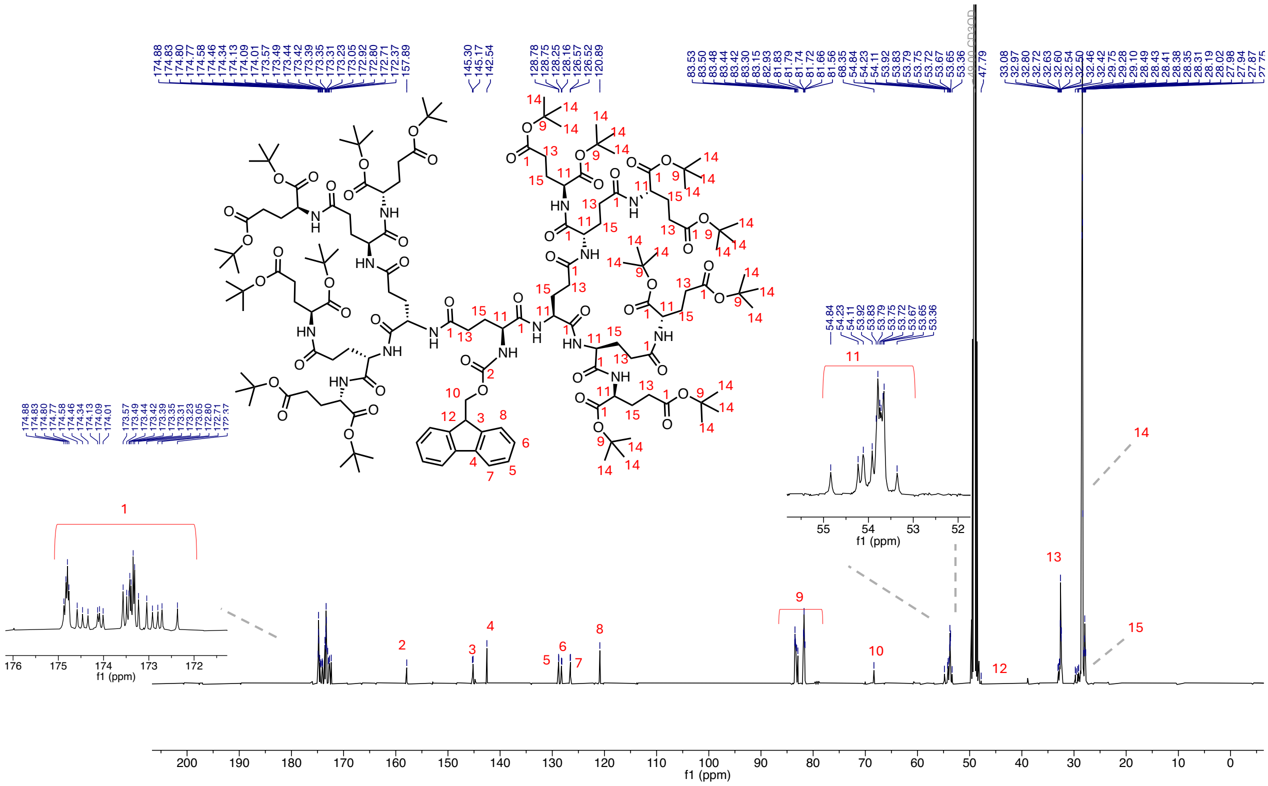

Fig. S28 101 MHz ^{13}C NMR spectrum of **13** in CD_3OD.

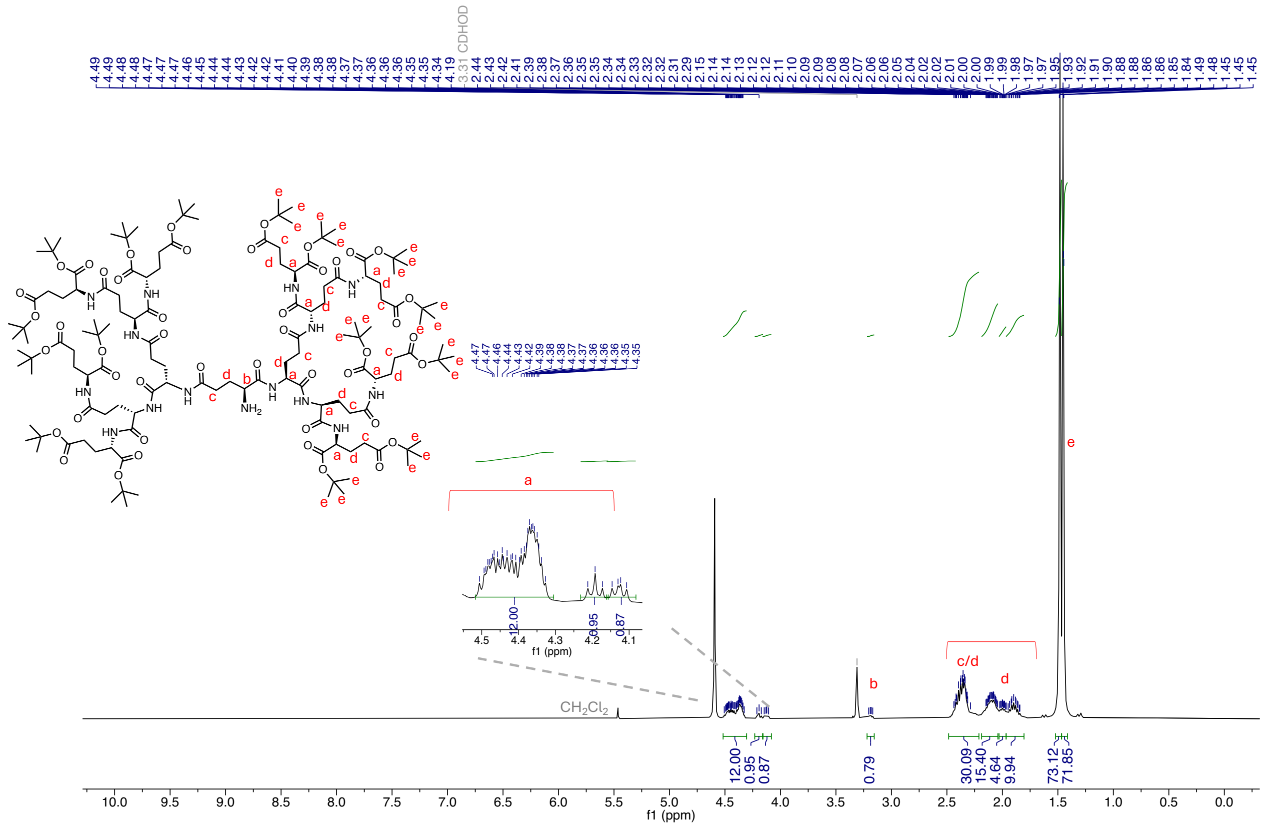

Fig. S29 400 MHz ^{1}H NMR spectrum of **14** in CD_3OD.

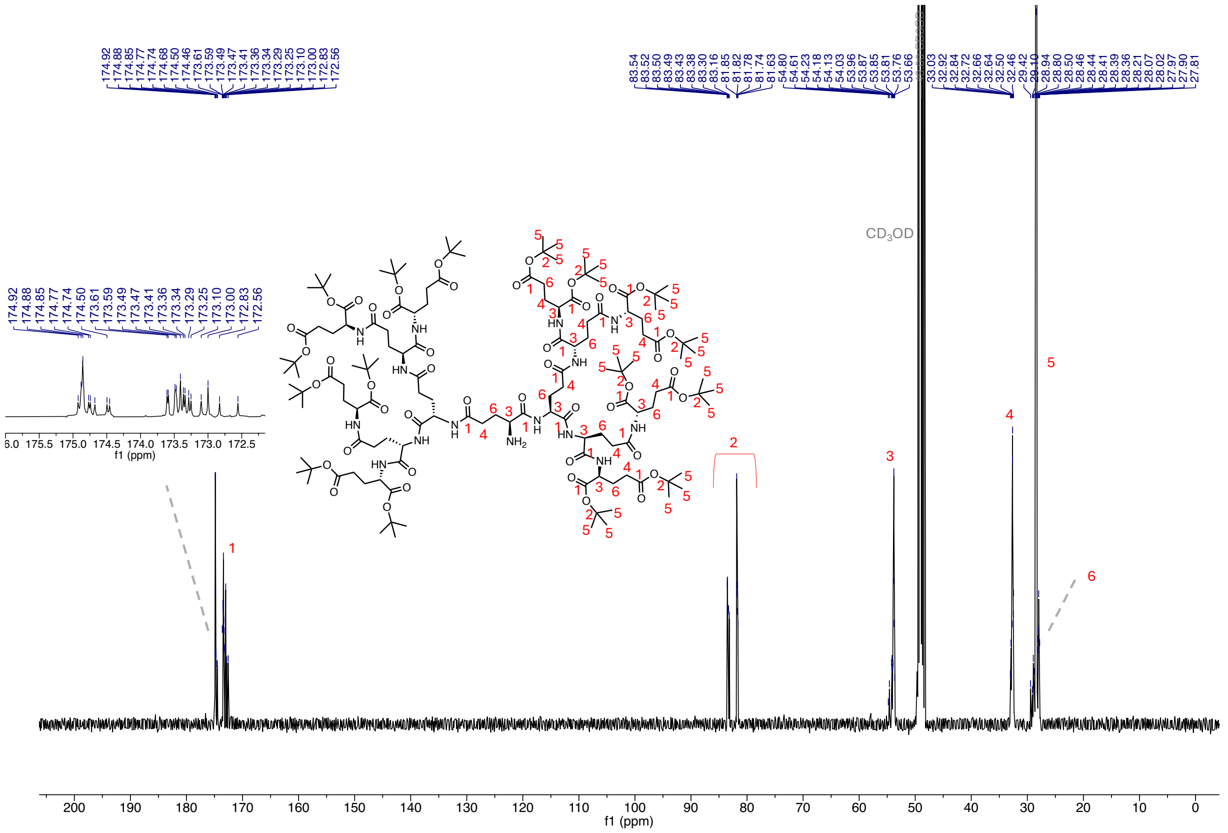

Fig. S30 101 MHz ^{13}C NMR spectrum of **14** in CD_3OD.

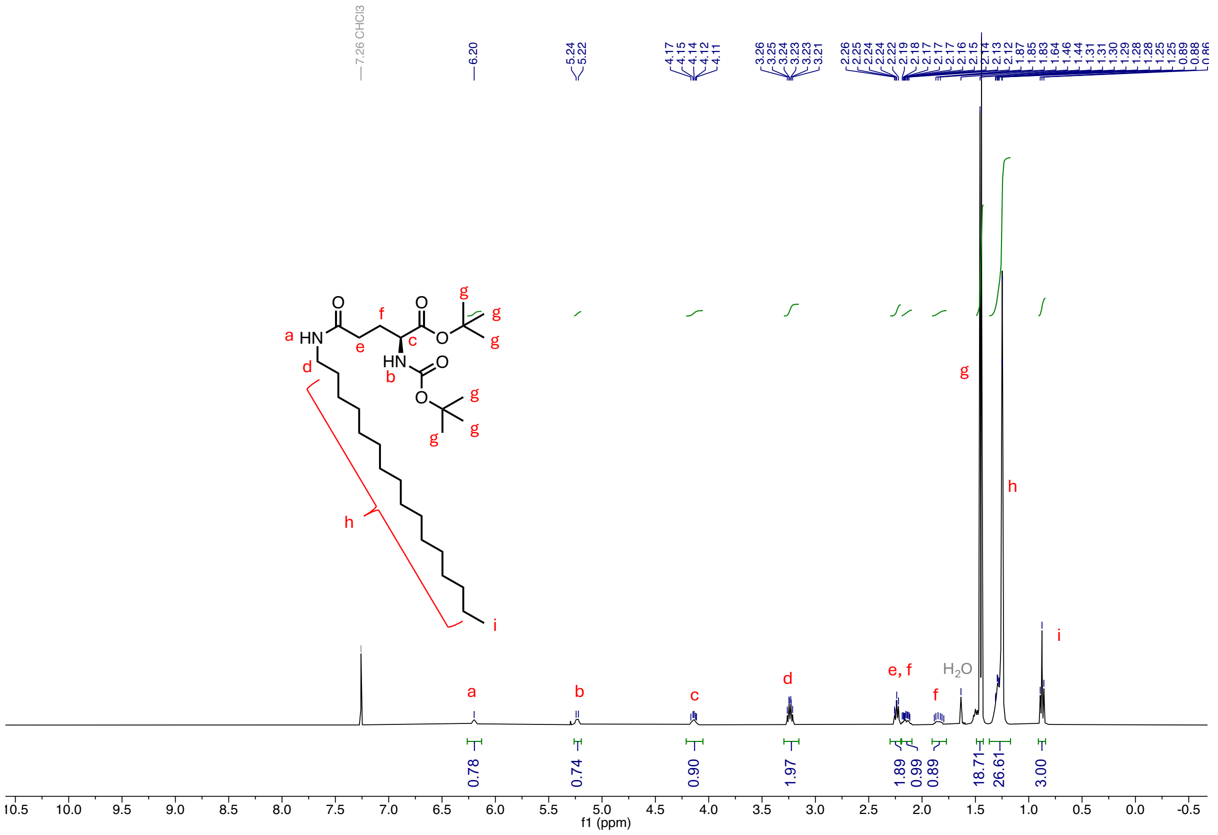

Fig. S31 400 MHz ^{1}H NMR spectrum of **15** in $CDCl_3$.

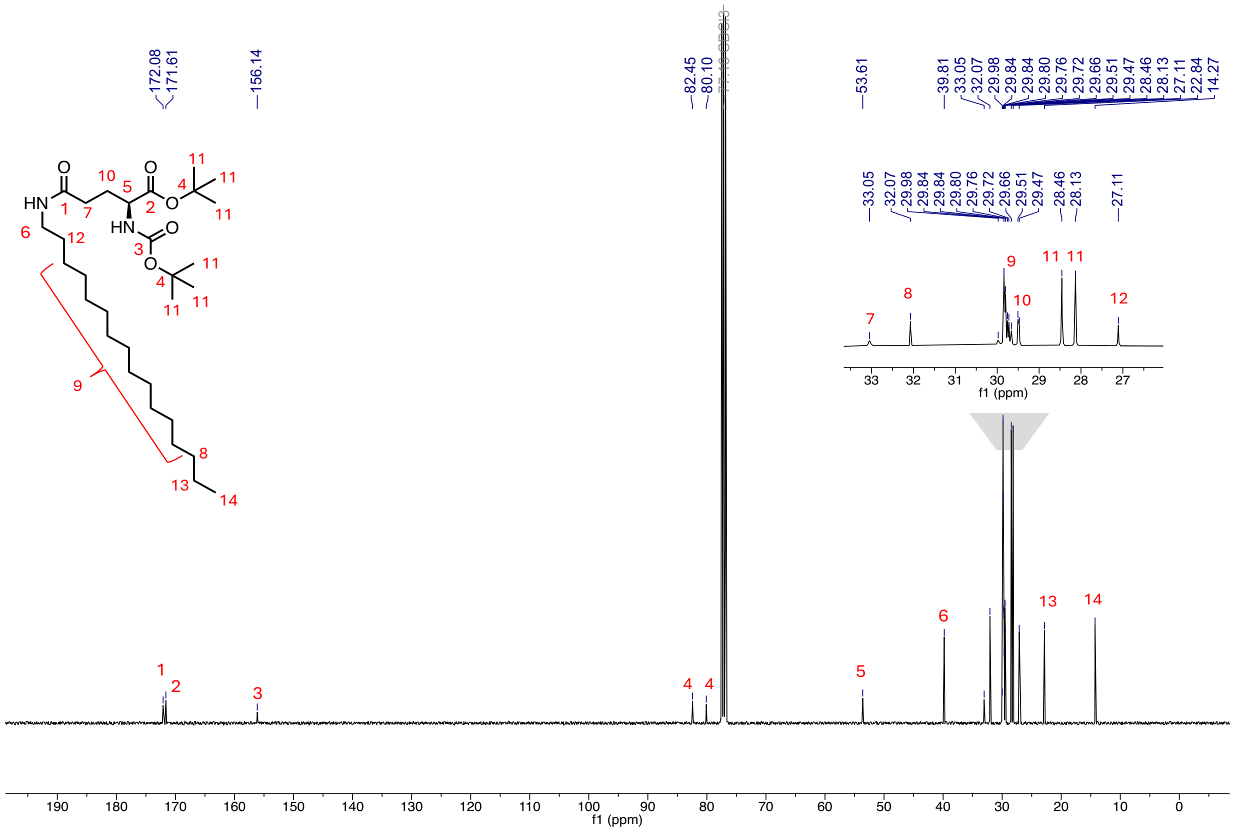

Fig. S32 101 MHz ^{13}C NMR spectrum of **15** in $CDCl_3$.

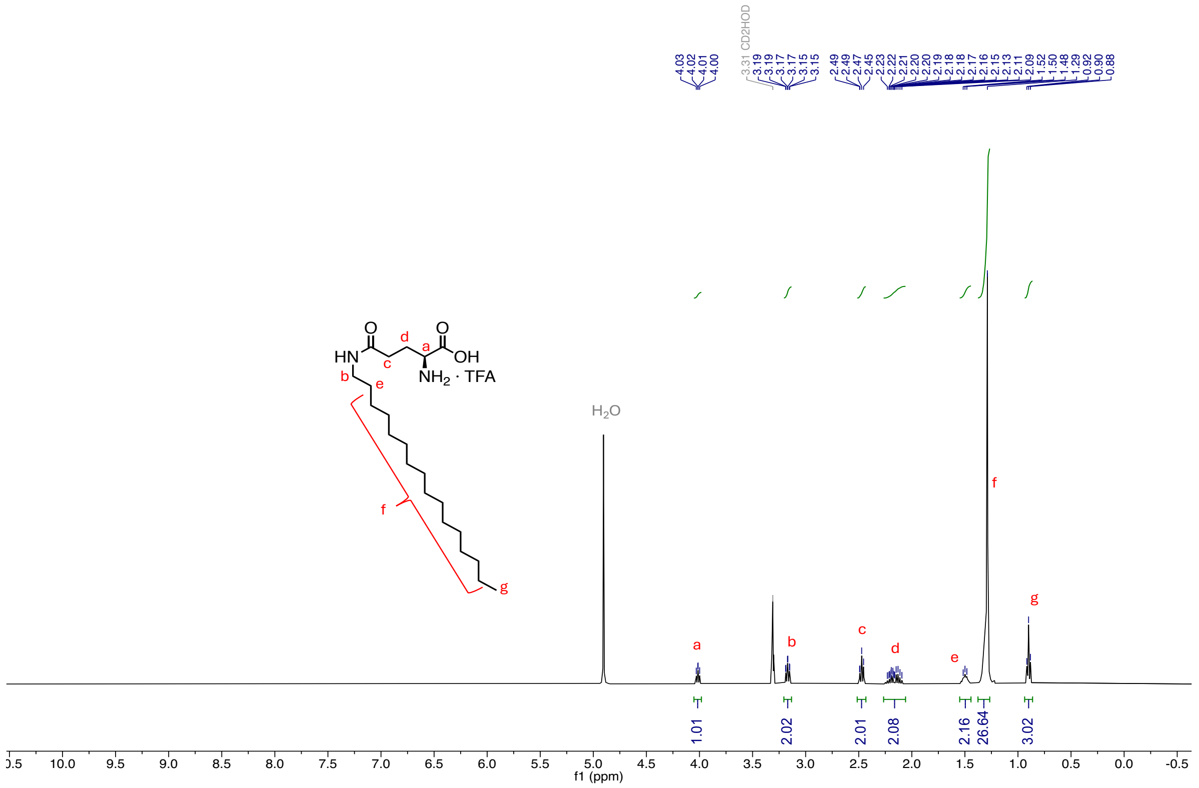

Fig. S33 400 MHz ^{1}H NMR spectrum of **20** in CD_3OD.

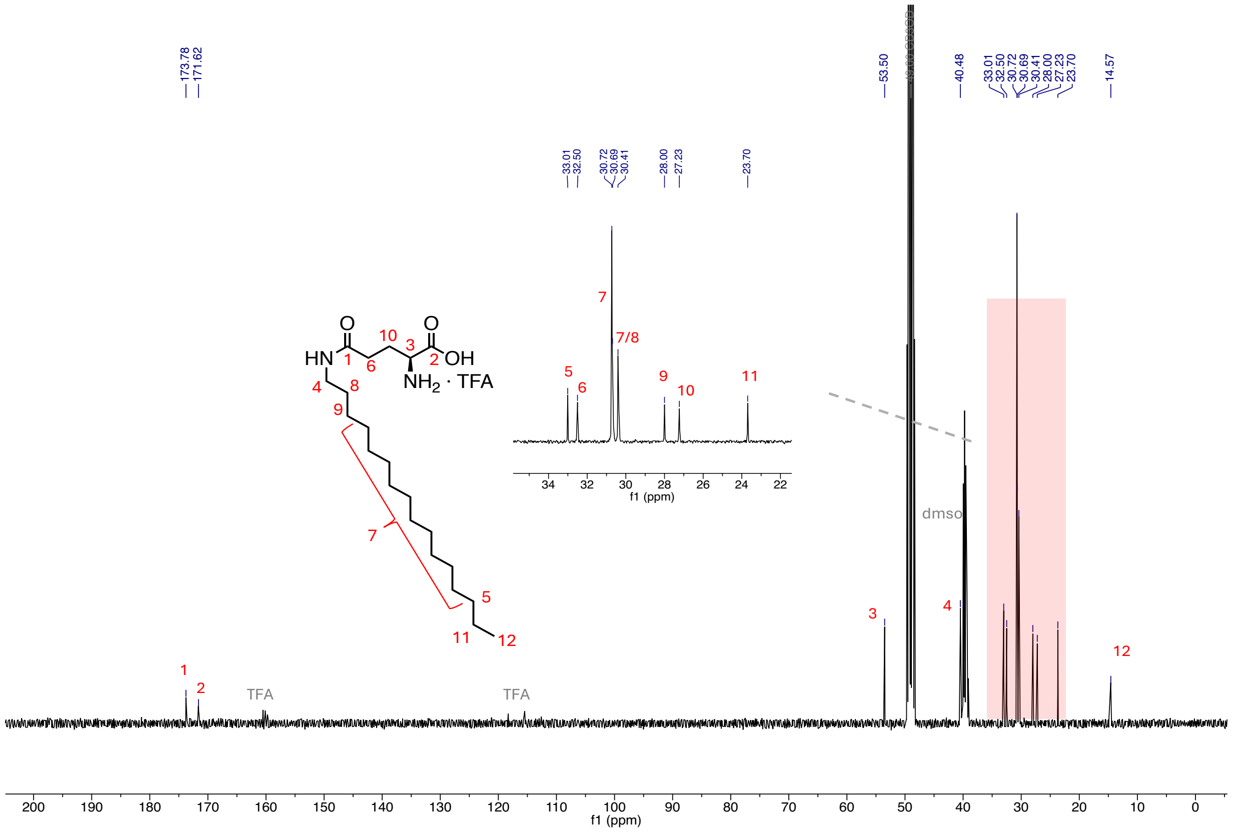

Fig. S34 101 MHz ^{13}C NMR spectrum of **20** in CD_3OD.

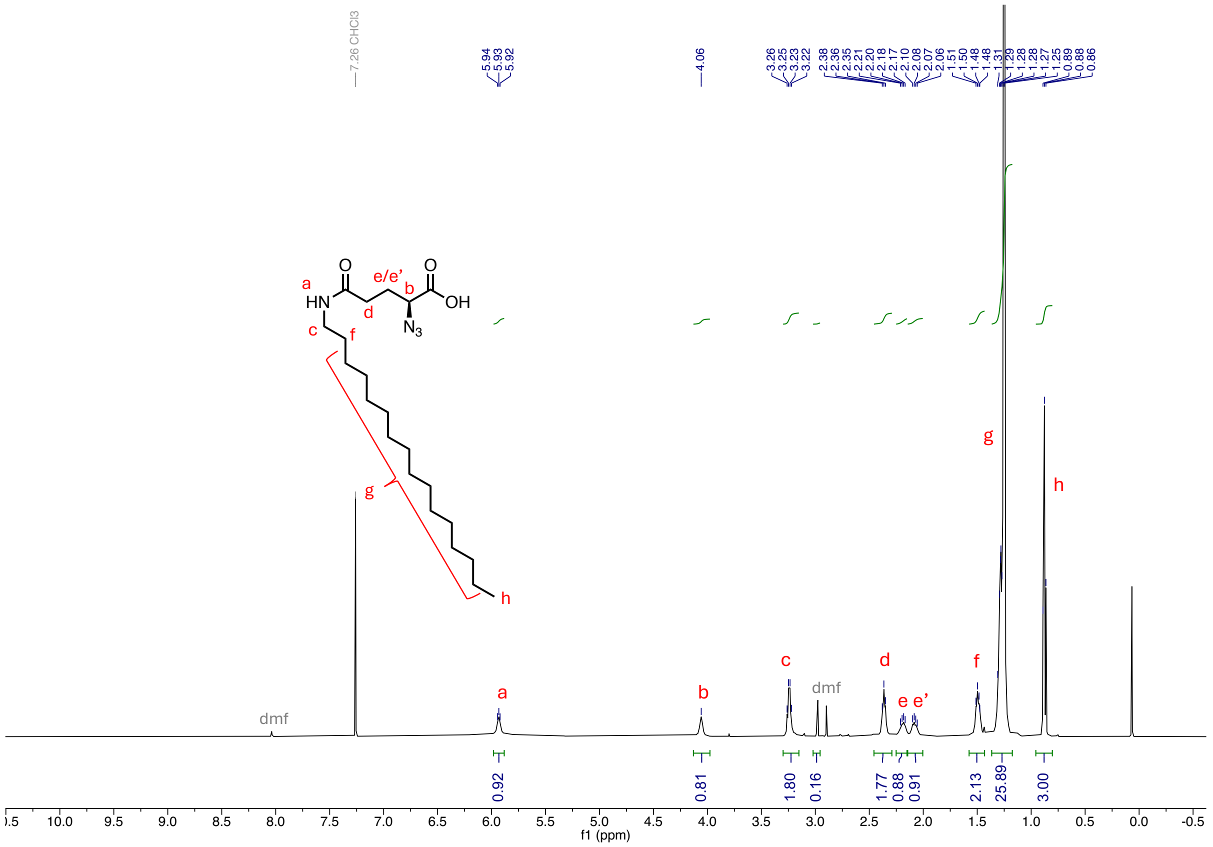

Fig. S35 500 MHz ^{1}H NMR spectrum of **23** in $CDCl_3$.

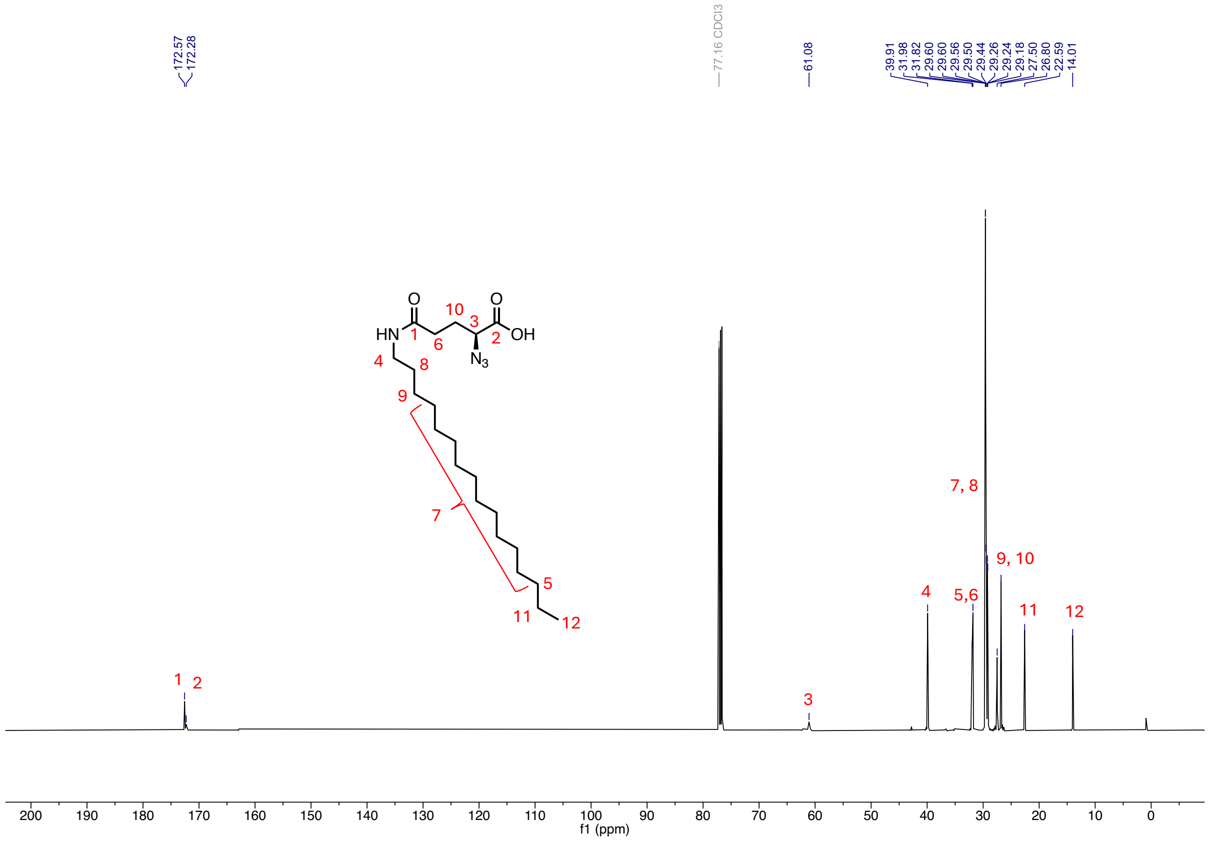

Fig. S36 126 MHz ^{13}C NMR spectrum of **23** in $CDCl_3$.

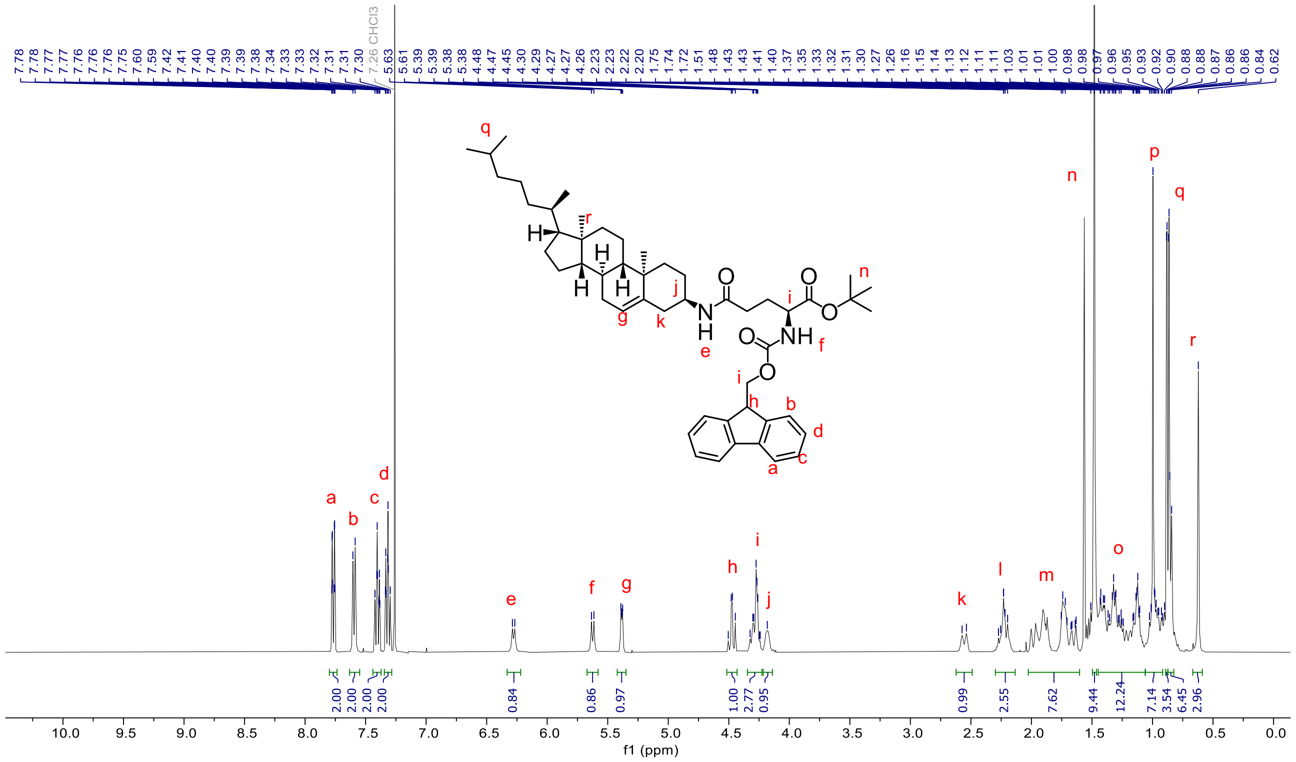

Fig. S37 400 MHz ^{1}H NMR spectrum of **26** in $CDCl_3$.

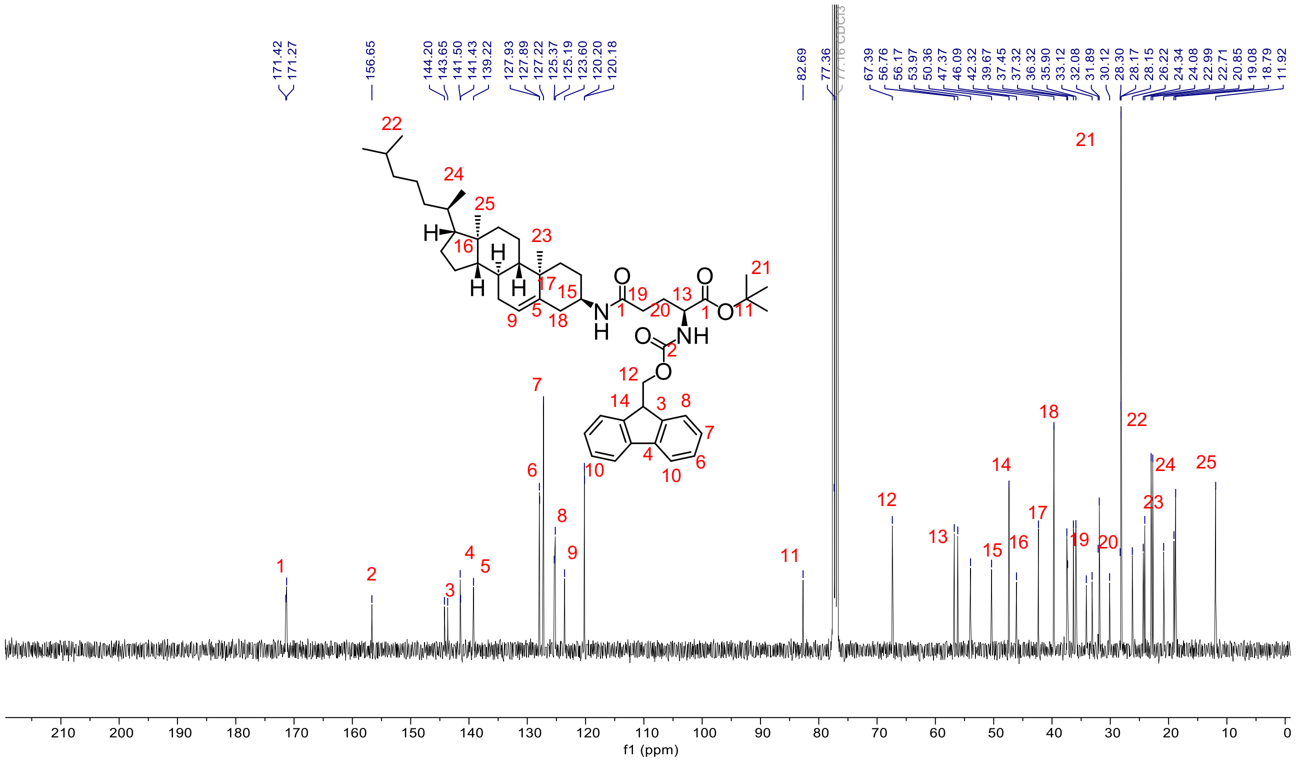

Fig. S38 101 MHz ^{13}C NMR spectrum of **26** in $CDCl_3$.

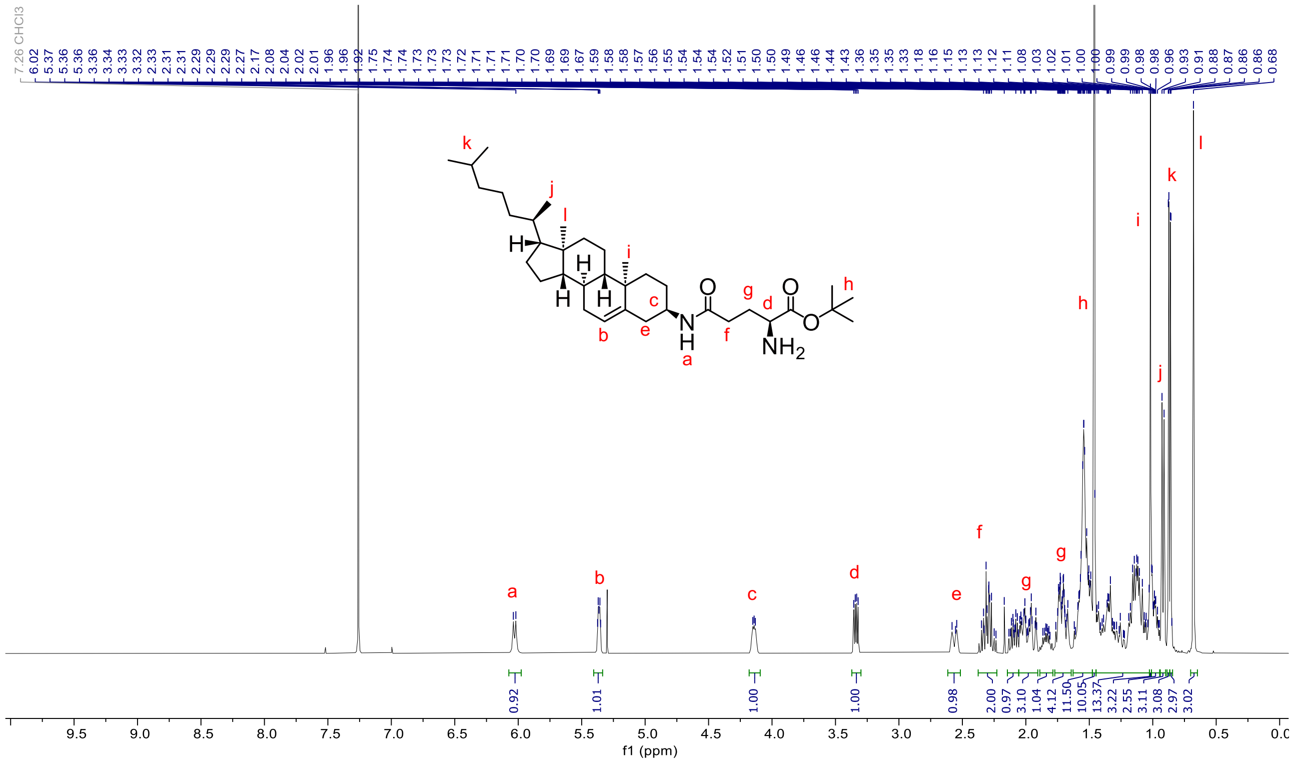

Fig. S39 400 MHz ^{1}H NMR spectrum of **28** in $CDCl_3$.

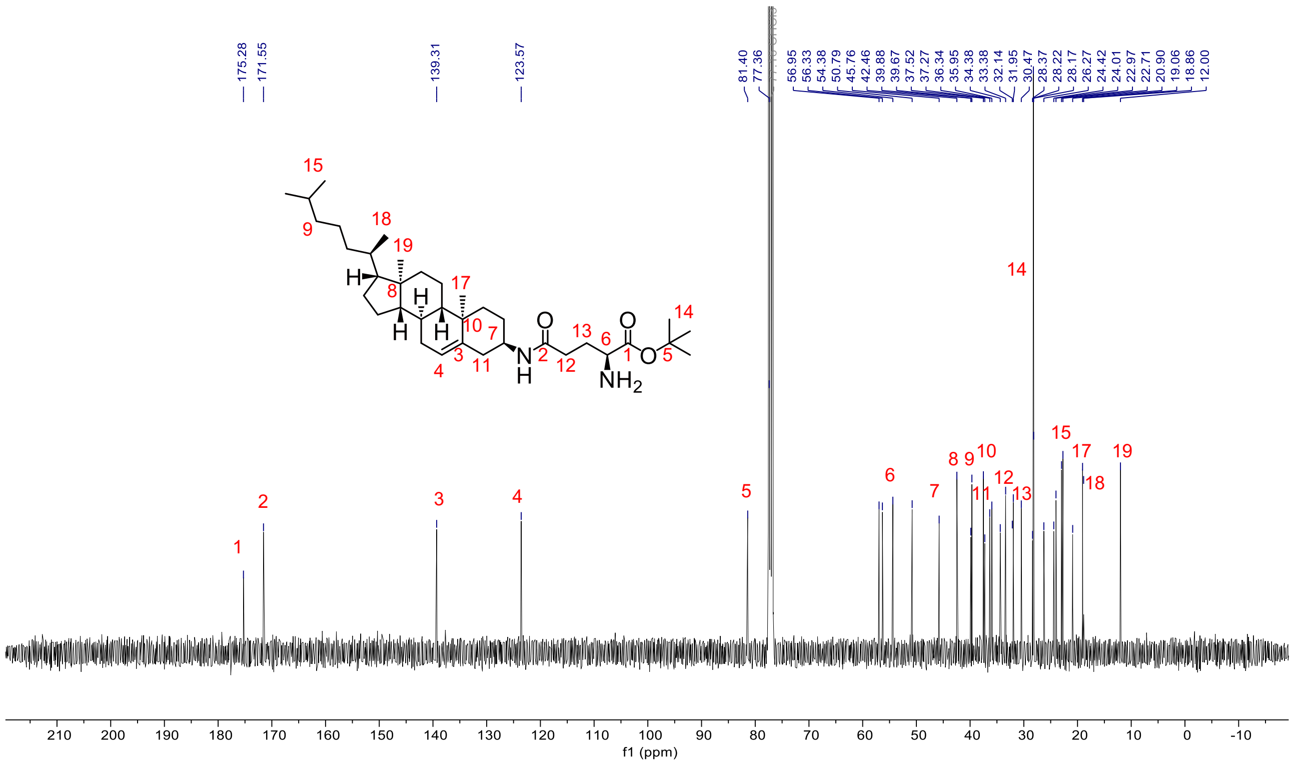

Fig. S40 101 MHz ^{13}C NMR spectrum of **28** in $CDCl_3$.

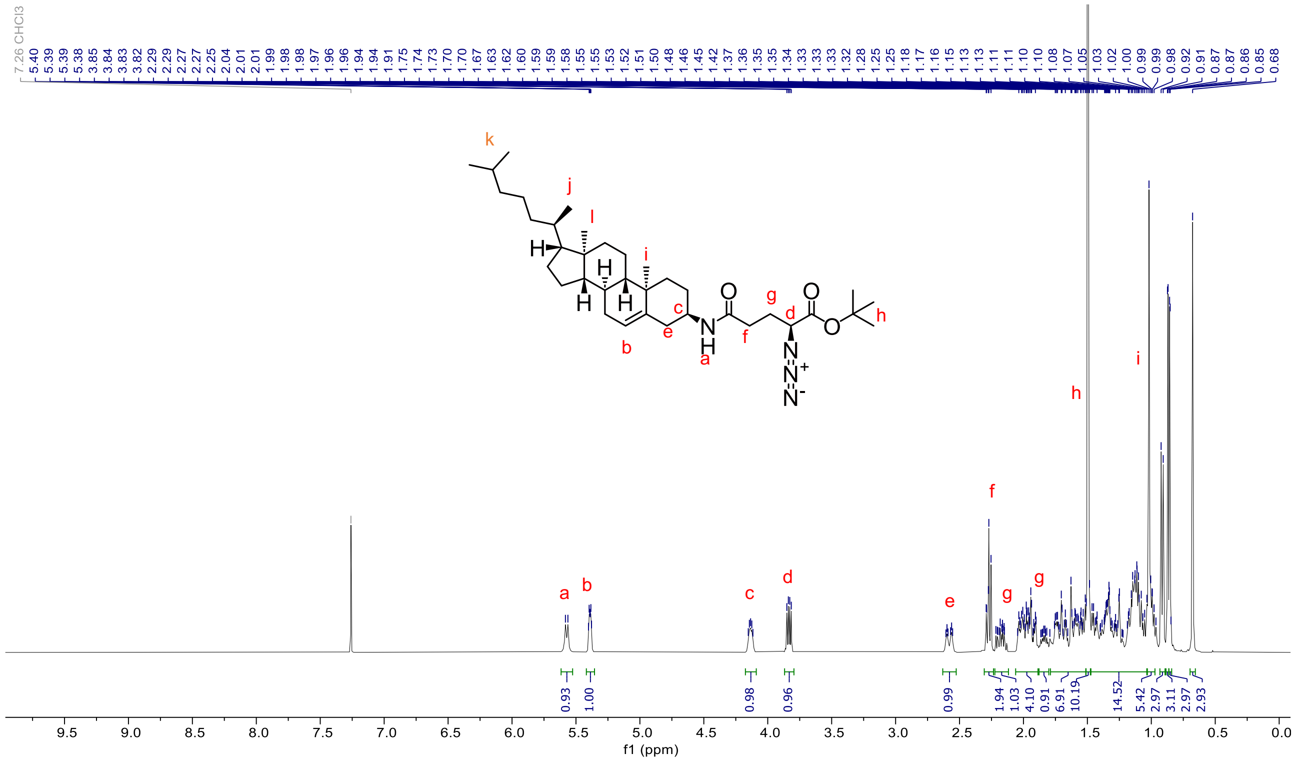

Fig. S41 400 MHz ^{1}H NMR spectrum of **29** in $CDCl_3$.

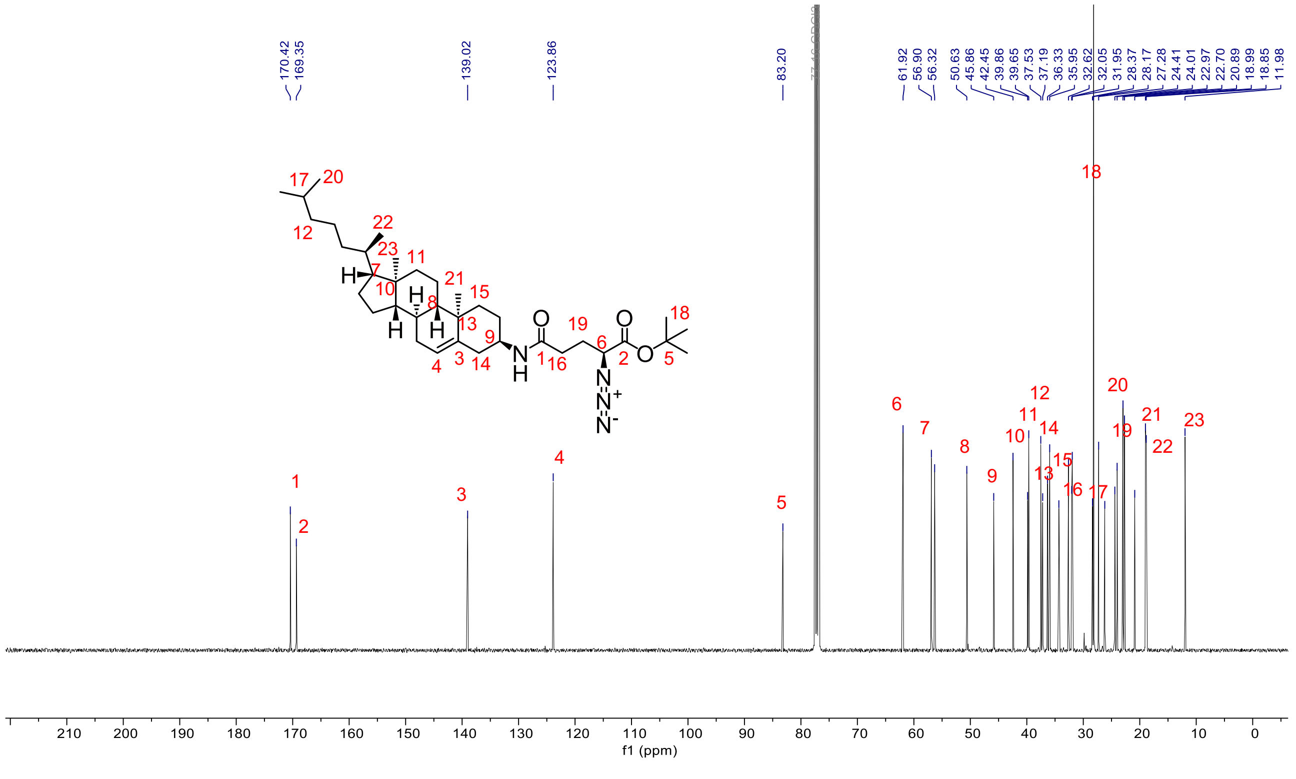

Fig. S42 101 MHz ^{13}C NMR spectrum of **29** in $CDCl_3$.

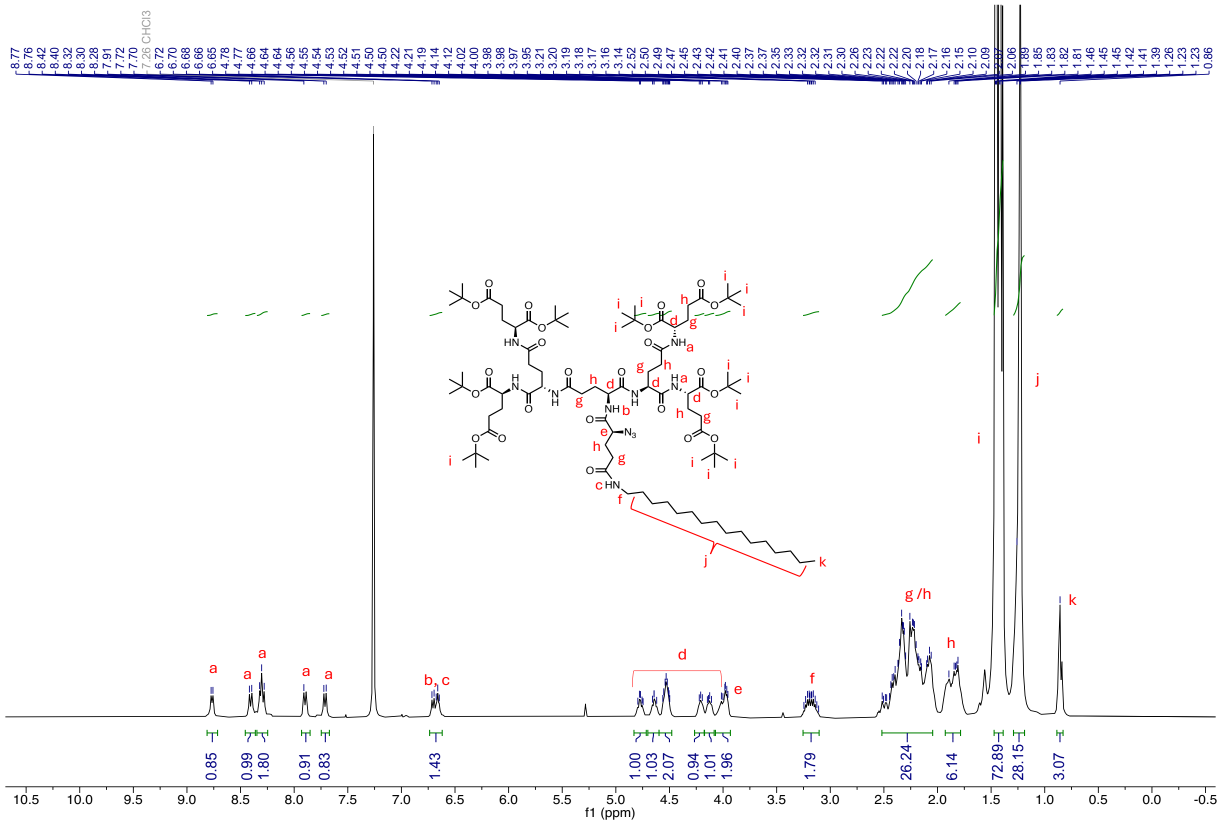

Fig. S43 400 MHz ^{1}H NMR spectrum of **31** in $CDCl_3$.

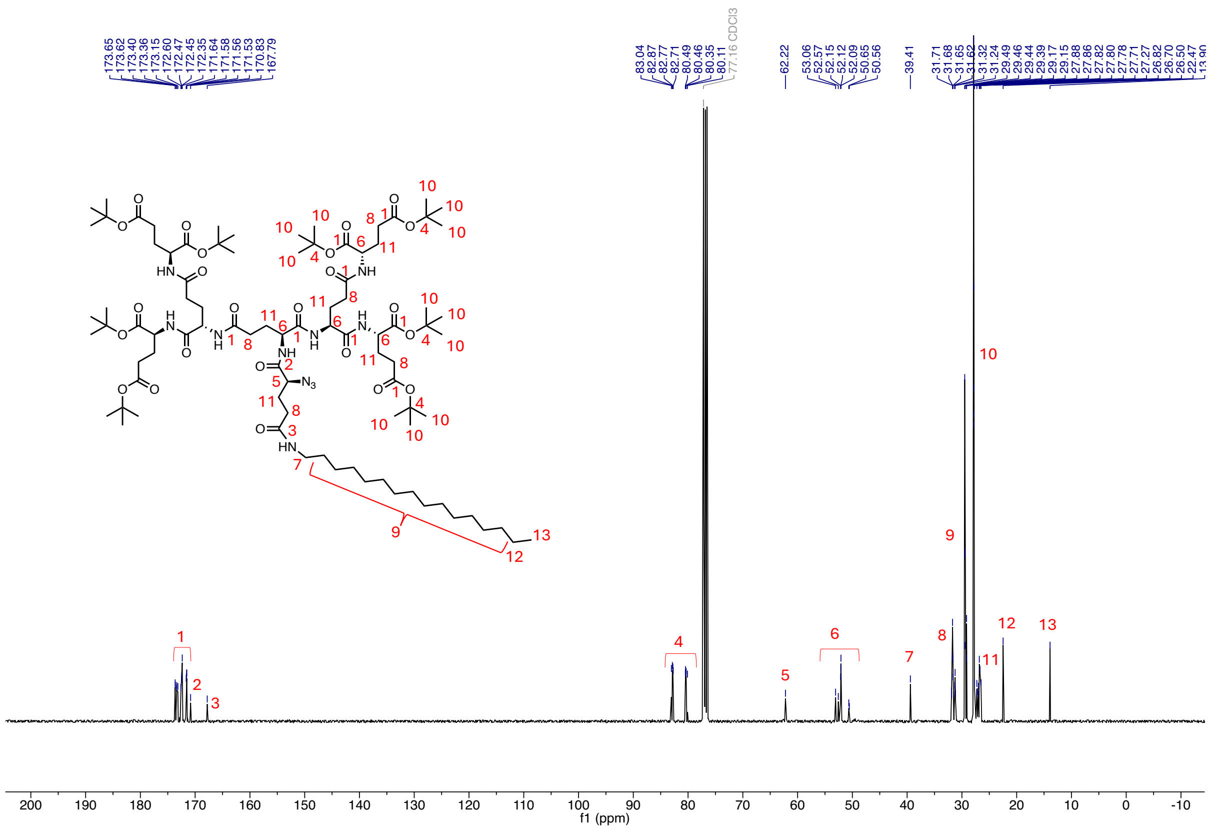

Fig. S44 101 MHz ^{13}C NMR spectrum of **31** in $CDCl_3$.

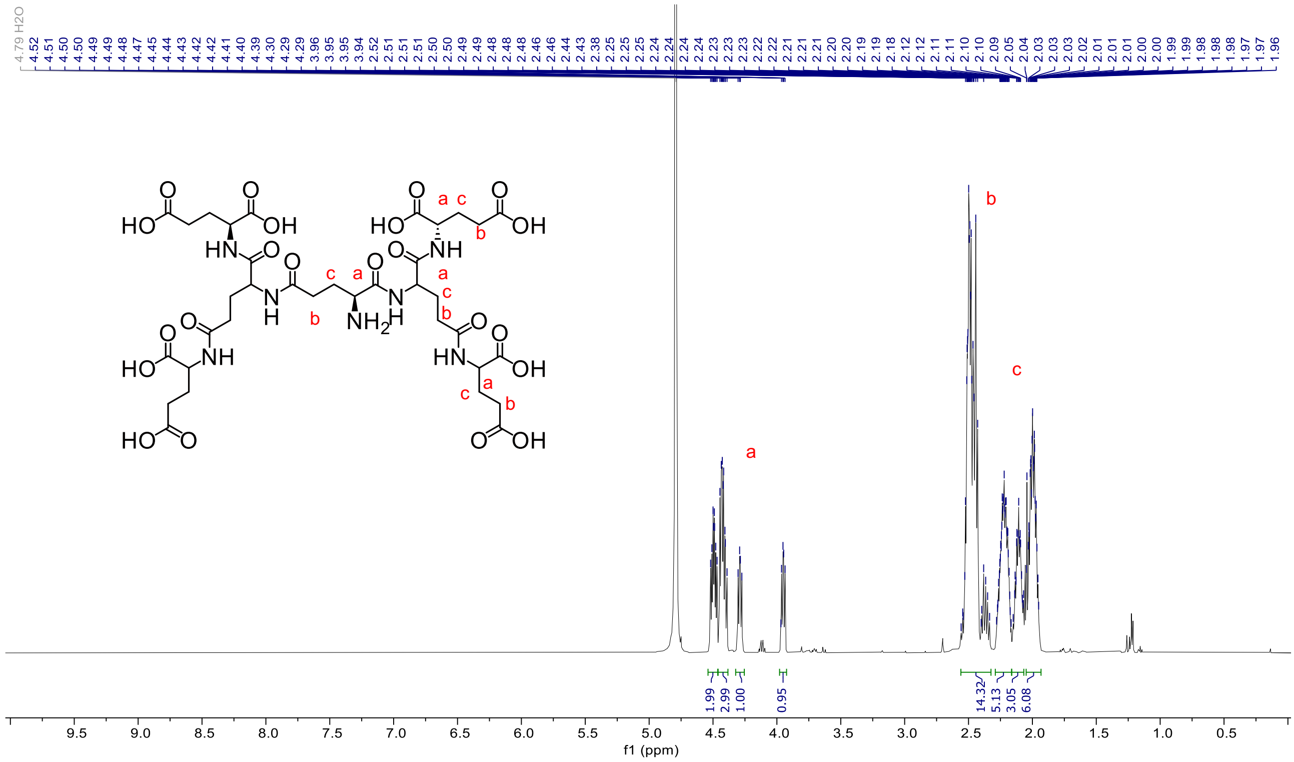

Fig. S45 500 MHz ^{1}H NMR spectrum of **32** in D_2O.

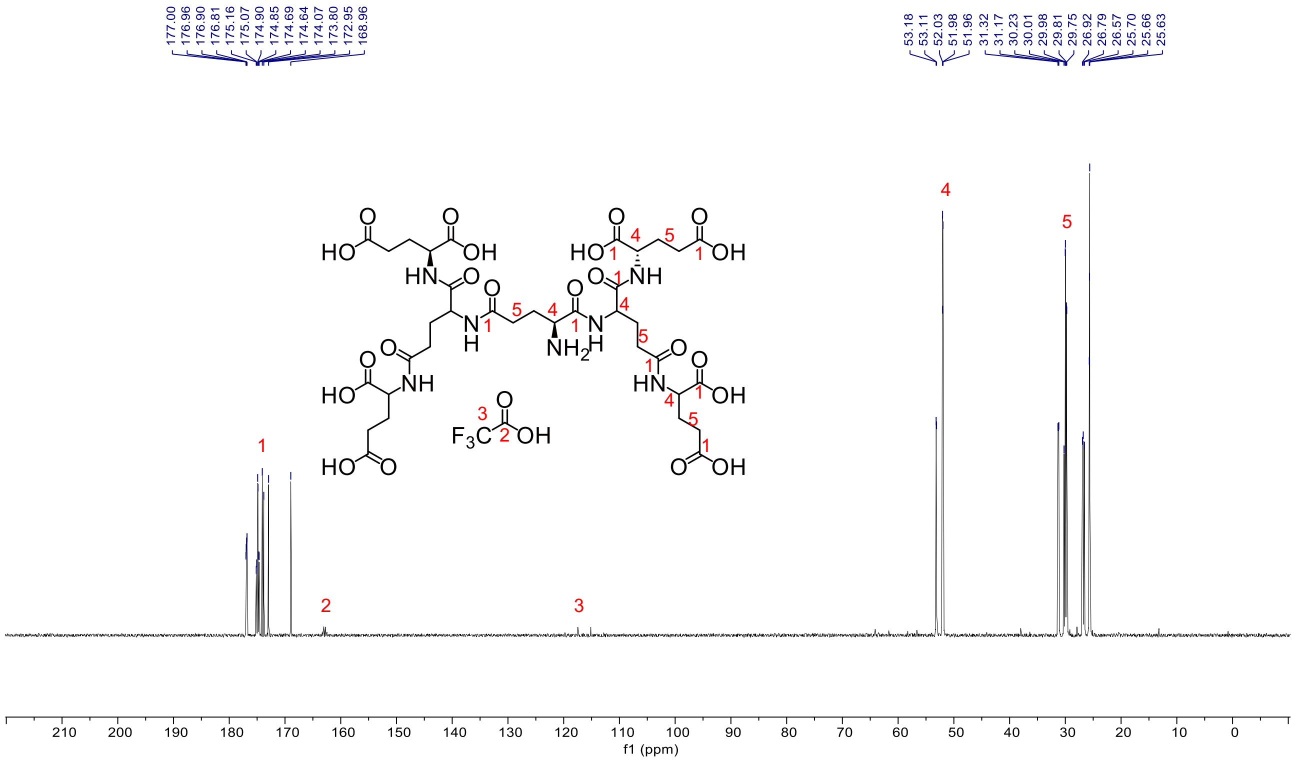

Fig. S46 126 MHz ^{13}C NMR spectrum of **32** in D_2O.

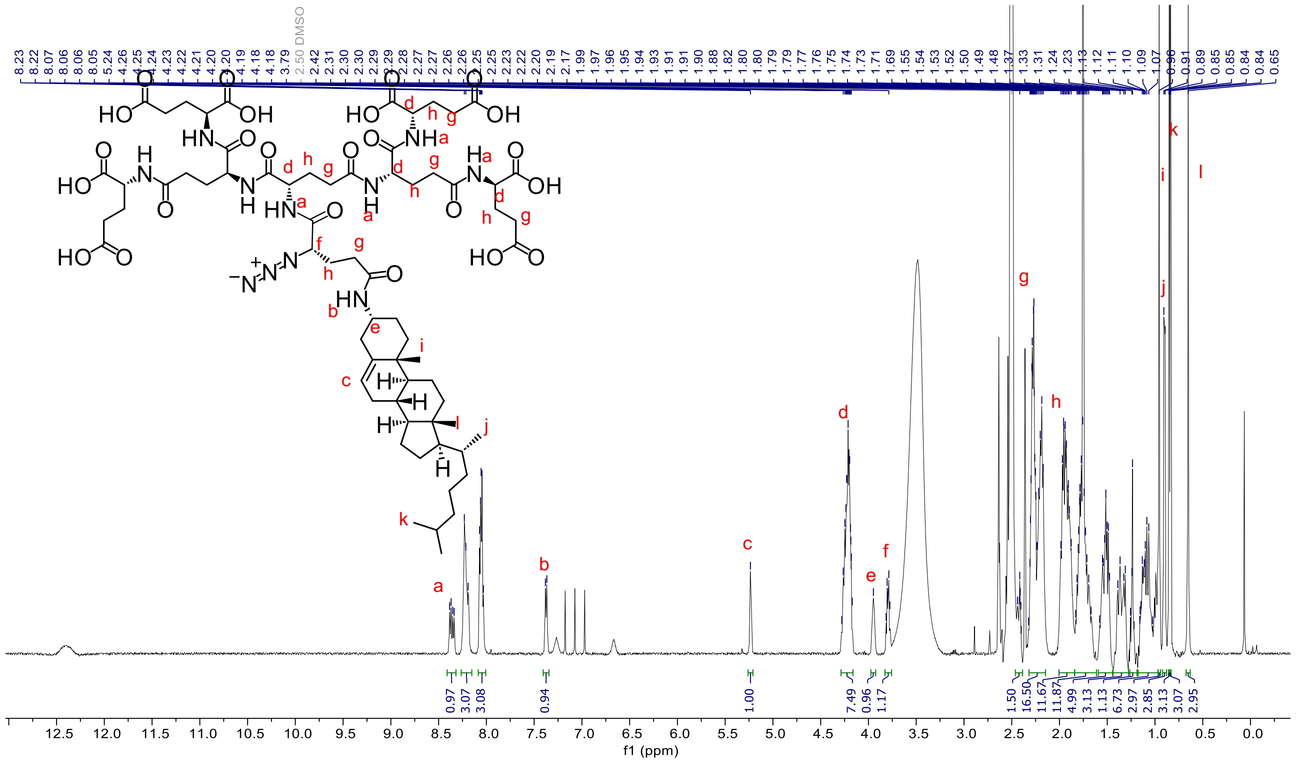

Fig. S47 500 MHz ^{1}H NMR spectrum of **33** in DMSO-d_6.

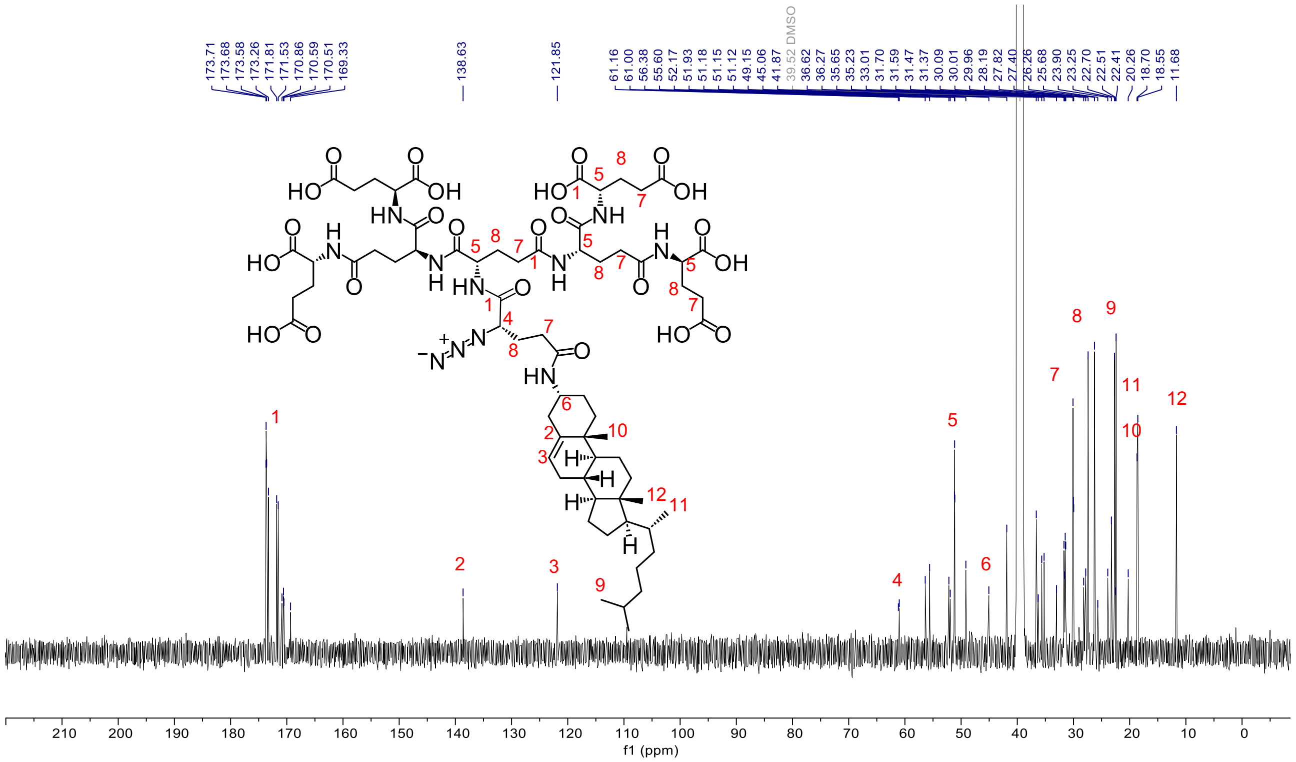

Fig. S48 126 MHz ^{13}C NMR spectrum of **33** in DMSO-d_6.

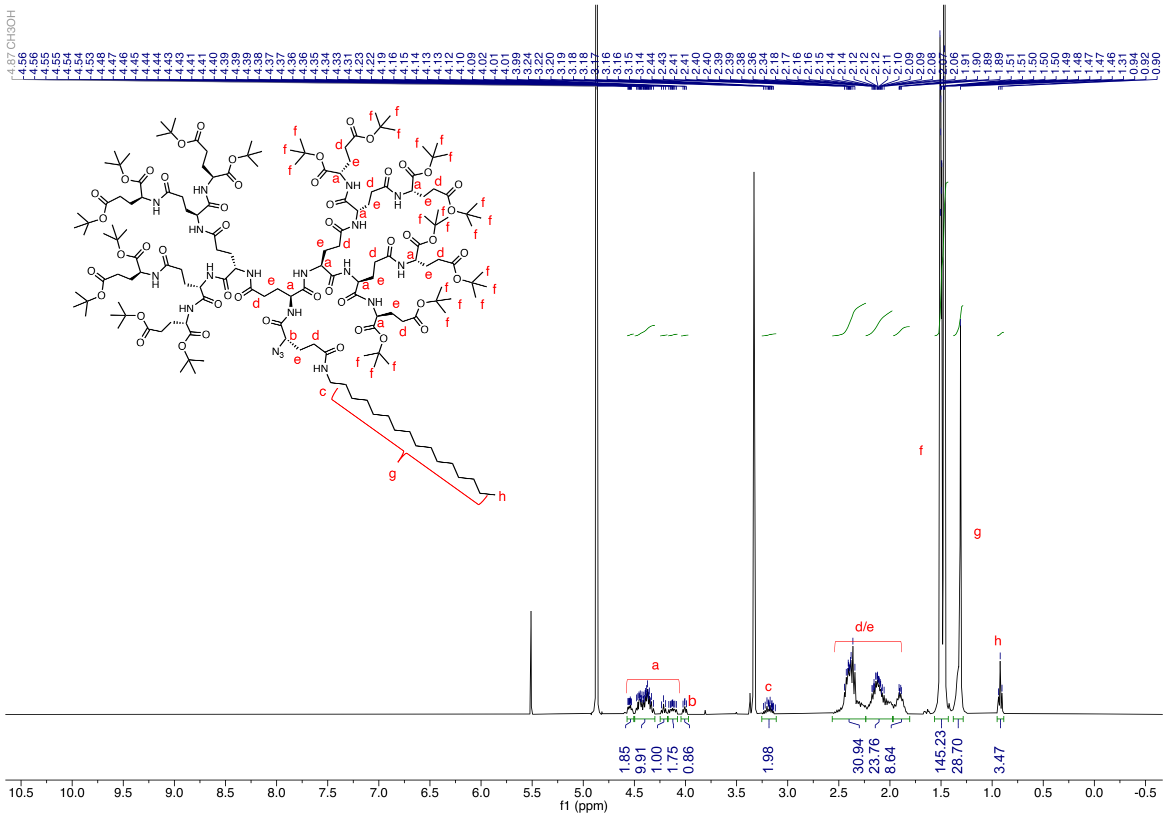

Fig. S49 400 MHz ^{1}H NMR spectrum of **34** in CD_3OD.

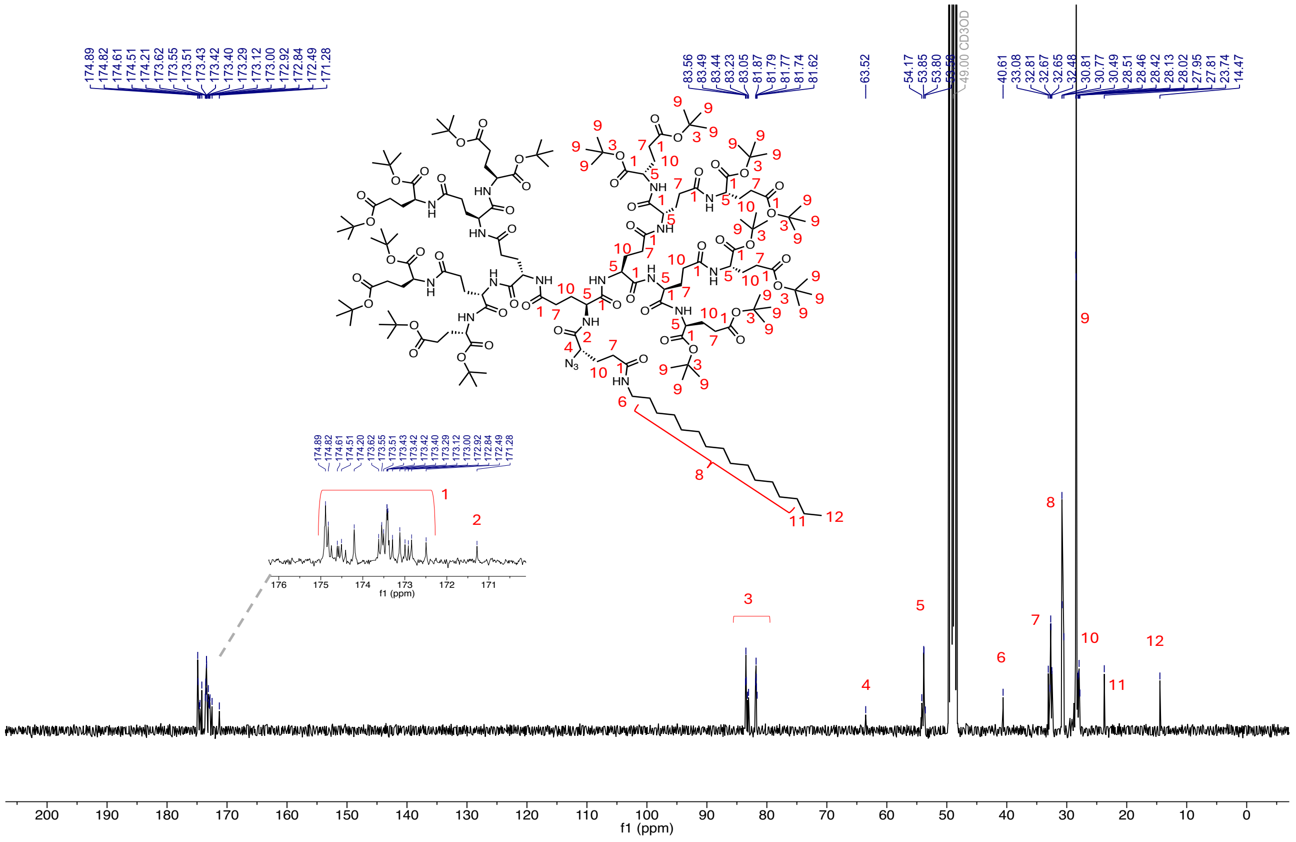

Fig. S50 101 MHz ^{13}C NMR spectrum of **34** in CD_3OD.

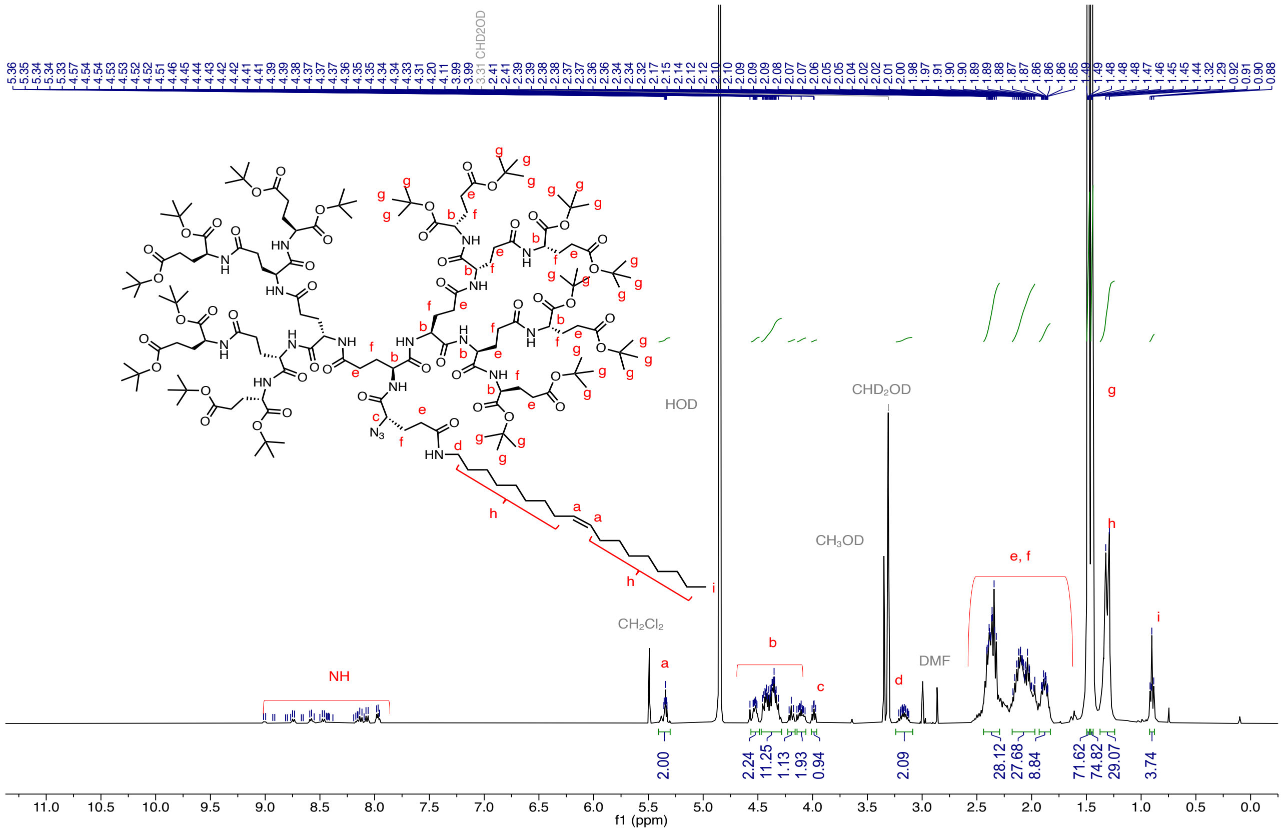

Fig. S51 400 MHz ^{1}H NMR spectrum of **35** in CD_3OD.

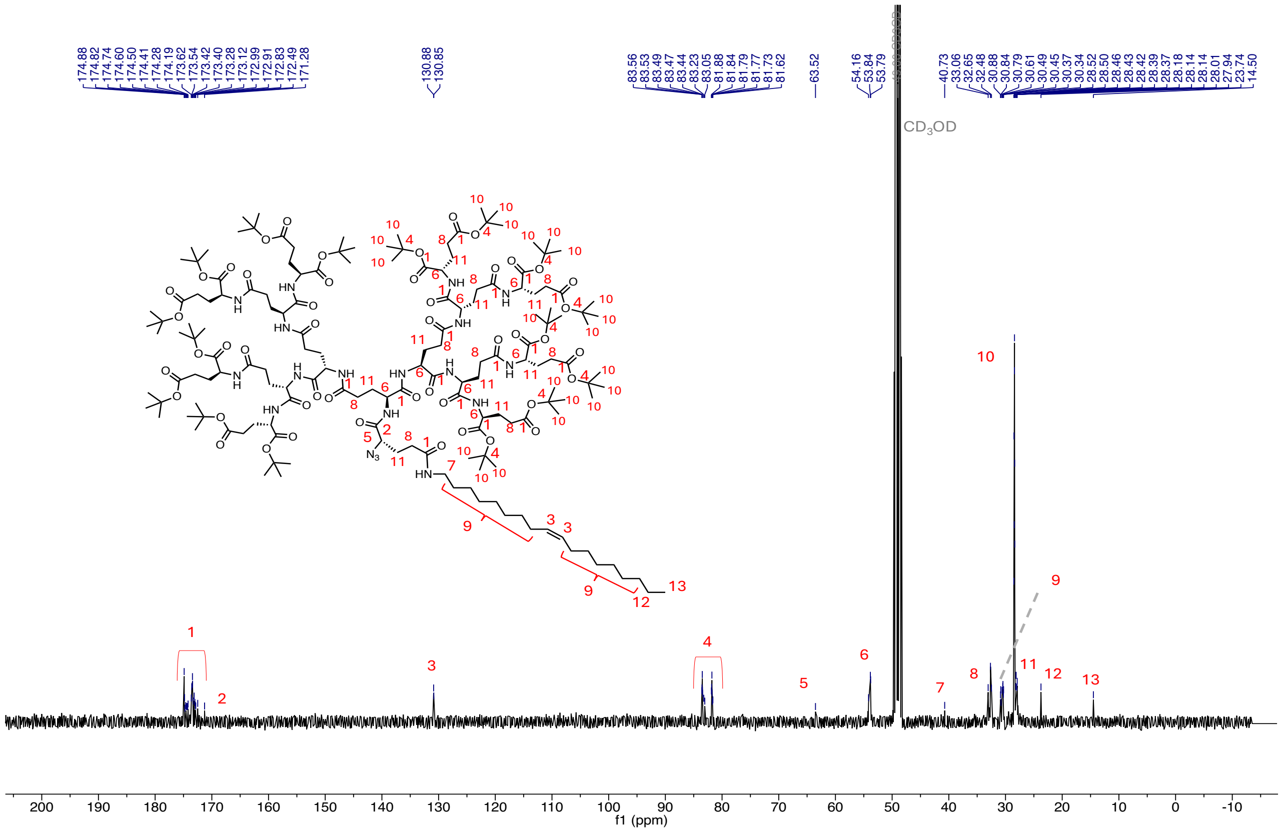

Fig. S52 101 MHz ^{13}C NMR spectrum of **35** in CD_3OD.

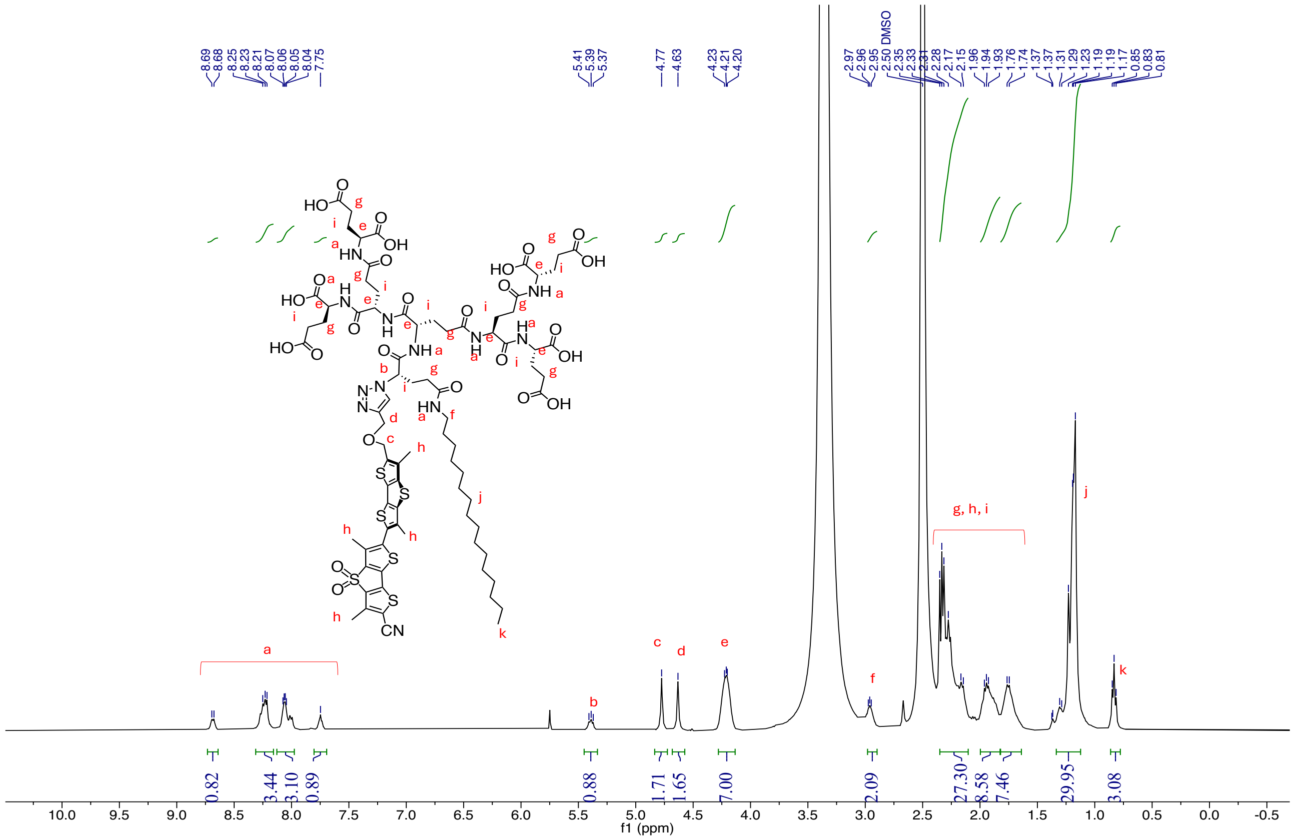

Fig. S53 500 MHz ^1H NMR spectrum of **3** in DMSO-d_6.

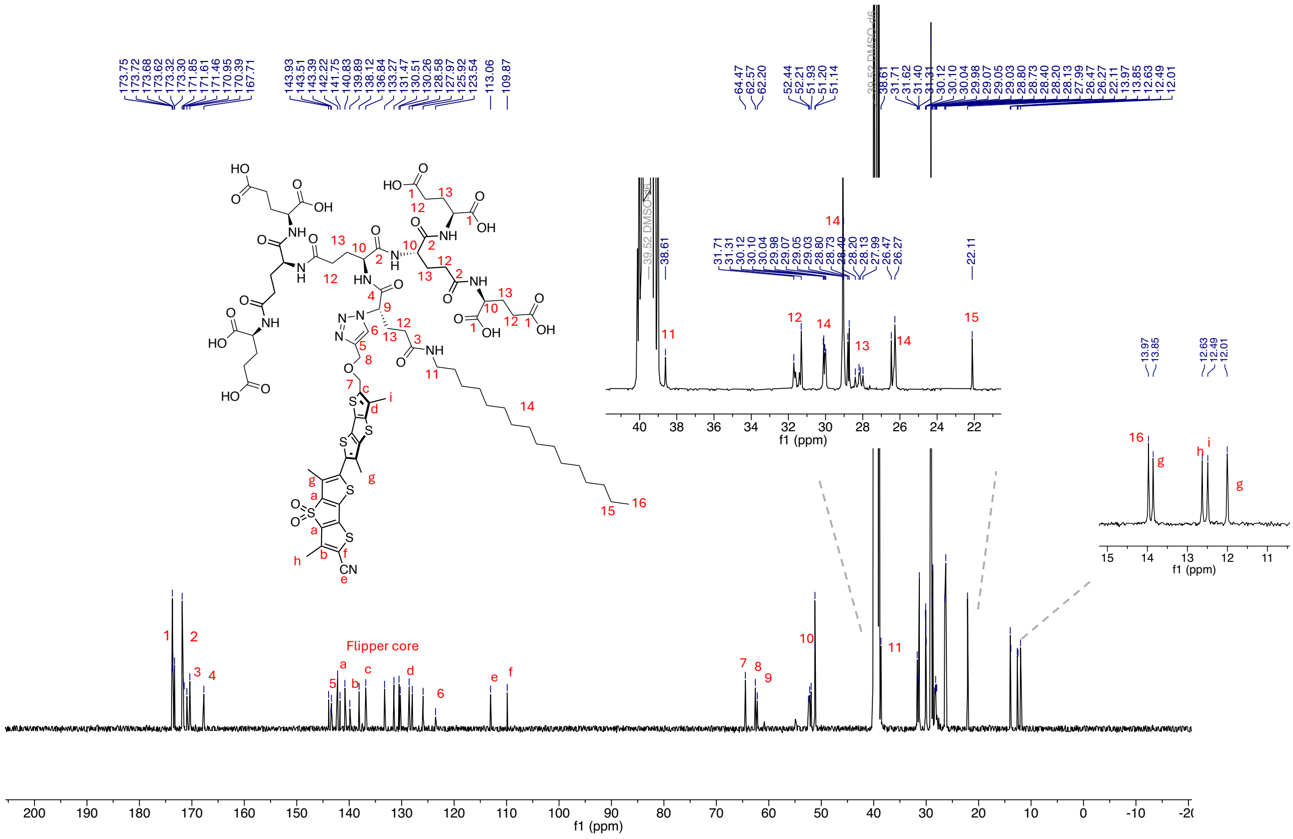

Fig. S54 126 MHz ^{13}C NMR spectrum of **3** in DMSO-d_6.

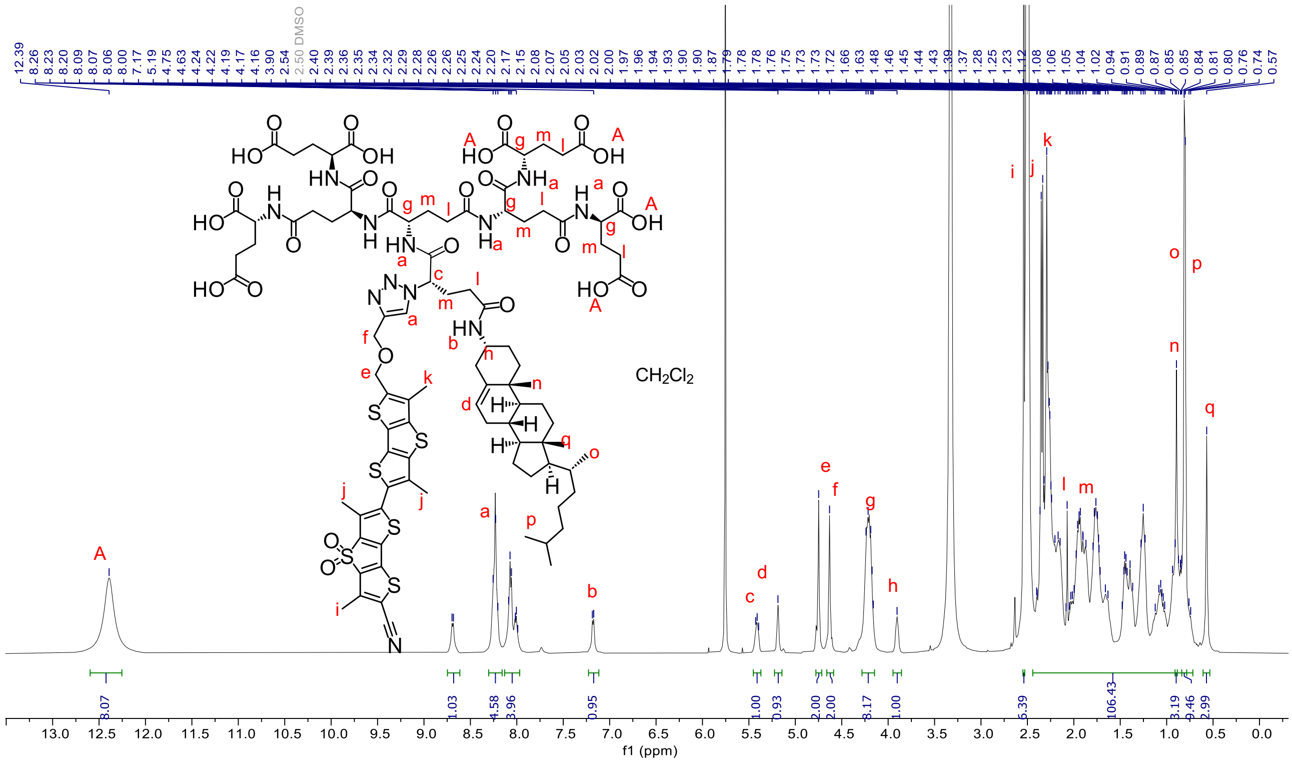

Fig. S55 500 MHz ^{1}H-NMR spectrum of **6** in DMSO-d_6.

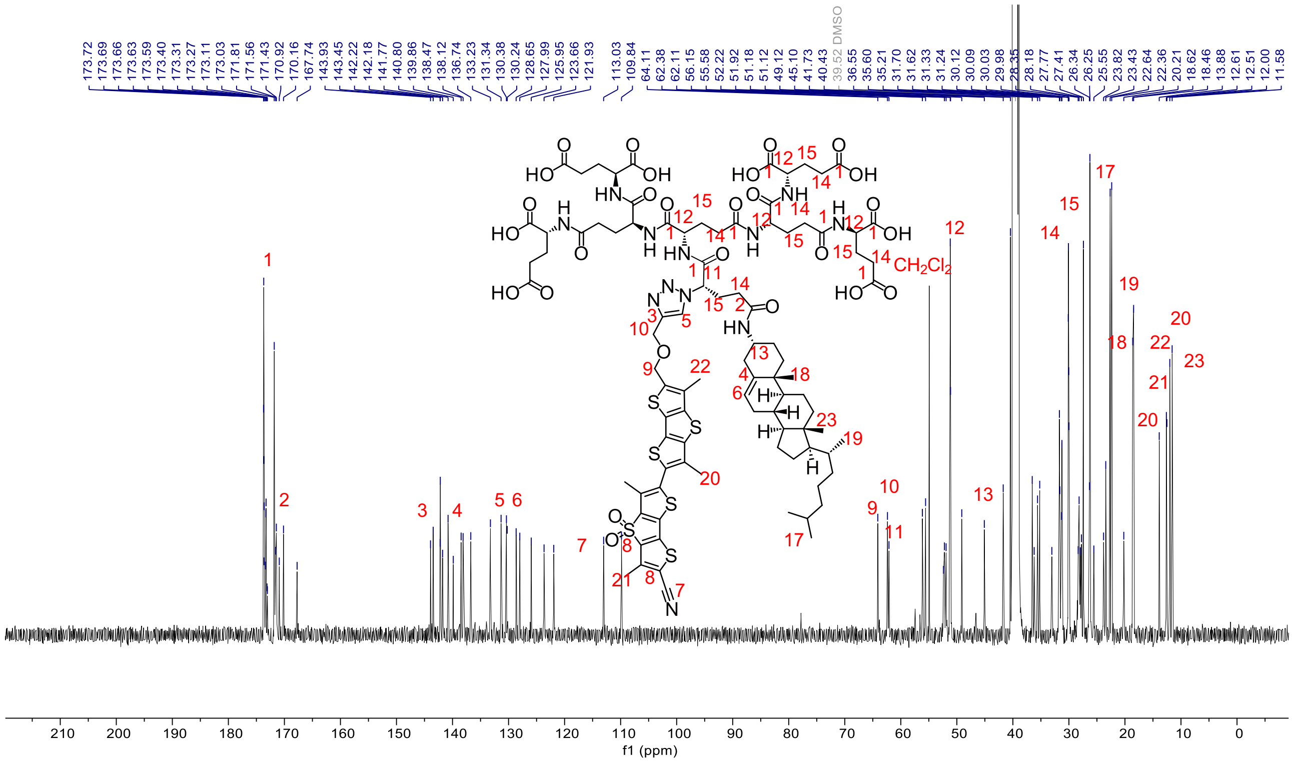

Fig. S56 126 MHz ^{13}C-NMR spectrum of **6** in DMSO-d_6.
